# Heterogeneity of radial spoke components in *Tetrahymena* cilia

**DOI:** 10.1007/s00018-025-05871-x

**Published:** 2025-08-31

**Authors:** Marta Bicka, Avrin Ghanaeian, Corbin Black, Ewa Joachimiak, Anna Osinka, Sumita Majhi, Anna Konopka, Ewa Bulska, Khanh Huy Bui, Dorota Wloga

**Affiliations:** 1 Laboratory of Cytoskeleton and Cilia Biology, Nencki Institute of Experimental Biology of Polish Academy of Sciences, 3 Pasteur Street, Warsaw, 02-093 Poland; 2 Faculty of Chemistry, University of Warsaw, 1 Pasteur Street, Warsaw, 02-093 Poland; 3 Department of Anatomy and Cell Biology, Faculty of Medicine and Health Sciences, McGill University, Montréal, QC Canada; 4 Faculty of Chemistry, Biological and Chemical Research Centre, University of Warsaw, 101 Żwirki i Wigury Street, Warsaw, 02-089 Poland; 5 Łukasiewicz Research Network - PORT Polish Center for Technology Development, 147 Stablowicka Street, Wrocław, 54-066 Poland

**Keywords:** Radial spoke, Label-free, TMT, Mass spectrometry, Cryo-ET, Cilia, Adenylate kinases, Casein kinase, PKA

## Abstract

Radial spokes, RS1, RS2, and RS3, are T-shaped, multiprotein complexes that transmit regulatory signals from the central apparatus to outer doublet complexes, including dynein arms. Radial spokes, especially RS3, differ in their morphology, protein composition, and RS base-docked IDAs. Spokes’ defects alter cilia beating frequency, waveform, and amplitude, leading, in humans, to primary ciliary dyskinesia and male infertility. The role of the particular spokes remains unclear. Ciliate *Tetrahymena thermophila* has three RSP3 paralogs and two or three paralogs of some other RSPs. Using multiple complementary approaches, we demonstrated that *Tetrahymena* forms RS1 and RS2 subtypes, each with a core composed of various RSP3 paralogs and one type of RSP3-less RS3. We elucidated proteomes of RS1 and RS2 subtypes, RS3, and identified novel RS proteins, including enzymatic proteins involved in local regulation of ADP/ATP levels, the guanylate nucleotide pathway, and protein phosphorylation, whose presence further diversifies RSs’ properties and likely functions.

**Supplementary Information:**

The online version contains supplementary material available at 10.1007/s00018-025-05871-x.

## Introduction

Motile cilia and homologous flagella are highly evolutionarily conserved, hair-like cell protrusions supported by a microtubular skeleton, the axoneme, composed of nine peripheral doublets and two central singlets. The central microtubules, C1 and C2, together with attached complexes, the projections, form the central apparatus (CA), a structure believed to initiate signals required for planar cilia beating. It is proposed that transient interactions between CA projections and outer doublet-docked radial spokes (RSs) enable transmission of the regulatory signals to outer doublet complexes, including the nexin-dynein regulatory complex (N-DRC) and motor protein-containing outer and inner dynein arms (ODAs and IDAs) [1–5].

RSs are T-shaped, approximately 40-nm-long complexes with three morphologically distinct regions: (i) an elongated stalk docking the entire RS structure to the outer doublet and having some conformational flexibility enabling RS, especially RS1, to tilt [2, 6]; (ii) an orthogonal head transiently coming in contact with CA projections; and (iii) a neck connecting a stalk and a head. Within the 96-nm axonemal repeat unit, RSs are arranged as triplets (RS1, RS2, RS3). The distance between the neighboring spokes is specific to each spoke pair, enabling their identification [7, 8]. Ultrastructural studies of cilia and flagella from evolutionarily distant species demonstrate that RS1, RS2, and RS3 spokes, initially recognized as morphologically similar structures [7], differ in their architecture and protein composition. The most striking example of RSs’ diversity comes from *Chlamydomonas*, where RS1 and RS2 are full-length structures, while RS3 is reduced to a short knob [9–11]. Even in species forming all RSs as full-length complexes [12], the RS3 differs from RS1 and RS2. Moreover, RS1 and RS2 are also not identical [2, 13–21].

Besides differences between spokes within the 96-nm axonemal unit, the architecture of particular spokes varies in different species. In choanoflagellate *S. rosetta* [22] and metazoans (sea urchin [9, 23], *Danio rerio* [24], mice, and humans [21, 25]), the RS1 and RS2 heads are symmetrical, H-shaped structures composed of two parallel halves divided by a deep cleft. In contrast, in *Chlamydomonas*, *Tetrahymena*, and *Trypanosoma*, the head of RS1 and RS2 is nearly flat, with a less pronounced cleft but with large lobes extending its area [9–11, 17, 23, 26]. Similar, the morphology of the RS3 head differs between *Tetrahymena* and metazoans [23]. Furthermore, in *P. rosetta* and studied metazoans, the RS heads are either separated, or a thin connection can exist between RS2 and RS3 [21–25]. In contrast, in *Tetrahymena*, *Chlamydomonas*, and *Trypanosoma*, all RS heads are firmly connected [9–11, 17, 23]. Strikingly, in mice and humans, the RSs present in sperm flagella and cilia of multiciliated cells are also not identical. In sperm cell flagellum, RSs are accompanied by additional structures not observed in cilia of multiciliated cells: the barrel-shaped density positioned near RS1, identified as TRiC [18], the RS2-RS3 cross-linker, and a density near the RS3 base (RS3 scaffolds) [25]. Moreover, while multiciliated cells express Rsph1 and Rsph4a (the RS head subunits), in sperm flagellum, the RSs contain Rsph6a, a paralog of Rsp4a [27] and possibly Rsph10 [28–30]. The differences in the RSs’ morphology reflect some differences in the RSs’ protein composition and perhaps inter-spoke arrangement of some proteins, as was recently shown for *Chlamydomonas* [15], mammals [18, 31], and *Trypanosoma* [32].

In *Chlamydomonas*, a radial spoke protein 3 (RSP3) dimer, a core component of RSs [33], stretches from the RS base (N-termini) to the RS head (C-termini) [3] and directly interacts with most of the RSPs [15]. Pioneering studies using the *Chlamydomonas pf14* (paralyzed flagella) mutant carrying a mutation in the *RSP3* gene [34] and lacking full-length spokes, RS1 and RS2, led to the identification of RS subunits, RSP1-RSP23. In contrast to RS1 and RS2, the knob-like RS3 is maintained in the *Chlamydomonas pf14* mutant [9, 10] but missing in the *fap61* (CaM-IP3) and *fap91* (CaM-IP2) mutants [35]. Our studies showed that in *Tetrahymena*, knockout of *CFAP91* affects the RS3 and RS2 base [13] while deletion of *CFAP61* and *CFAP251* eliminates part of the RS3 stalk (less frequently, the entire RS3) or an arch-like structure at the RS3 base, respectively [19],. More recently, Lrrc23 was identified as a component of the RS3 head in mouse sperm flagellum [16] while this year, a protein composition and an atomic model of RS3 in bovine sperm [18] and mouse respiratory cilia [31] were proposed. Strikingly, in mice and humans, mutations in *CFAP61*, *CFAP91*, *CFAP251*, and *LRRC23* cause male infertility but not primary ciliary dyskinesia or hydrocephalus, suggesting more differences in the RS3 structure and/or function between sperm flagellum and cilia in multiciliated cells [36–40]. To date, CFAP61, CFAP251, LRRC23, CFAP91, and very recently, AK7 [31] are the only proteins whose presence in RS3 was confirmed by a genetic knockout and ultrastructural studies.

To identify and verify the presence of the remaining components of RS3, we took advantage of existing *Tetrahymena* knockout mutants with RS defects (CFAP61-KO [19], CFAP206-KO [41], and CFAP91-KO [13]), and newly engineered mutants lacking RSP3 paralogs, and compared wild-type (WT) and mutant ciliomes using label-free and tandem mass tag (TMT) quantitative mass spectrometry. To verify if identified candidate RSPs (proteins diminished in mutant ciliomes) are indeed present in RSs or their vicinity, we performed co-IP and BioID assays. These proteomic approaches combined with cryo-electron tomography (cryo-ET) studies of RS mutants and cross-links data [42] allowed us to assign with a high probability the substantial number of known RSPs and newly identified proteins to either RS1, RS2, or RS3. Importantly, among newly identified proteins, we found not only structural proteins but also proteins having predicted enzymatic folds and thus likely having an enzymatic activity (further shortly, enzymes), including serine-threonine kinases, adenylate kinases that locally control ADP/ATP levels, and two enzymes from the guanylate nucleotide pathway. We also observed a destabilization of some single-headed IDAs in the analyzed RS mutants and obtained evidence suggesting that RS likely interacts with specific central apparatus components.

Here, we propose a model of *Tetrahymena* RSs, showing both ubiquitous and RS type-specific RSPs, as well as newly identified structural and enzymatic proteins. We postulate that *Tetrahymena* has subtypes of RS1 and RS2 spokes that increase the heterogeneity of RSs and perhaps their function. Our data, besides expanding the general knowledge regarding RS composition, can contribute to a better understanding of the molecular mechanisms regulating cilia motion.

## Results

### Deletion of *Tetrahymena* Rsp3 paralogs differently affects cilia beating

In contrast to *Chlamydomonas* and animals, the *Tetrahymena* genome encodes three RSP3 orthologs, Rsp3A, Rsp3B, and Rsp3C (Supplementary Fig. 1). Out of these three paralogs, Rsp3B is the most similar to *Chlamydomonas* and metazoan RSP3 (39% identity and 63% similarity to human RSPH3 within the conserved region encompassing the axoneme targeting domain [43] and the radial spoke 3 domain [44]). Similar to RSP3 in other species, *Tetrahymena* Rsp3 paralogs have a predicted PKA-anchoring domain (AKAP) [45, 46] and amphipathic helix AH-D, binding proteins with a DPY-30 domain [46] (Supplementary Fig. 1). Three LC8-interacting TQT-like motifs, common in N-termini of *Chlamydomonas* and vertebrate RSP3 [47], are predicted only in Rsp3B, while Rsp3A and Rsp3C have one and two TQT-like motifs, respectively. Interestingly, the Rsp3C is unusual in having a predicted N-terminal ARF (ADP-ribosylation factor) domain and a C-terminal tail enriched in glutamic acid residues. We identified the Rsp3C-like RSP3 orthologs only in ciliates closely related to *Tetrahymena*, such as *Paramecium*, *Ichthyophthirius*, and *Pseudocohnilembus*, but not in *Stentor* or *Stylonychia* species.

Ciliary Rsp3A, Rsp3B, and Rsp3C have several isoforms, suggesting their posttranslational modifications, likely including the most common, phosphorylation (Supplementary Fig. 2). In human RSPH3, two threonine residues, T243 and T286, are phosphorylated by ERK1/2 [45]. In corresponding positions, the RSP3 orthologs have either threonine or serine residues (Supplementary Fig. 1), except for *Tetrahymena* Rsp3A, missing the first, and Rsp3B, missing a second putative phosphorylation site.

**Fig. 1:**
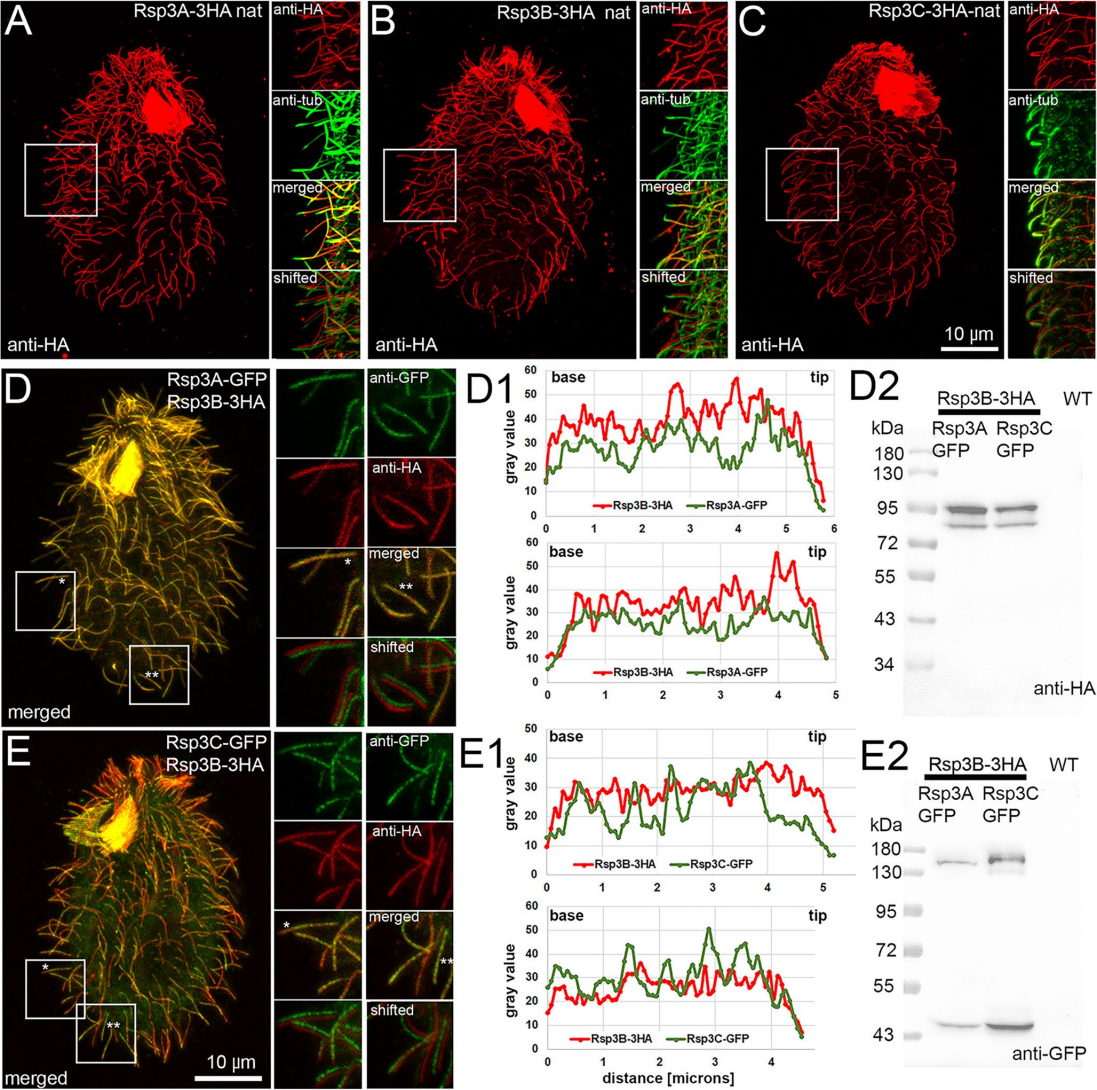
Ciliary localization of Rsp3 paralogs. (**A-C**) 3HA-tagged Rsp3 paralogs expressed under the control of the transcriptional promoters localize along the entire cilia length except for the ciliary tip. Cells were co-labeled with (**A-B**) anti-acetylated tubulin antibody or (**C**) polyG to visualize the ciliary shaft. To the right, are presented the magnified fragments of the cells as indicated by the white insets, showing cilia stained with anti-HA (red) and anti-tubulin (green) antibodies, and merged images (red and green). Below are images showing both channels but with some shifts to better visualize the presence of the Rsp3 paralogs along the entire cilia. (**D-E2**) Co-localization of Rsp3B-3HA and (**D**) GFP-tagged Rsp3A or (**E**) Rsp3C. To the right, the magnified fragments of the cells as in (**A-C**). (**D1-E1**) Plots representing the intensity of the 3HA (red) and GFP (green) fluorescence signal in exemplary cilia marked by stars in (**D** and **E**) and shown in enlarged insets. (**D2-E2**) Western blot analyses of co-expressed Rsp3 paralogs

**Fig. 2:**
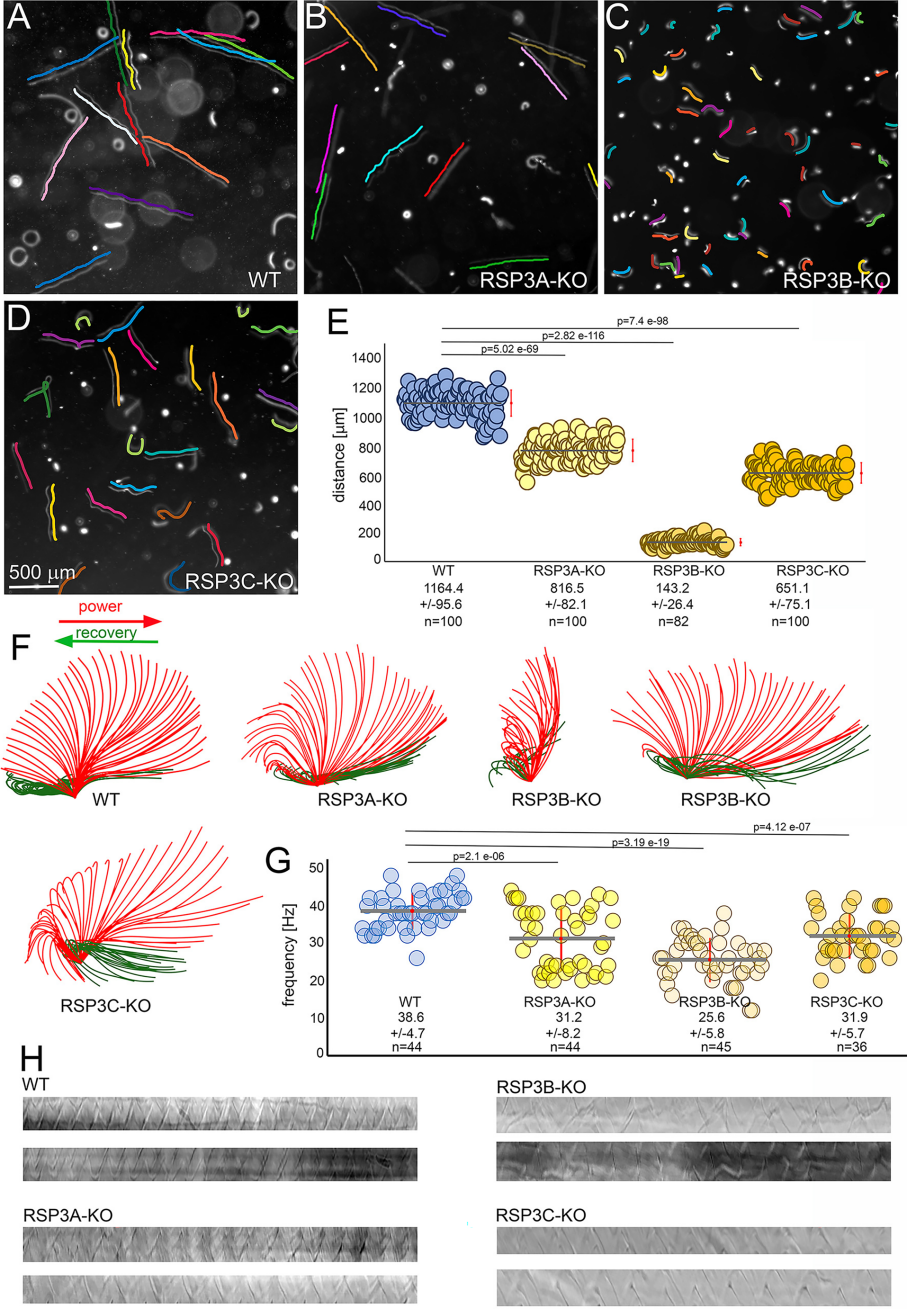
Deletion of each *RSP3* gene differently affects cilia beating. (**A-D**) WT (**A**), RSP3A-KO (**B**), RSP3B-KO (**C**), and RSP3C-KO (**D**) trajectories were recorded for 3 s using a high-speed video camera. The cell swimming paths are indicated by the parallel colored lines. Bar = 500 μm. (**E**) A comparison of the length of trajectories recorded for 3 s. The red bar position to the right represents the standard deviation. The WT cells swam on average 388+/−32 μm/s (*n* = 100), and the swimming speeds of the RSP3A-KO, RSP3B-KO, and RSP3C-KO mutants were reduced to 272+/−27 μm/s (*n* = 100), 48+/−9 μm/s (*n* = 82), and 217+/−25 μm/s (*n* = 101), respectively. Statistical significance was calculated using a student t-test. (**F**) Drawings showing examples of the most frequently observed subsequent positions of a cilium of WT, *RSP3A*, and *RSP3C* knockout cells and the most extreme difference in cilia beating in RSP3B-KO cells. The ciliary waveform and amplitude in the RSP3B-KO mutant can vary both in the case of neighboring cilia and during subsequent cycles of the same cilium. (**G**) Graph showing a range of cilia beating frequencies in WT and *RSP3* knockout mutants. The red bar represents the standard deviation. Statistical significance was calculated using a student t-test. (**H**) Examples of the kymographs used to calculate cilia beating frequency

*Tetrahymena* Rsp3 paralogs expressed as fusions with a C-terminal 3xHA tag under the control of a transcriptional promoter localized along the entire cilia length except the ciliary tip (Fig. 1A-C). When co-expressed, 3xHA- and GFP-tagged Rsp3 paralogs were incorporated concurrently into growing cilia (Supplementary Fig. 3), although, we observed some minor fluctuation of the Rsp3C-GFP signal along the cilium length in both elongating and full-length cilia (Fig. 1D-E). To explore the significance of Rsp3 paralogs, we constructed knockout strains (Supplementary Fig. 4). Deletion of a single *RSP3* gene affected cells swimming and cilia-beating parameters (Fig. 2), and in the case of RSP3B-KO, reduced cilia length to ~ 84% of the WT cilia (Supplementary Fig. 5A). The loss of Rsp3B had the most profound effect on cell motility and reduced the swimming speed to ~ 12% of the WT cell speed, while deletion of either *RSP3A* or *RSP3C* was less damaging (70% and 56% of the WT cell speed, respectively). *Tetrahymena* cells are propelled by the forces generated by cilia beating in metachronal waves. The cilium beating cycle has two phases, the power and recovery strokes that occur in different planes in relation to the cell surface, perpendicular and parallel, respectively. During the power stroke, the cilium is basically straight, while during the recovery stroke, it bends, and the bend position shifts from the cilium base to the tip as the recovery stroke progresses (Fig. 2F, Supplementary Video S1) [13, 48]. Deletion of *RSP3A* or *RSP3C* did not change the ciliary waveform and beat amplitude but slightly affected cilia metachrony and beating frequency (Fig. 2F-H, Supplementary Fig. 5B-E, Supplementary Videos 2 and 4). In contrast, cilia lacking Rsp3B were beating in a less coordinated manner, with significantly lower frequency and inconsistencies in the waveform and amplitude between neighboring cilia and between subsequent beating cycles performed by the same cilium (exhibiting periods of nearly normal and highly modified patterns). Moreover, the distal end of the majority of RSP3B-KO cilia was still bending during the power stroke (Fig. 2F, Supplementary Fig. 5B-E, and Supplementary Video S3). A delayed straightening of the distal cilium end was also observed in some cells lacking Rsp3C.

### RSP3 paralogs and heterogeneity of RS1 and RS2 spokes

**Fig. 3:**
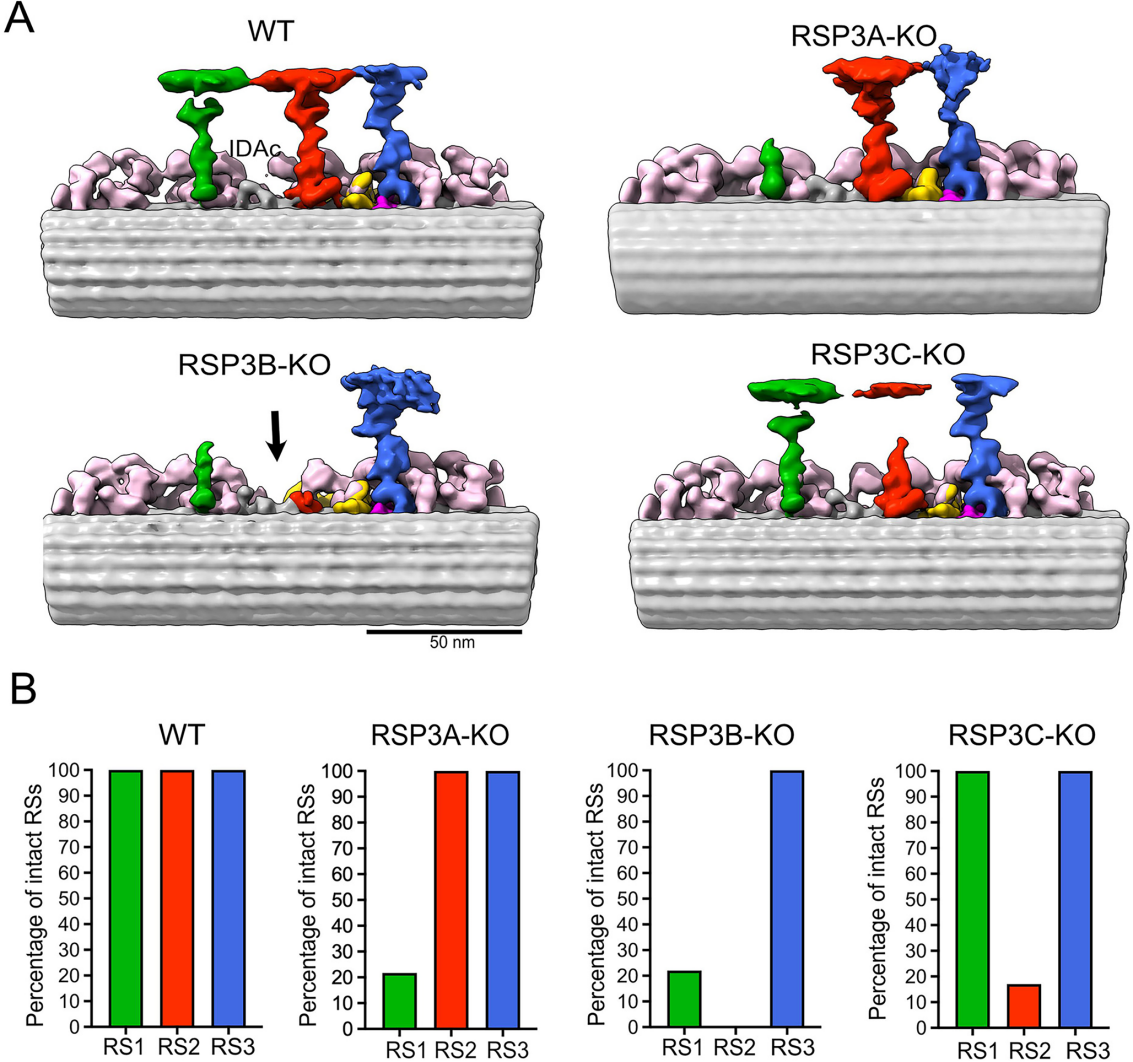
Cryo-ET and subtomogram averaging analyses of the ciliary ultrastructure of WT cells and *RSP3* knockout mutants. (**A**) Subtomogram averages of WT, RSP3A-KO, RSP3B-KO, and RSP3C-KO mutants. RS1: green; RS2: red; RS3: blue. IDAs: light pink, N-DRC: yellow, Ccdc96-Ccdc113 complex: dark pink. The black arrow in the RSP3B-KO subtomogram points to the place corresponding to the position of IDAc in the WT cilia. (**B**) Quantification of intact RSs in WT and RSP3 mutant cells. Each graph shows the percentage of intact RSs. The analysis was performed using subtomogram classification, revealing structural differences in RS integrity

The differences in the phenotype of *RSP3* knockouts suggest that elimination of particular paralogs affects spokes of different types and/or different numbers of spokes. Cryo-ET and subtomogram averaging of the RSP3-KO mutants revealed paralog-specific ultrastructural defects (Fig. 3, Supplementary Table 1). Averages of all 96-nm subunits showed that RSP3A-KO and RSP3C-KO mutant cilia lacked RS1 and RS2 spokes, respectively, except for a spoke base, while RSP3B-KO lacked the entire RS2 and the RS1 head (Fig. 3A). Of note, in all *RSP3* mutants, the RS3 was intact, strongly suggesting that none of the Rsp3 paralogs is an RS3 component. The densities remaining in analyzed mutants, especially at the RS1 and RS2 bases, were reduced compared to that in WT cilia, suggesting RS heterogeneity. Therefore, we performed classification of the base and head images (Fig. 3B, Supplementary Figs. 6 and 7). Our classification revealed that, in the RSP3A-KO mutant, the RS1 was intact in approximately 22% of the 96-nm units. In the RSP3C-KO mutant, 19% of 96-nm repeating units lacked RS2, while 17% retained the entire RS2, and 58% contained a long stump of RS2. In RSP3B-KO, all 96-nm repeats lacked RS2, while RS1 was intact in only 22% of analyzed units.

**Fig. 4:**
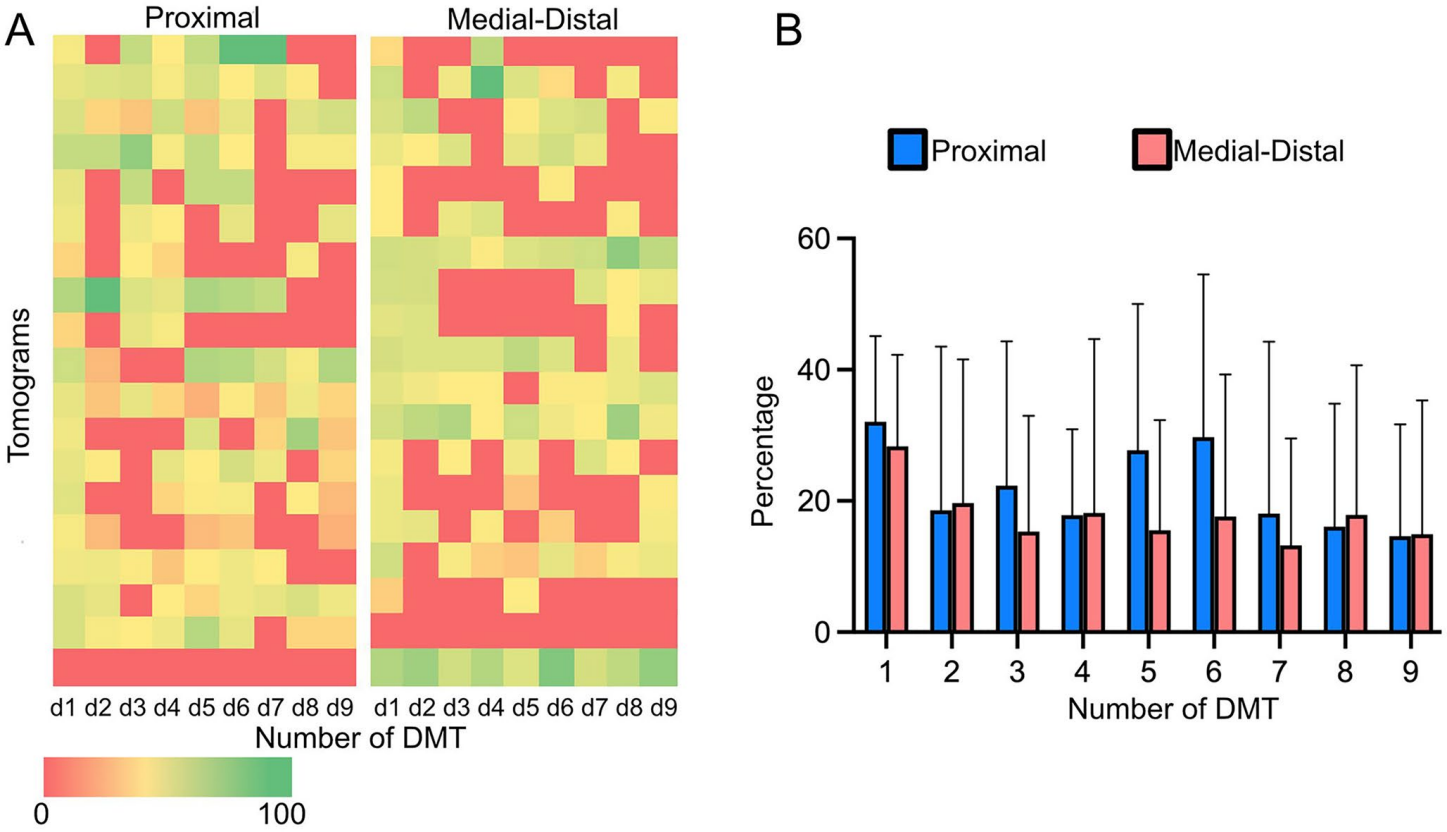
Ultrastructural analyses of Rsp3C-containing RS. (**A-B**) Distribution of intact RS2 in the RSP3C-KO axoneme. (**A**) Heat map showing the distribution of particles within each doublet at the proximal and medial-distal ends of the axoneme. The doublet (DMT) with the highest particle count is considered the first doublet in the heat map. To assess the distribution of subtomograms with intact RS2 across the doublets, counts were normalized; “0” indicates that none of the subtomograms have intact RS2, while “100” that particular doublet has all RS2 intact. (**B**) Graph representing the mean percentage of intact RS2 subtomograms for a specific doublet. Tomograms were treated as biological replicates, thereby collecting biological variability within each doublet. Error bars (standard deviation) indicate the variability in the data across tomograms

We concluded that Rsp3A is a component of RS1 spokes, while Rsp3C is a component of RS2 spokes. In contrast, Rsp3B is likely a main core protein in RS1 and RS2 spokes. Lack of Rsp3C either partly affected RS2 spokes or, less frequently, led to their complete elimination (Supplementary Fig. 6). To evaluate the distribution of the intact RS2 along the doublet microtubule in RSP3C-KO, we performed a statistical analysis of subtomograms. We found that there was no significant difference in the distribution of particles between the proximal and medial-distal regions of each doublet within the axoneme (Fig. 4).

To further study the extent of RS defects, we isolated cilia from WT and *RSP3-KO* cells and performed comparative proteomic mass spectrometry analyses using label-free (LFQ) and TMT 10-plex isobaric mass tagging approaches. The experiments were repeated three times to generate independent sets of data. At least 75% of proteins were present in all three replicates. Approximately one thousand ciliary proteins from WT cells and *RSP* mutants were identified in LFQ experiments, while nearly four times more were detected using the TMT approach. Based on principal component analysis of obtained ciliomes using Ingenuity Pathway Analysis, the RSP3A-KO ciliome is most similar to that of WT (Supplementary Fig. 8). Changes in the protein levels were analyzed using Perseus software (v. 2.0.3) [49]. The t-test (*p* < 0.05) was used to identify proteins whose level significantly changed (a ± 1.5-fold) in mutants’ versus that in WT cilia. The obtained data are graphically represented as Volcano plots -log (*P* value) vs. log_2_ (fold change of mutant/WT, Supplementary Fig. 8) and Venn diagrams (Supplementary Fig. 9A-C). If there were discrepancies between LFQ and TMT data, we interpreted changes in the protein level according to the latter analyses.

Global mass spectrometry analyses of the RSP3A-KO, RSP3B-KO, and RSP3C-KO mutant cilia confirmed that the protein encoded by the targeted gene was completely eliminated in the respective mutants (Supplementary Table 2). The knockout of *RSP3A* or *RSP3C* did not significantly affect the levels of other Rsp3 paralogs, while in RSP3B-KO cilia, the level of Rsp3A was diminished, suggesting that Rsp3A-Rsp3B could form a heterodimer or that in the absence of RS2, some Rsp3A-containing RS1 spokes are unstable. Of note, in none of the *RSP3* mutants was the level of remaining Rsp3 paralogs significantly elevated, suggesting that if the substitution of a missing paralog by another one occurs, it is a minor phenomenon.

**Fig. 5:**
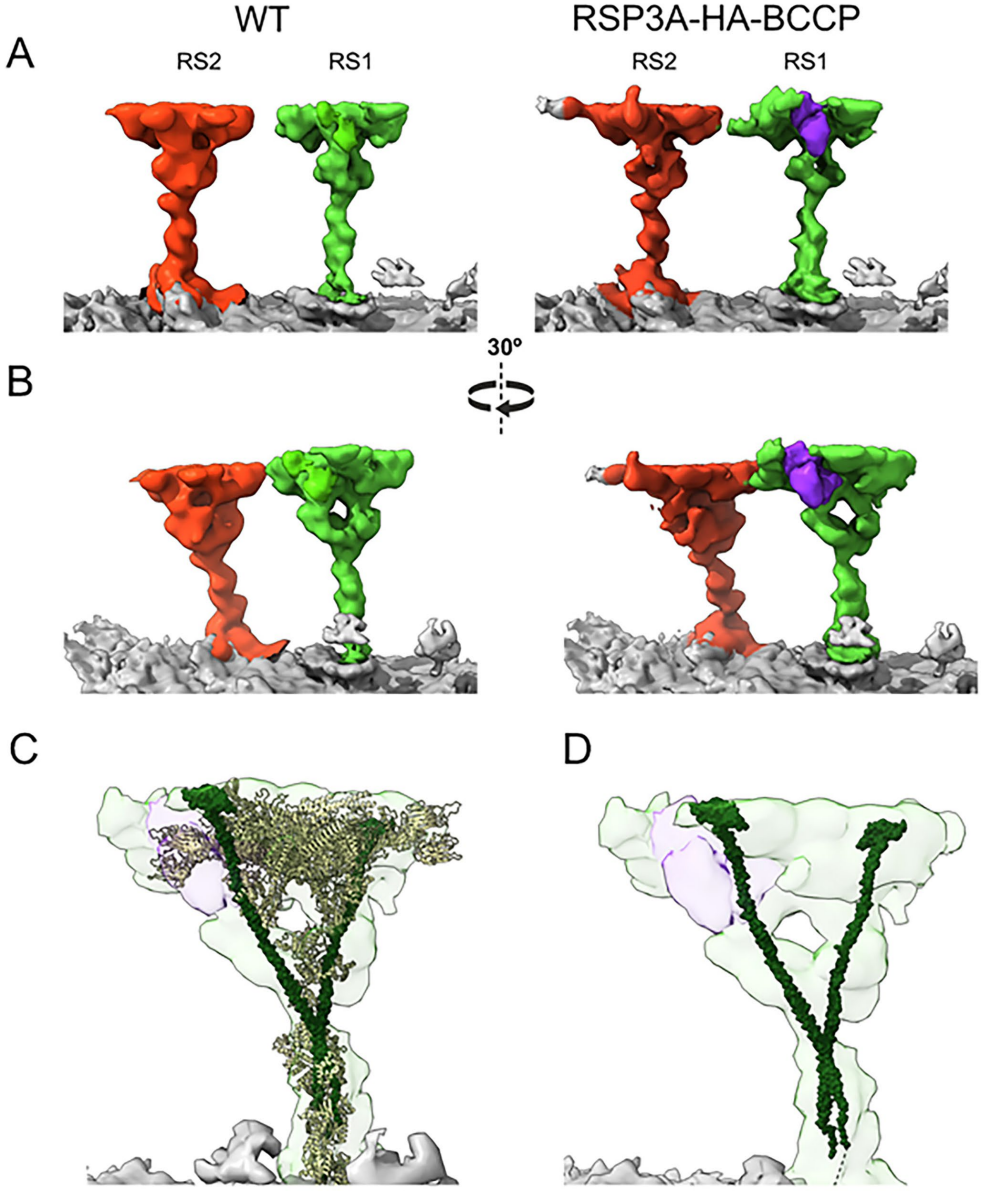
Subtomogram structure of the outer doublet in WT and Rsp3A-HA-BCCP expressing cells. (**A**) Locally refined structures of RS1 and RS2 in the WT and BCCP-tagged axoneme. RS1 is shown in green, RS2 in red, and streptavidin in purple. (**B**) View of the doublet with a 40-degree rotation. (**C**) The model of RS1 from *Chlamydomonas reinhardtii*, fitted into the RS1 density from the Rsp3A-HA-BCCP map. (**D**) The complete RS1 model from *Chlamydomonas reinhardtii*, with all proteins hidden except RSP3 proteins

The RSP3 is present in RS as a dimer [33]. Based on cryo-ET and proteomic data, we propose that *Tetrahymena* cilia could have up to three RS1 subtypes with a core composed of (i) Rsp3A dimer (RS1 missing in RSP3A-KO but intact in RSP3B-KO cilia), (ii) Rsp3B dimer (RS1 missing in RSP3B-KO but present in RSP3A-KO cilia), and (iii) Rsp3A-Rsp3B heterodimer (RS1 missing in both *RSP3A* and *RSP3B* mutants). The existence of the third class is supported by proteomic data showing that the level of Rsp3A was substantially reduced in *RSP3B* mutant cilia. On the other hand, in *Tetrahymena*, the RSs’ heads are connected, and therefore the RSs stabilize one another. Thus, some Rsp3A-containing RS1 spokes could be destabilized (a secondary effect) in the RSP3B-KO mutant, leading to a moderate reduction of the Rsp3A level. To solve this issue, we searched the *Tetrahymena* cilia interactome [42] and found a highly confident cross-link between Rsp3A and Rsp3B (RSP3A K172 to RSP3B K195), suggesting a direct contact between these proteins. We also engineered cells expressing Rsp3A-BCCP under the control of the *RSP3A* promoter and analyzed BCCP localization using cryo-ET and subtomogram averaging. A comparison of the subtomogram-averaged maps from the WT and Rsp3A-BCCP cells revealed an additional density on the IDA-facing side of the RS1 head (Fig. 5A-B). Moreover, when the entire RS1 model from *Chlamydomonas* was fitted into our maps (Fig. 5C), the extra globular density corresponding to streptavidin was found near the C-terminus of only one copy of the *Chlamydomonas’s* RSP3 (Fig. 5D). Taken together, the above data suggest that some of the RS1 spokes could indeed have a core composed of Rsp3A-Rsp3B heterodimers, and thus it is possible that in *Tetrahymena* there are three types of RS1, depending on the Rsp3 dimer composition.

The absence of Rsp3C affected RS2, but the entire RS2 was missing in only ~ 19% of the analyzed subtomograms, which is in striking contrast with RSP3B knockout, in which all RS2 were eliminated. Thus, the presence of RS2 spokes containing Rsp3C homodimer is unlikely or very infrequent. We propose that Rsp3B is a component of all *Tetrahymena* RS2 spokes and that RS2 affected in the RSP3C-KO mutant could contain either an Rsp3B-Rsp3C heterodimer or an Rsp3B homodimer additionally stabilized by Rsp3C. The unaltered level of Rsp3B in *RSP3C* knockout suggests that in the absence of Rsp3C, the Rsp3B-containing, partly collapsed RS2 remnants were still attached to the axoneme but no longer maintained their T-shaped structure (Supplementary Fig. 6, RS2 head masked). Thus, likely, Rsp3C is present in a subset of RS2 spokes. Surprisingly, the level of Rsp3C in RSP3B-KO cilia was also unaltered, suggesting that perhaps Rsp3C docks to the axoneme independently of Rsp3B. We did not find cross-link data supporting the interactions between Rsp3B and Rsp3C; however, this does not exclude the possibility that Rsp3B and Rsp3C can co-assemble, forming the RS2 spoke. To summarize, in contrast to RSs from other species, in *Tetrahymena* there are subtypes of RS1 and RS2 spokes, increasing spoke heterogeneity. Based on the available data, the significance of this phenomenon remains unknown.

### Identification of *Tetrahymena* RSP orthologs and protein composition of RSs

The bioinformatics search of *Tetrahymena* RSP orthologs (Supplementary Fig. 10, Supplementary Table 3) revealed that besides three Rsp3 paralogs, *Tetrahymena* also has three paralogs of Rsp4/6 and two of Rsp7, Rsp12, Rsp16, and Cfap198, while other RS proteins are encoded by a single gene. Most of the *Tetrahymena* RS proteins are well evolutionarily conserved (Supplementary Table 3), and their identification was straightforward, but finding true orthologs of Rsp1, 5, 7, 10, and 12 required additional information. Therefore, to identify these less-conserved RSPs as well as new RS subunits, and to assign with a high probability the identified proteins to particular spokes, we performed additional experiments. We engineered *Tetrahymena* cells expressing RS proteins in fusion with either C-terminal 3HA or HA-BirA* tags under the control of the transcriptional promoter and performed co-IP or BioID assays, respectively, followed by mass spectrometry analyses to identify ciliary proteins that either directly or indirectly interact with 3HA-tagged Rsp3A, Rsp4A, Rsp4B, or Rsp4C (Supplementary Table 4) or are in the vicinity of Rsp3 or Rsp4 paralogs, Cfap61, Cfap91, or Cfap206 (Supplementary Fig. 11, Supplementary Table 5). Such analyses enabled identification of these less conserved RS proteins (Supplementary Table 6). Furthermore, we compared ciliomes of WT and previously described *Tetrahymena* RS mutants (Supplementary Fig. 9D-E): (i) CFAP206-KO, missing either the entire RS2 or only its base, rarely accompanied by minor RS3 defects [41]; (ii) CFAP61-KO, lacking a part of the RS3 stalk or the entire RS3 [19]; and (iii) CFAP91-KO, missing RS3 and a part of the RS2 base [13]. Proteins whose level was reduced in CFAP91-KO cilia, were assigned as RS3 subunits if their level was unchanged in RS2 mutants (RSP3B-KO, RSP3C-KO, CFAP206-KO) or as RS2 components if they were also diminished in RS2 mutants.

**Fig. 6:**
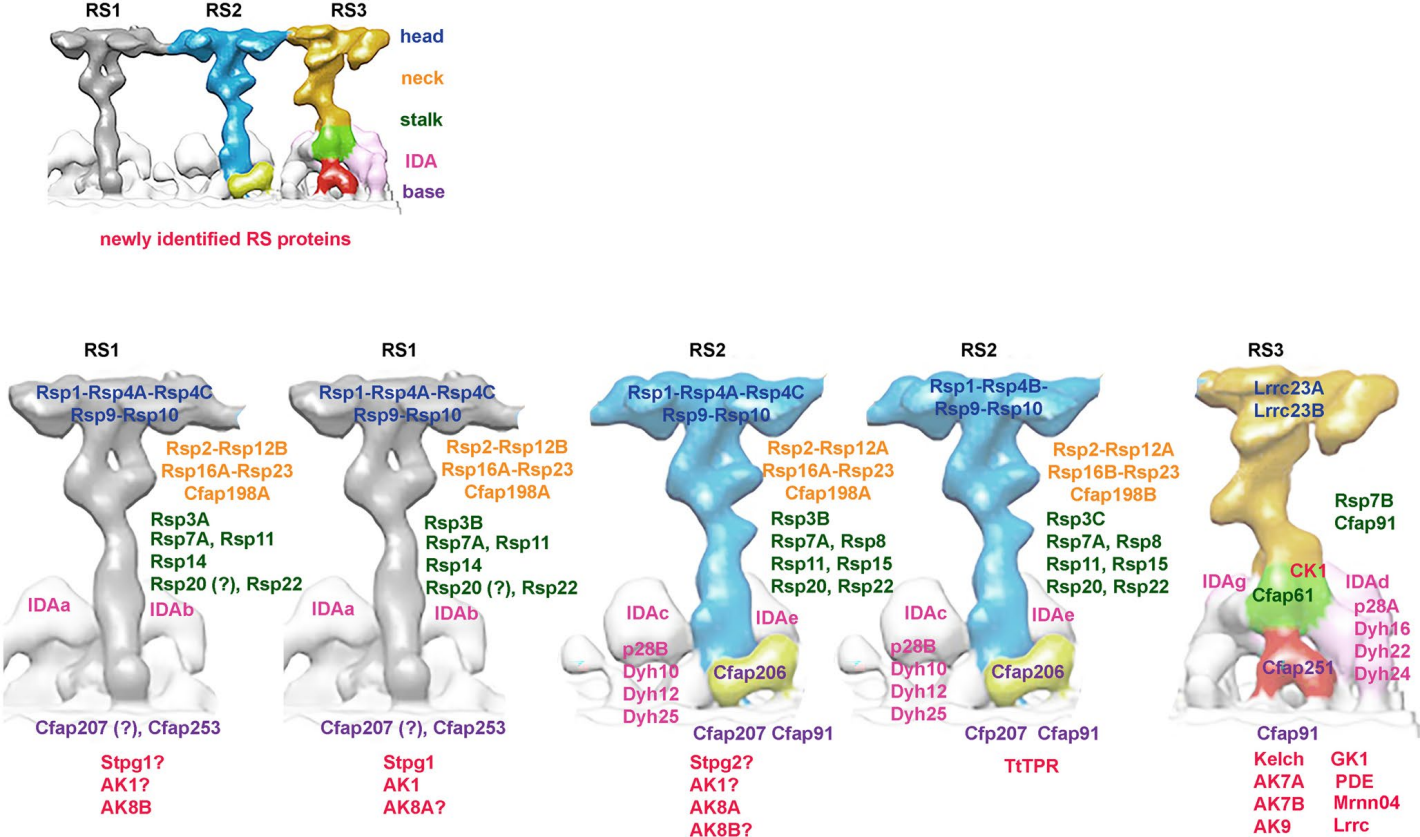
A schematic summary of the RS protein composition in *Tetrahymena* cilia prepared based on collected ultrastructural and proteomic data. A fragment of Fig. 3B published in our earlier paper [19] was used as the RSs template: RS1 (grey), RS2 (blue), and RS3 (yellowish with the positions of Cfap61 and Cfap251 marked in green and red, respectively). The upper panel shows RSs with marked levels of the RS head, neck, stalk, base, docked single-headed IDAs, and newly identified RS proteins. Please note that the same color code was used in the lower panel to describe the protein composition of the indicated RS regions: Rsps building the RS head in navy blue, neck in orange, stalk in green, RS base in violet, new Rsps in red, and IDAs names and components in pink

Because of some specific features of *Tetrahymena* RS, dissecting the RS protein composition is challenging. In contrast to *RSP3B* knockout, deletion of *RSP3A* or *RSP3C* affects only a fraction of spokes, and thus, although the level of RS proteins decreases, the change could be statistically insignificant (Supplementary Tables 7–9). Furthermore, different RSP paralogs can be present in different RS subtypes. Finally, because in *Tetrahymena* RSs’ heads are connected, proteins present in the neighboring spokes are likely to be detected in co-IP and BioID assays. Supplementary Table 10 summarizes reasons for assigning known RS components and newly identified proteins to particular spokes (Fig. 6).

#### Components of the radial spoke head

*Tetrahymena* has three paralogs of the RS head protein, RSP4/6: Rsp4A, Rsp4B, and Rsp4C, and a single ortholog of the remaining RS head components, Rsp1, Rsp2, Rsp9, and Rsp10. In contrast to *Chlamydomonas*, but similar to mammals, *Tetrahymena* spokes lack RSP5. The levels of all head proteins, except for Rsp4B, were significantly diminished in the RSP3B-KO mutant, lacking all RS2 and two-thirds of RS1 spokes. The same proteins were diminished in RSP3A-KO cilia, but estimated changes were statistically insignificant, likely because only a fraction of RS1 was missing in this mutant. Strikingly, the Rsp4B was almost completely eliminated in RSP3C-KO cilia. We propose that heads in all RS1 subtypes and in RS2 missing Rsp3C are composed of the same subunits, while in Rsp3C-containing RS2 spokes, Rsp4B is a dominating Rsp4 paralog (Fig. 6).

The elimination of leucine-rich repeat-containing 23 (LRRC23) impairs the assembly of the RS3 head in human and mouse sperm axoneme, indicating that LRRC23 is likely an RS3 head component [16]. In *Tetrahymena*, the orthologous Lrrc23A and Lrrc23B were unaltered in mutants affecting only RS1 and/or RS2 spokes but reduced in the *CFAP91* mutant [13]. Both Lrrc23 proteins have a C-terminus enriched in glutamic acid residues (especially Lrrc23B), which may increase the negative charge of the RS3 head surface. We propose that in *Tetrahymena*, both Lrrc23A and Lrrc23B are RS3 head components.

Among proteins biotinylated in cells expressing BirA*-tagged RS head subunits, we also identified components of the central apparatus (Supplementary Table 11), mainly subunits of the C1b, C1d, and C2b projections. Thus, either C1b, C1d, and C2b primarily interact with the RS head, or a contact between the RS head and these projections is longer, enabling more efficient biotinylation. Furthermore, C1d components, Cfap46 and Cfap54, were preferentially biotinylated when RS1-RS2 head subunits were expressed as BirA* fusions, while in cells expressing Lrrc23-BirA*, besides C1d, also C2b and C1b proteins were biotinylated, suggesting preferences in RS-CA projection interactions.

#### Components of the radial spoke neck

*Tetrahymena* has a single Rsp2, likely a single ortholog of Rsp23, and paralogous Rsp12A and Rsp12B, Rsp16A and Rsp16B, and Cfap198A and Cfap198B. The 70 amino acid long FAP385 modeled in *Chlamydomonas* RS neck protein [15] is strikingly similar to the very N-terminal fragment of adenylate kinase 8A (Ak8A). *Tetrahymena* and *Chlamydomonas* RSP2 and RSP23 orthologs share similarity only within their N-termini containing predicted DPY-30 [50] and NDK (nucleoside diphosphate kinase) [51] domains, respectively. In *Tetrahymena RSP2* knockdown cilia (Supplementary Table 2), only Rsp12A and Rsp12B were significantly diminished, suggesting that Rsp12 likely directly binds to Rsp2. The genome of *Tetrahymena* encodes two proteins similar to Rsp23, Cfap67A (TTHERM_000372529) and Cfap67B (TTHERM_00266490). Although neither of them was diminished in analyzed mutants, Cfap67A but not Cfap67B was identified in co-IP and BioID assays, suggesting that Cfap67A is the main, if not the only, Rsp23. Interestingly, recent single-particle cryo-electron microscopy and molecular modeling studies showed that Cfap67A is a microtubule inner protein (MIP) present in the A-tubule lumen [52]. Based on previous and here presented data, we propose that out of two *Tetrahymena* Cfap67 orthologs, Cfap67A likely dominates and has a double role in cilia - as an RSP and as a MIP. To summarize, the neck region of all types of RS1 is composed of Rsp2, 12B, 16A, 23/Cfap67A, and Cfap198A (Fig. 6). The RS2 lacking Rsp3C likely has a neck composed of the same proteins except that Rsp12B can be replaced by Rsp12A, while the neck of RS2-containing Rsp3C is built of Rsp2, 12A, 16B, 23/Cfap67A, and Cfap198B (Fig. 6).

#### Components of the radial spoke stalk and base

Rsp3 paralogs form a core of RS1 and RS2 stalks. The moderately diminished levels of Rsp3B and Rsp3C proteins in the *CFAP91* mutant (Supplementary Tables 2 and 7) can be due to defects in some RS2 [13, 41]. Similar to *Chlamydomonas*, *Tetrahymena* has two ARM-like (armadillo-like) motif-containing RS proteins (Rsp8 and Rsp14) and an LRR-containing Rsp15. Rsp14 is a subunit of the RS1, while Rsp8 and Rsp15 are RS2 components, independent of the Rsp3 paralog forming the RS stalk. *Chlamydomonas* RSP7 and RSP11 form a heterodimer positioned near RSP8 or RSP14 [15, 53]. Out of two RSP7 orthologs, *Tetrahymena* Rsp7A is a component of RS2 and likely of RS1, while Rsp7B is an RS3 subunit. *Tetrahymena* Rsp11, a small (7.9 kDa), basic protein (pI = 9) with a predicted RIIa domain and a limited similarity to *Chlamydomonas* RSP11 [53], similar to Rsp7A, is a component of both RS1 and RS2 (Fig. 6).

*Chlamydomonas* RSP20/CaM and RSP22/LC8 (cytoplasmic dynein light chain type 2) [54, 55] form a base of RS1 and RS2. CaM binds to FAP253 between RS1 and RS1-docked IDAs, while RSP22/LC8 forms a multimer, likely an RS docking site, composed of four homodimers in RS1 and two homodimers in RS2 [15]. Our proteomic data suggest that in *Tetrahymena* Rsp20/CaM and Rsp22/Lc8 associate with RS2 and perhaps RSP3B-containing RS1. On the other hand, the cryo-ET data showed that the RS1 base is missing only in a minor fraction of 96-nm subunits in the RSP3A-KO mutant (Fig. 3), which coincides with the unaltered levels of RS1 base proteins, Rsp20/CaM1, Rsp22/Lc8, Cfap207, and Cfap253 (Supplementary Tables 2 and 7), and together suggests their presence at the base of Rsp3A-containing RS1.

*Chlamydomonas* FAP207, a MORN motif-containing protein, was modeled as a part of the base of both RS1 and RS2, while the IQ motif-containing FAP253, orthologous to *Ciona* CMUB116 [12] and murine Iqub [20] was shown to be an RS1 adaptor [15, 20]. *Tetrahymena* and *Chlamydomonas* FAP253 are smaller than mammalian orthologs and lack the ubiquitin domain predicted in IQUB. In all tested *Tetrahymena* mutants, the level of Cfap253 was unaltered, but the protein was biotinylated in cells expressing BirA*-tagged Rsp3A or Rsp3B, together indicating that Cfap253 is an RS1 adaptor. Similar to Cfap253 and Lc8, the level of Cfap207 was unaltered in *RSP3A* and *RSP3C* knockouts but diminished in cells either lacking RS2 (RSP3B-KO) or having defects in the RS2 base (CFAP206-KO, CFAP91-KO), suggesting that the presence of Cfap207 at the RS2 base is Rsp3C-independent (Fig. 6).

The RS3 structure was unaltered in all RSP3-KO mutants, indicating that RS3 is an Rsp3-less spoke in *Tetrahymena.* Our previous analyses led to the identification of Cfap61 and Cfap251 as RS3 stalk and base components [19] and Cfap91 as a protein playing a role in RS2 base and RS3/RS3 base stability [13]. Here we showed that the RS3 stalk also contains Rsp7B (Fig. 6).

### New radial spoke candidate proteins

We identified several additional proteins whose level was diminished in analyzed mutants, suggesting that those are either new RS subunits or proteins positioned in the RS base vicinity (Supplementary Fig. 12, Supplementary Tables 2, 8, and 9). Based on the identified domains, we divided those proteins into structural proteins and proteins with putative enzymatic activity (shortly, enzymes).

The tetratricopeptide repeat-containing (TTHERM_00623040, TtTpr) and Kelch repeat-containing (TTHERM_00760390, TtKelch) proteins (Supplementary Tables 4, 5, 8, and 10) lack vertebrate or *Chlamydomonas* orthologs. They are diminished in *RSP3C* and *CFAP91* knockouts (TtTpr) or only in CFAP91-KO (TtKelch) and thus can be the components of Rsp3C-containing RS2 and RS3, respectively. The level of Lrrc (TTHERM_00046820) protein is reduced in both CFAP91 and CFAP61 mutants, strongly suggesting that this protein is a new RS3 subunit. The Mrnn04 (TTHERM_00324550), a MORN domain-containing protein orthologous to mammalian MORN5, is most probably also RS3 component, as recently, based on AI modeling, MORN5 was proposed to be the subunit of the RS3 stalk [18, 31]. Out of two other identified proteins, STPG1 and STPG2 (sperm-tail PG-rich repeat), the latter, which is similar to Stpg2 in mice and *Chlamydomonas* (CHLRE_09g415650v5), was reduced in RS1 or RS2 mutants. We propose that Stpg proteins are not RS components but are rather positioned near the RS base.

Cilia beating depends upon a constant supply of ATP and its uniform distribution within the cilium. Ciliary ATP is either delivered from the cell body by diffusion or produced within cilia [56]. Adenylate kinases (Ak) reversibly catalyze the ATP + AMP ↔ 2 ADP reaction and thus locally control the ATP level and maintain the homeostasis of adenine nucleotides [57]. Six Aks (types 1, 7, 8, and 9) identified in co-IP and BioID assays were diminished in different RS mutants (Supplementary Table 9). Based on these data, we propose that Ak8B is a component of Rsp3A-containing RS1, Ak8A (whose very N-termini is similar to FAP385) is a component of the Rsp3B-containing spokes, while Ak7A, Ak7B, and Ak9, which were reduced in CFAP91-KO cilia but unaltered in *RSP3* mutants, are components of RS3. Ak1 level was reduced in both *RSP3B* and *CFAP91* knockouts. Neither of the identified adenylate kinases was diminished in cells with knocked-out *RSP3C*. Interestingly, the Ak8B in the *RSP3A* mutant as well as Ak7B and Ak9 in the *CFAP91* knockout were nearly completely eliminated, strongly suggesting that Rsp3A-containing RS1 and RS3 are the main, if not the only structures where these enzymes localize. Interestingly, guanylate kinase, Gk1, and phosphodiesterase, Pde, are also RS3-associated enzymes, and cGMP could play a role in the signal transduction via RS3 or in the RS3 vicinity.

The RSP3 has an AKAP domain [58] and thus locally regulates PKA localization. PKA functions as a tetramer composed of two regulatory and two catalytic subunits [59]. We identified two PKA catalytic subunits and one regulatory subunit whose levels were altered in *RSP3B* and *RSP3C* mutants but not in *RSP3A* knockout cilia (Supplementary Table 9). The levels of two PKA subunits were also reduced in CFAP91-KO cilia, suggesting that PKA could also dock to RS3. Indeed, the RS3 subunit, Rsp7B, has an AKAP domain in its N-terminal fragment, suggesting that this protein could bind PKA. Finally, we also found that one of the *Tetrahymena* casein kinase 1 (Ck1)-type enzymes was eliminated in CFAP91-KO cilia (Supplementary Table 2), suggesting its specific association with the RS3.

### RS defect-related instability of inner dynein arms

Single-headed IDAs are docked in pairs at the RSs’: dynein a and b at RS1, c and e at RS2, and d and g at RS3. In *Chlamydomonas*, single-headed IDAs are composed of IDA type-specific dynein heavy chain (Dyh), actin, and either centrin (dyneins b, e, g) or light chain protein, p28 (dyneins a, c, d) [60]. The protein composition of single-headed IDAs in *Tetrahymena* is not fully resolved [61]. We found that RS defects affected some IDAs (Supplementary Table 12). *Tetrahymena* has three orthologs of p28: p28A, p28B, and p28C [61]. The level of p28B diminished in *RSP3B* and *CFAP206* knockouts, indicating (together with cryo-ET data) that p28B is a component of dynein c. In CFAP91-KO, the levels of both p28A and p28B were reduced [13], suggesting that p28A or both, p28A and p28B, are the components of dynein d.

The levels of Dyh10, 12, and 25 were diminished in the *RSP3B*, *CFAP206*, and *CFAP91* knockouts (in CFAP91-KO cilia lack Cfap206), which coincides with a lack of dynein c density (Fig. 3) [41]. In contrast, dynein e was unaffected in those mutants, suggesting that dynein c and dynein e are docked differently. In CFAP91-KO cilia, besides Dyh10, 12, and 25, the levels of Dyh16, 22, and 24 were also reduced [13], suggesting that these dynein heavy chains are components of IDAd or IDAg.

## Discussion

Identification of RSs’ components and RS-docking proteins is crucial for understanding the mechanism(s) enabling transduction of signals from the central apparatus to dynein arms. Despite the extensive research conducted using diverse ciliated species, including *Chlamydomonas* [15, 54, 55], *Tetrahymena* [13, 19, 41], *Ciona intestinalis* [62–64], and mice [2, 14, 16, 20, 40, 65], the model showing organization of RS3 subunits was only recently proposed [18, 31]. Here, using bioinformatics, genetic, proteomic, and cryo-ET approaches, we analyzed protein composition of RSs in a ciliate *Tetrahymena*, showing spokes’ heterogeneity and complexity. Strikingly, we not only found the differences between RS1, RS2, and RS3 spokes but also identified the subtypes of RS1 and RS2, generated primarily by the presence of different Rsp3 paralogs. The advantage of having Rsp3 paralogs, which, based on our data, do not substitute one another, is not clear. The presence of three (Rsp4) or two (Rsp7, Rsp12, Rsp16, and Cfap198) paralogs of other RSPs further diversifies RSs’ structure and perhaps function. The existence of paralogous RS proteins agrees with *Tetrahymena* genome analyses revealing duplication of some genes encoding proteins involved in sensing or structural complexity [66].

To the best of our knowledge, besides the *Chlamydomonas pf14* mutant, our cryo-ET analyses of *Tetrahymena RSP3* mutant cilia are the only ones showing ultrastructural changes caused by RSP3 knockout. Thus, using a combination of genetic and microscopic approaches, we have proved for the first time that Rsp3 is not an RS3 component. Previous studies of *Tetrahymena CFAP61*, *CFAP91*, and *CFAP251* knockout mutants engineered in our lab led to the identification of proteins that built the RS3 base and a part of the RS3 stalk [13, 19]. Based on the all available data, showing that CFAP61, CFAP91, and CFAP251 do not form a complex existing on its own but build a part of RS3, we propose to avoid the term “CSC complex” as it is misleading.

Presented here studies, available from 2023 as a reviewed preprint [67], led to the identification of additional proteins, which were either absent or diminished in cilia lacking RS3, including (i) two LRRC23-like proteins, (ii) AKAP domain-containing Rsp7B, (iii) adenylate kinases Ak7A, Ak7B, and Ak9, (iv) guanylate kinase Gk1, (v) phosphodiesterase, and (vi) one of the casein kinase 1 orthologs, strongly suggesting that these are either RS3 components or enzymes that dock to RS3. Our hypothesis agreed with the later proposed models of RS3 in mammalian sperm flagella and respiratory cilia locating AK9, two AK7, and two LRRC23 within the RS3 head [18, 31]. More recent reexamination of our proteomic data revealed that the MORN domain-containing protein, MRNN04, orthologous to the modeled RS3 subunit, MORN5, is also diminished in the *Tetrahymena* RS3 mutant. Models of mammalian RS3 suggest that a dimer of malate dehydrogenases, pseudophosphatase STYXL1, and CATIP also contribute to RS3 structure [18, 31]. The *Tetrahymena* genome encodes four malate dehydrogenases highly similar to mammalian enzymes. Only two of them were identified in the *Tetrahymena* ciliomes, but neither of the *Tetrahymena* malate dehydrogenases was detected in our co-IP or BioID assays. Moreover, neither of them was diminished in the *Tetrahymena* RS mutant; in contrast, one of them was moderately elevated in the *RSP3B* and *RSP3C* knockouts. Interestingly, another protein, TTHERM_00770670, which is similar to mouse MDH1B but only within the very N-terminal end (1–66 amino acids), significantly diminished in the CFAP91-KO mutant but was detected in Lrrc23A- and Lrrc23B-TurboID assays only by a single peptide. We did not identify *Tetrahymena* protein orthologous to STYXL1. The guanylate kinase, GK1, was suggested to be orthologous to CATIP ([31], Supplementary Table 1). However, only the very N-terminal, 60-amino acid fragment preceding the guanylate kinase domain is similar to a fragment in the middle part of CATIP.

The comparative proteomic analyses of *Tetrahymena* NaCl-extracted WT and CFAP61-KO axonemes revealed 49 RS3 or RS3-associated protein candidates conserved in mice, including IDA components, dynein heavy chains and p28 [31]. A search of our TMT and LFQ databases, each prepared based on three independent experiments, confirmed a significant reduction of only 15 of such proteins in the CFAP91-KO, RS3-less mutant.

Similar to RS3, the RS1 and RS2 are also composed of the evolutionarily conserved and species-specific components (*Chlamydomonas* [15], mammals [2, 5, 14, 18, 20], and *Trypanosoma* [32]). For example, RSP5 is a *Chlamydomonas*-specific RS head component, while TbRSP63 and TbRSP96 are *Trypanosoma*-specific. Moreover, *Tetrahymena* MORN domain-containing RS head proteins, Rsp1 and Rsp10, are relatively small (221 aa and 227 aa, respectively). While *Chlamydomonas* RSP10 and mammalian RSP1 are of similar size, *Chlamydomonas* RSP1 and mammalian RSP10 are four times larger (814 aa and 876 aa, respectively).

Damaging mutations in genes encoding RS1 and RS2 components cause PCD [21, 23, 44, 68–71], while mutations in genes encoding RS3 proteins, *CFAP61* [37, 38], *CFAP91* [39], *CFAP251* [36], and *LRRC23* [16, 40] result in male infertility but not PCD. The exception is the RS1 base protein, CFAP253/IQUB, whose mutation also causes male infertility but not PCD [14, 20]. Accordingly, in mice, Cfap61, Cfap91, Cfap251, Lrrc23, and Iqub/Cfap253 are highly expressed in the testis but not in the lung or brain [20, 38, 40]. Thus, perhaps RSs (especially RS3) contribute differently to the regulation of sperm flagella and multiciliated cell cilia motion. Based on data presented here and previous *Tetrahymena* mutants’ analyses [13, 19], it seems that *Tetrahymena* cilia, with their complex 3D waveform, are more similar to mammalian flagella than to cilia of multiciliated cells.

Interestingly, in humans, mutation in AK9 also causes male infertility [72], while mutations in AK7, besides male infertility [73], were correlated with PCD [74, 75] and caused PCD in mice [76]. Data obtained in *Xenopus* multiciliated cells (MCC) suggest that deletion of AK7 impairs cilia beating and mucociliary fluid flow due to centriolar defects and, in consequence, a reduced number of basal bodies [77]. Our proteomic data indicated that AK7A, besides RS3, docks to or is a component of other ciliary structures, and thus elimination of AK7 in mammals could affect other structures besides RS3, causing PCD.

*Tetrahymena* Rsp3 paralogs differently contribute to the RS structure and are preferentially recognized by other Rsp and/or RS-interacting/associated proteins. In RSP3A-KO and RSP3B-KO mutants, the RS1 base remains intact, and the level of RS1-base protein, Cfap253 [15, 20] is similar to that in WT cilia. Strikingly, the deletion of *RSP3B* but not *RSP3A* or *RSR3C* reduces the level of Cfap207 and the RS2-base component, Cfap206. Moreover, the deletion of *CFAP206* has a stronger impact on the level of Rsp3C than Rsp3B, together suggesting that Rsp3C could dock to Cfap206, which in turn is perhaps partly stabilized by Rsp3B. In *Tetrahymena*, the RS2 is docked to the A-tubule by front, side, and back prongs, and knockout of *CFAP206* in the vast majority of subtomograms (83%) eliminates the entire RS2 and front and back prongs. In the remaining 17%, RS2 was nearly unchanged while the front prong was missing and the back prong was reduced [41]. Interestingly, the number of RS2 remaining in *CFAP206* and *RSP3C* knockouts is similar. Based on the above data, it is also possible that the side prong maintained in CFAP206-KO cilia could be an Rsp3B dimer-dependent structure.

It is proposed that the RS head can transiently interact with CA projections and that such interactions enable the transmission of signals from the central apparatus via RS to dynein arms. These interactions can be mechano-chemical and electrostatic in nature [1–3, 15]. Compared to the Rsp3A and Rsp3B, the C-terminal end of Rsp3C is enriched in glutamic acid residues. If the Rsp3C C-terminal end, similar to the *Chlamydomonas* RSP3, is a part of the RS head, its presence increases the negative charge of the head surface and thus might locally modulate the electrostatic interactions with CA projections. Interestingly, the N-terminal part of Rsp3C is also unusual in having an ARF domain, which could play a role in signaling.

Our data suggest that the heads of the RS1 and RS2 are likely built of the same proteins (except for Rsp4B, likely co-assembling with Rsp3C), while the RS3 head contains Lrrc23 paralogs. These data agree with observations in mice and humans carrying mutations in Rsph1/RSPH1 or Rsph4A/RSPH4A showing that in multiciliated cells, only RS1 and R2 spokes are headless [21, 23] as well as recent findings that Lrrc23 mutation results in headless RS3 [16], and that Lrrc23 is an RS3 component as proposed by the RS3 model [18, 31].

Whether heads of all three RS interact with CA projections in a similar way and if all CA projections are involved in such interactions, is still an open question. Based on the fitted multi-scale axonemal structure, Meng and co-authors proposed a rigid contact mode between C1d and Rsph4a of doublet 8 and an elastic contact between C2b-C2d and Rsph1 of doublet 4 [2]. Our BioID data indicate that during cilia beating, the BirA* ligase is close enough to the central apparatus to biotinylate some of the CA components (Supplementary Table 11). Interestingly, Cfap46 and Cfap54, both subunits of C1d projection [78], were biotinylated when BirA* was fused to Rsp3A, Rsp4A, or Rsp4C. In contrast, in cells expressing Lrrc23-HA-BirA* fusions, Cfap54 was more prominently biotinylated than Cfap46. Moreover, in Lrrc23-BirA*-expressing cells, hydin and Cfap47, the C2b components [79], and Spef2A, and androglobin (Adgl), the C1b subunits [80], were also biotinylated. According to the model of *Chlamydomonas* CA, FAP46 and FAP54 form the outer surface of C1d, while CPC1/SPEF2 and FAP42 build the distal part of C1b [81, 82]. We previously showed that Adgl has limited similarity to FAP42 [80] and therefore likely has a similar position within the C1b projection. Taken together, the above data support the existence of interactions between the head of RS1 and RS2 and C1d as well as the RS3 head and C1d and long projections, C1b and C2b. The subunits of other projections, if biotinylated, were identified by a lower number of peptides. The proteomic analyses of respiratory cilia from WT and AK7-deficient mice (Supplementary Table 3 in [31]) suggested that the level of Hydin and C1b projection components are reduced in mutant mice cilia. Such changes were not observed in *Tetrahymena* CFAP91-KO mutant. Together, this raises a question about the involvement of the remaining CA projections in CA-RS interactions, calling for further studies.

### Differences in IDA Docking

Destabilization of RSs affects docking of some IDAs. In the RSP3B-KO mutant, the level of Dyh10, 12, and 25 was significantly reduced, which coincided with the lack of dynein c (Fig. 3). Dynein c (cryo-ET) and Dyh10, 12, and 25 (proteomic studies) were also missing in *Tetrahymena* cells with knocked out *CFAP206* encoding RS2-base protein ([41] and in this work). Strikingly, another RS2 dynein, dynein e, is not affected in RSP3B-KO or CFAP206-KO cilia, suggesting differences in the dynein c and dynein e docking. Indeed, the atomic model of *Chlamydomonas* RS2 shows that IDAc docks to the RS2 through FAP207 and p28, the IDAc subunit [15]. In *Tetrahymena* RSP3B-KO and CFAP206-KO mutants, the level of Cfap207 is reduced (Supplementary Table 7), strongly suggesting that also in *Tetrahymena* docking of IDAc depends upon Cfap207.

We did not find IDA defects in RSP3A-KO and RSP3C-KO mutants affecting RS1 and RS2, respectively. These observations agree with the atomic models of *Chlamydomonas* RS1 and RS2, suggesting that although FAP207 is present at the base of both RS1 and RS2, the p28 dimer of IDAa interacts with RS1-specific FAP253 [15]. Accordingly, in *Tetrahymena*, the level of Cfap253 remains unaltered in all studied RS mutants.

In *Tetrahymena*, knockout of *CFAP91* primarily affects the RS3 and the base of RS2, as the levels of Cfap206 and Cfap207 are substantially diminished ([13], and this work). In CFAP91-KO cilia, besides Dyh10, 12, and 25 (dynein c), the levels of Dyh16, 22, and 24 are moderately reduced ([13] and this work), suggesting that these dynein heavy chains are components of IDAd or IDAg. Of note, Dyh22p and Dyh15p co-precipitate with GFP-tagged RS3-base protein, Cfap251 [19]. Some of the remaining dyneins could be the components of IDAa or IDAb. Of note, in growing *Tetrahymena* cells, Dyh13, 17, 18, and 23 are expressed at a very low level (TGD, Gene expression profiles, and our proteomic data) and thus are likely minor components of *Tetrahymena* dynein arms or expressed during a specific life phase.

Out of three *Tetrahymena* p28 paralogs [61], p28B is likely the main, if not the only, p28 ortholog present in RS2 base-docked IDAc. In CFAP91-KO cilia, besides p28B, the level of p28A is also reduced [13]. Thus, p28A is likely a subunit of IDAd, but it remains to be determined if it forms homo- or heterodimers with p28B.

### RS proteins containing predicted enzymatic domains

The global comparative analyses of WT and RS mutant ciliomes led to another interesting discovery – the identification of several enzymes whose level was reduced in RS mutants, suggesting that those enzymes are either RS components or dock to specific RSs. Among them are (i) adenylate kinases that, together with arginine kinase(s), locally regulate ATP homeostasis; (ii) enzymes playing a role in the cGMP cycle; and (iii) serine-threonine kinases that could play a role in the control/regulation of RS-mediated signal transduction. Thus, the phenotypic outcome of the deletion of genes encoding RS structural proteins likely is not a sole consequence of the RS damage and some IDAs’ defects but also changes in protein phosphorylation-mediated signaling, the level of guanylate nucleotides, and the accessibility to the ATP-stored energy.

Adenylate kinases locally control the ATP levels and maintain the homeostasis of adenine nucleotides [57]. We have found that the levels of specific adenylate kinases were reduced in specific RS mutants. The *Chlamydomonas* RS2 protein, FAP385, was modeled as an RS neck subunit [15]. FAP385 is highly similar to *Tetrahymena* Ak8A, which suggests that Ak8A and likely Ak8B are subunits of RS2 and RS1 spokes, respectively. By analogy, Ak7A, Ak7B, and Ak9 could be the components of RS3. Such an assumption agrees with RS modeling data [2, 18, 31]. Of note, Meng and co-authors [2] modeled AK8 to the RS stalk, near the Rsph20 and Rsph22, while Leung and co-authors [18] proposed that, similar to FAP385 [15], AK8 is a part of the RS neck. Such differences call for wet-bench experiments verifying AI-based predictions.

Strikingly, the presence of Ak7B, Ak8B, and Ak9 in cilia nearly completely depends upon the presence of a certain class of RS, suggesting that RSs are their sole localization sites, while Ak1, Ak7A, and Ak8A can also associate with or be a part of other ciliary structures. Interestingly, Gk1 and Pde enzymes were also nearly completely eliminated from CFAP91-KO cilia, suggesting their docking or presence near RS3 or its vicinity. Gk1 is one of two *Tetrahymena* guanylate kinases. Gk catalyzes phosphate transfer from ATP to GMP, producing GDP and ADP. The phosphorylation of GDP to GTP is catalyzed by nucleoside-diphosphate kinases (NDK), and GTP can be converted to cGMP by guanylate cyclase, while 3^’^5’-cyclic nucleotide phosphodiesterase (PDE) hydrolyzes cGMP [83–85]. The RSP23 has NDK domains [51], however, its level remains unchanged in analyzed mutants. Early analyses of the ciliary beating in *Paramecium* showed that cyclic nucleotide monophosphates-driven chemical signaling controls ciliary beat [86–88]. Thus, the immotility of the CFAP91-KO mutants might be caused not only by the lack of RS3 and some IDAs [13] but also by the perturbation of the guanidine nucleotide homeostasis.

It is tempting to speculate that some RSPs undergo phosphorylation and that the phosphorylation status controls RS-mediated transduction of the regulatory signals. We found that the level of one of the casein kinase 1-type enzymes is completely eliminated in the *CFAP91* mutant, while the levels of some cAMP-dependent protein kinase (PKA) subunits are altered in RS mutants. The presence of Ck1 in the RS3 vicinity was confirmed by co-IP and BioID studies. In *Chlamydomonas*, CK1 docks to outer doublets and regulates the phosphorylation of IC138, the IDAI1/f intermediate chain [89]. In *Tetrahymena*, this role can be played by other Ck1-type enzymes.

## Materials and methods

### *Tetrahymena* cell culture

*Tetrahymena thermophila* (Tetrahymena Stock Center) cells were grown to the mid-log phase (2-4 × 10^5^ cells/mL) with shaking (80–110 rpm) at 30 °C. WT cells and motile mutants were cultured in a standard SPP medium (1% proteose peptone, 0.1% yeast extract, 0.2% glucose, 0,003% Fe-EDTA) [90], while mutants with major ciliary defects were grown in a rich MEPP medium (2% proteose peptone, 2 mM Na-citrate, 1 mM FeCl_3_, 30 µM CuSO_4_, 1.7 µM Folinic acid, Ca salt) [91], both supplied with an antibiotic-antimycotic mix (Sigma-Aldrich, St. Louis, MO, USA) at 1:100 (SPP) or 1:50 (MEPP). Before biolistic transformation and BioID assay, cells at mid-log phase were washed in 10 mM Tris pH 7.4 and next grown overnight (14–22 h) in the same buffer.

### Generation of *Tetrahymena* mutants

Engineering and phenotypic analyses of *Tetrahymena* mutants with deleted *CFAP61* [19], *CFAP206* [41], or *CFAP91* [13] were described before. The *RSP3* genes’ fragments used to obtain knock-out and knock-in transgenes were amplified by PCR with the addition of restriction sites using Phusion™ Hot Start II DNA high-fidelity polymerase (Thermo Fisher Scientific) and the appropriate primers listed in Supplementary Table 13.

### Knock-outs

To delete a part of the *RSP3* genes (0.8-1 kb), approximately 1.2–1.5 kb fragments positioned upstream and downstream of the targeted gene fragment were amplified and cloned subsequently on both sides of the neo4 resistance cassette [92]. Approximately 60 µg of plasmid was digested with ApaI and SacII to separate the transgene from the plasmid backbone and precipitated onto approximately 0.63 mg of 0.5–0.8 nm gold particles (Thermo Fisher Scientific) by mixing with 1 M CaCl_2_ and 20 mM spermidine (final concentrations). DNA-coated gold particles were washed with ethanol and placed on macrocarriers (Bio-Rad), and after drying, used to transform conjugating *Tetrahymena* cells (strains CU428 and B2086) at the early stages of meiosis. Transformed cells were selected in SPP medium supplemented with 1.5 µg/mL CdCl_2_ and 100 µg/mL paromomycin, followed by selection in 6-methylpurine (15 µg/mL)-supplemented SPP. Double resistant cells, after sexual maturation and validation by crossing to CU427 cells, were crossed to the infertile A*III strain to obtain heterokaryons. Next, heterokaryons were crossed to obtain knockout cells [93, 94]. The deletion of the targeted fragments was confirmed by PCR.

To eliminate a fragment of the RSP2 gene, we used a co-deletion approach [95] and primers listed in Supplementary Table 13. The targeting plasmid (~ 15 µg) was introduced to conjugating CU428 x CU427 cells using a biolistic gun as described above. After 14 h, cells were transferred to SPP medium, grown for 6 h, and exposed to paromomycin selection (100 µg/mL) on 96-well plates. To verify *RSP2* gene fragment deletion, the genomic DNA was purified from the WT (control) and paromomycin-resistant cells, and the extent and completeness of gene deletion was analyzed by PCR using primers listed in Supplementary Table 13.

### Knock-ins

To express RS proteins as fusions with C-terminal − 3HA, -HA-BirA*, or -HA-TtBCCP tags under the control of the transcriptional promoter, approximately 1 kb fragments of the open reading frame immediately upstream of the STOP codon and ~ 1 kb fragment of the 3’UTR were amplified by PCR as described above (primers used are listed in Supplementary Table 13) and cloned into pCFAP44-3HA-neo4, pCFAP44-HA-BirA*-neo4, and pCFAP44-TtBCCP-neo4 plasmids, respectively [96, 97] to replace fragments of the *CFAP44* gene. To express Lrrc23 with a C-terminal TurboID tag (TurboID) we amplified the Turbo coding region from the IFT52-Turbo plasmid adding restriction sites, and replaced the BirA* coding region in the pLRRC23A- HA-BirA*-neo4 plasmid. To express Rsp4 proteins with an N-terminal BirA*-HA tag, we replaced 5’UTR and coding region fragments of Adgb in the BirA*-HA-Adgb plasmid [80] with those of RSP4 genes. To express proteins with a C-terminal GFP tag under the control of the transcriptional promoter and pPur cassette enabling selection of transformed *Tetrahymena* cells with puromycin [98], the coding sequence of the 2xV5 tag in the pCFAP44-2V5-pPur plasmid [97] was replaced by the GFP coding region, and fragments of the *CFAP44* gene were replaced by the fragments of a coding region and the 3’UTR of the gene of interest as described above. Approximately 10–15 µg of plasmid was digested with MluI and XhoI to separate the transgene from the plasmid backbone and precipitated onto approximately 0.20 mg of 0.5–0.8 nm gold particles (Thermo Fisher Scientific) as described above. Approximately 10^7^ CU428 cells were transformed by biolistic transformation, and after 2 h recovery in SPP medium supplied with 1.5 µg/mL CdCl_2_, cells were transferred to 96-well plates, and transformants were selected using 100 µg/mL paromomycin for 3–4 days.

To co-express Rsp3B-3HA with Rsp3A-GFP or Rsp3C-GFP, Rsp3B-3HA cells were transformed with appropriate constructs as described above except that after transformation cells were grown for 24 h in an SPP medium supplied with 2.5 µg/mL CdCl_2_. Positive transformants were selected on 96-well plates using 200 µg/mL puromycin. After selection, transformants and double transformants were grown in SPP medium supplied with the growing concentration of paromomycin and/or puromycin and reduced concentration of CdCl_2_ to promote transgene assortment [99].

### Phenotypic analyses

The measurements of the cell swimming rate and the analyses of cilia beating (amplitude, waveform, and frequency) were described in detail [13]. Briefly, for swimming rate analyses, cells at a density of 2-3 × 10^3^ cells/mL were viewed and recorded at room temperature using a Zeiss Discovery V8 Stereo microscope (Zeiss, Oberkochen, Germany) equipped with a Zeiss Plans 10_ FWD 81 mm objective and an Axiocam 506 camera and ZEN2 (blue edition) software. The length of the trajectories was measured using ImageJ software (Supplementary Table 14 for Source data) and the colored lines parallel to the trajectories were added in the Adobe Photoshop program. For each strain, experiments were done in triplicate and 80–100 trajectories were registered and analyzed in each experiment. Cilia beating was analyzed as described in [13]. Cells from a mid-log phase were cultured at room temperature for 3 hours, centrifuged, placed between two pieces of adhesive tape fixed on the glass slide, covered with a coverslip, and recorded using a Phantom Miro C110 high-speed camera (Vision Research, Wayne, NJ, USA) mounted on an AXIO Imager M2 microscope (Zeiss, Germany) with either a 40 x oil immersion lens (analyses of cilia beating frequency) or a 63x oil immersion lens (numerical aperture 1.4, analyses of ciliary waveform). Videos were recorded at 900 frames/s. For each strain, at least 10–15 cells were recorded, aligned in ImageJ, and analyzed using ImageJ (frequency, Supplementary Table 15 for Source data) or Adobe Photoshop (waveform, amplitude).

### Immunofluorescence

To analyze the localization of HA-tagged Rsp proteins, cells from the overnight culture were fixed 1:1 v/v on coverslips with a mix of 1% Triton and 4% PFA/or 1% NP-40 substitute and 4% PFA, both in a PHEM buffer (12 mM PIPES, 5 mM HEPES, 2 mM EGTA, 0.8 mM MgSO_4_, pH 6.9). After drying and blocking with 3% BSA in PBS, cells were stained overnight at 4 °C with a mix of primary antibodies (i) rabbit monoclonal anti-HA antibody (BioLegend, San Diego, CA, USA) 1:300 and mouse monoclonal anti-acetylated α-tubulin antibody 6–11 B1 (1:2000) or (ii) mouse monoclonal anti-HA 16B12 (1:200) and rabbit polyclonal polyG (1:2000) [100]. To co-localize Rsp3 paralogs, cells were stained with a mix of primary antibodies: mouse monoclonal anti-HA 16B12 (1:200) (BioLegend, San Diego, CA, USA) and rabbit polyclonal anti-GFP (1:6000) (Abcam). After washing with PBS, samples were stained for 1.5 h at RT with a mix of the secondary antibodies, anti-mouse and anti-rabbit IgG, conjugated with either Alexa-488 or Alexa-555 (Invitrogen, Eugene, OR, USA) both diluted 1:300. Coverslips were mounted in Fluoromount-G (Southern Biotech., Birmingham, AL, USA). Cells were recorded using either a Zeiss LSM780 (Carl Zeiss Jena, Germany) or a Leica TCS SP8 (Leica Microsystems, Wetzlar, Germany) confocal microscope.

### Deciliation of *Tetrahymena* cells

For immunofluorescence analyses of regenerating cilia, cells co-expressing 3HA- and GFP-tagged Rsp3 paralogs were grown overnight to mid-log phase and rinsed with 10 mM Tris-HCl, pH 7.4 buffer. Approximately 4 × 10^5^ cells were collected, suspended in 100 µL of a 10 mM Tris-HCl, pH 7.4 buffer and mixed with 1 mL of 10% Ficoll in 10mM Tris-HCl, pH 7.5, and immediately deciliated by passing 5–6 times through the syringe needle (diameter 0.8 mm). After 60–90 s, cells were transferred to 10 mL of SPP medium for cilia regrowth.

For biochemical analyses, cilia were purified from 6 to 8 × 10^7^ cells using a pH shock method [101]. In brief, cells were collected, rinsed with Tris-HCl buffer, pH 7.4, resuspended in deciliation buffer (10 mM Tris-HCl, pH 7.4, 10 mM CaCl_2_, 50 mM sucrose), and deciliated by addition of acetic acid (10.5 mM final concentration). After 1–1.5 min the pH was raised to physiological level with potassium hydroxide (10.8 mM final concentration). Deciliation was monitored under a microscope. Deciliated cell bodies were separated from cilia-containing supernatant by centrifugation (twice at 1680 x g for 5 min) and cilia were collected by centrifugation at 26,900 x g for 30 min at 4 °C. Next, cilia were resuspended in a cold deciliation buffer supplemented with protease inhibitors (cOmplete mini EDTA-free protease inhibitor cocktail, Roche Diagnostics GmbH, Mannheim, Germany), and protein concentration was estimated using Pierce™ BCA Protein Assay Kit (Thermo Scientific, Bartlesville, OK, USA). Purified cilia were further analyzed using Western blotting, mass spectrometry (ciliomes), co-immunoprecipitation, or BioID assays.

### Co-immunoprecipitation and proximity labeling (BioID) assays

All buffers used in biochemical studies were supplemented with protease inhibitors (cOmplete mini EDTA-free protease inhibitor cocktail, Roche Diagnostics GmbH, Mannheim, Germany).

For the co-immunoprecipitation assay, approximately 6 × 10^7^ cells from a mid-log phase culture, either WT (control) or expressing − 3HA tagged Rsp proteins, were spun down and washed with 10 mM Tris-HCl buffer (pH 7.4). After deciliation, collected cilia were resuspended in deciliation buffer and combined with an equal volume of 2% NP-40 and 1.2 M NaCl in 80 mM Tris-HCl buffer, pH 7.5. After 15 min incubation on ice, axonemes were pelleted at 21,000 × g, 4 °C, for 15 min and treated with 0.5 M KI, 30 mM NaCl, 5 mM MgSO_4_, 0.5 mM EDTA, 1 mM DTT in 10 mM HEPES, pH 7.5. After 30 min on ice, the axonemes were centrifuged (21,000 × g for 15 min at 4 °C). The supernatants were diluted 500x with 50 mM Tris–HCl, pH 7.4 and concentrated on ultracentrifugation columns (Vivaspin^®^ Turbo 4, Sartorius, Niemcy). Collected proteins (0.5-1 mg) were incubated overnight with agarose beads-conjugated anti-HA antibody (Thermo Fisher Scientific, Waltham, MA) at 4 °C. The bead-bound proteins were identified by mass spectrometry (Supplementary Table 16 for Source data).

The proximity-labeling (BioID) assay [102]was performed as described in detail [80]. The WT cells were used as a control because the ~ 30 kDa BirA* tag can enter cilia by diffusion and randomly biotinylate components of different ciliary complexes. Briefly, approximately 6 × 10^7^ WT or Rsp-HA-BirA fusion-expressing cells were grown to the mid-log phase, transferred for 16–18 h to 10 mM Tris-HCl buffer (pH 7.4) and next incubated with 50 μm biotin for 4 h at 30 °C in the same buffer. After deciliation, cilia were suspended in axoneme stabilization buffer (20 mM potassium acetate, 5 mM MgSO_4_, 0.5 mM EDTA in 20 mM HEPES, pH 7.5) supplied with 0.2% NP-40 to release unbound biotin. After 5 min, the axonemes were spun down (10 min, 21,000x g, 4 °C), washed, suspended in lysis buffer (50 mM Tris–HCl, pH 7.4, 0.4% SDS, 0.5 M NaCl, 1 mM DTT), and incubated at RT for 1 h. After spinning down (8000x g at 4 °C), the collected supernatant was diluted with 50 mM Tris–HCl, pH 7.4 (1:3) and incubated overnight with streptavidin-coupled Dynabeads (Dynabeads M-280 Streptavidin, Thermo Fisher Scientific, Waltham, MA) at 4 °C. The bead-bound biotinylated proteins were analyzed by Western blot using HRP-conjugated streptavidin (1:40 000) (Thermo Fisher Scientific, Rockford, IL, USA) and identified by mass spectrometry (Supplementary Table 17 for Source data).

### Protein gel electrophoresis

Approximately 30 µg of ciliary proteins or 10% of bead-bound proteins were loaded on the SDS-PAGE polyacrylamide gel, separated in standard conditions, and transferred to a nitrocellulose membrane for 1 h at 170 mA. Two-dimensional electrophoresis was performed as described [13]. Approximately 30 µg of ciliary extract purified from cells expressing Rsp-3HA fusion proteins were cleaned with ReadyPrep 2-D cleanup (Bio-Rad) and dissolved in rehydration buffer (7 M urea, 2 M thiourea, 2% CHAPS, 0.1%, Tergitol NP7, 40 mM DTT and 0.2% BioLytes). A protein solution was used to rehydrate ReadyStrip IPG pH 3–10 or 4–10 (Bio-Rad) for at least 12 h. Proteins were separated in Protean IEF Cell (Bio-Rad) for ~ 80kVh, at 4000 V and next subjected to standard SDS-PAGE and transferred to a nitrocellulose membrane.

### Western blot

After transfer to nitrocellulose and blocking for 1 h with a 5% skimmed milk in TBST, blots were incubated with mouse monoclonal anti-HA antibody (1:2000) or rabbit polyclonal anti-GFP antibody (1:60,000), both diluted in 5% skimmed milk in TBST (overnight, 4 °C). After washing (4 × 10 min, TBST) and 1 h incubation at RT with HRP-conjugated secondary antibodies: goat anti-mouse IgG (1: 10 000) (Jackson ImmunoResearch, West Grove, PA, USA) or goat anti-rabbit IgG (1:20,000) (Sigma-Aldrich) blots were washed as before. To analyze biotinylated proteins, nitrocellulose was blocked with 3% BSA in TBST, incubated with HRP-conjugated streptavidine (1:40,000 in 3% BSA in TBST, 3 h, RT) (Thermo Fisher Scientific) and washed again (4 × 10 min TBST). Proteins were visualized using a Westar Supernova kit (Cyanagen, Italy).

### Cryo-ET Preparation

The axonemes were cross-linked with glutaraldehyde (final concentration 0.15%) for 40 min on ice and quenched with 35 mM Tris pH 7.5. The axoneme solution at 3.6 mg/mL was mixed with 5 (Cytodiagnostics) or 10 (Aurion) nm gold beads in a 1:1 ratio for a final axoneme concentration of 1.8 mg/mL. Inside the Vitrobot Mk IV (Thermo Fisher) chamber, 4 µl of crosslinked axoneme sample was applied to negatively glow discharged (10 mA, 10 s) C-Flat Holey thick carbon grids (Electron Microscopy Services). The sample was incubated at 23 °C and 100% humidity for 45 s on the grid, followed by 8 s of blotting with force 0 and plunge frozen in liquid ethane.

### Cryo-ET acquisition and reconstruction

Tilt series were collected using the dose-symmetric scheme from − 60 to 60 degrees with an increment of 3 degrees at 2.12 Å per pixel using a Titan Krios equipped with Gatan K3 and BioQuantum energy filter. The acquisition was performed using SerialEM [103]. The defocus values for the tilt series range from − 2.5 to −6 μm. The total dose for each tilt series is 120 to 160 e- per Å^2^. For each view, a movie of 10–13 frames was collected. Motion correction of each view was performed with Alignframes [104]. Tomograms were reconstructed using IMOD [105].

### Subtomogram averaging

CTF estimation for each tilt series was performed with WARP [106]. The doublet in the tomograms was picked using IMOD by tracing the line along the microtubules [105]. Subtomogram averaging of the 4-times binned 96 nm repeating unit of WT and mutant strains was performed using the “axoneme align” program [107]. The subtomogram coordinates and alignment parameters were converted to Relion 4.0 for local refinement and classification [108]. The resolutions for the 96-nm repeating unit of the axoneme of wild type, *RSP3A-KO*, *RSP3B-KO*, and *RSP3C-KO* are 18, 20, 22, and 17 Å, respectively. Axonemal repeats from *RSP3A* and *RSP3B* mutants showed strong heterogeneity in the occurrence of RS1 and RS2. To analyze the heterogeneity of the mutant strains, three-dimensional (3D) classification without alignment was performed in Relion by masking RS2 and RS1. The axoneme of the *RSP3C* mutant strain showed heterogeneity in the RS2 head and neck region. Therefore, unsupervised 3D classification by masking the head and neck region was performed with three classes.

The 3D classification of *n* = 2099 axonemal 96-nm repeats with RS1 defects revealed either the lack of the entire RS1 (*n* = 639 units, ~ 30%) or RS1 except for the RS1 base (*n* = 1417, 67%). The RS1 in the remaining 43 units was not classified due to the low number of particles. The identification of intact RS1 in Relion was unsuccessful, likely due to the structural heterogeneity of intact RS1 in RSP3A-KO compared to WT and/or conformational flexibility enabling RS, especially RS1 to tilt [2, 6]. However, visual inspection of denoised tomograms revealed the presence of intact RS1 in ~ 22% of units (*n* = 455). Additionally, the averaged subtomogram map indicates that existing RS1 spokes are thinner in RSP3A-KO than in WT, suggesting potential structural differences associated with the knockout (Supplementary Fig. 6). In contrast, the RSP3B-KO mutant lacked RS2 in all analyzed axonemal units (*n* = 2092). Moreover, ~ 77% of the axonemal repeats (*n* = 1622) showed also RS1 defects; the RS1 structure was either missing (51%, *n* = 1078) or had only a base part (26%, *n* = 544). Among the collected *n* = 2790 axonemal repeats, 173 axonemal repeats (~ 7%) were unclassified. The analyses of the remaining *n* = 2617 axonemal repeats revealed that ~ 19% (*n* = 527) lacked the entire RS2. The 3D classification of the remaining repeats using the RS2 mask covering an RS2 head and stalk showed that the remaining units grouped into two categories: (i) with an intact RS2 structure (*n* = 469) and (ii) with a well-visible RS2 base (*n* = 1621) (Supplementary Fig. 6).

For visualization, tomograms were CTF deconvolved and missing wedge corrected using IsoNet [109]. UCSF ChimeraX was used for the visualization of subtomogram averages, surface rendering, segmentation, and fitting [110].

### Heat map

Heat maps illustrating the distribution of particles within each doublet were generated using Microsoft Excel. The doublet with the highest particle count was designated as the first doublet. To assess the distribution of subtomograms with intact RS2 across doublets, counts were normalized such that a value of ‘0’ indicates no subtomograms with intact RS2, while ‘100’ represents a doublet where all subtomograms have intact RS2. Tomograms were categorized into proximal and medial-distal regions based on the presence of the CCDC81B MIP signal. Tomograms exhibiting this signal were classified as proximal, while those lacking the signal were designated as medial-distal [111]. Bar graph visualization and statistical analyses were performed using GraphPad Prism (GraphPad Software, San Diego, CA, USA). Mean percentages of intact RS2 subtomograms for each doublet were calculated in GraphPad Prism, treating tomograms as biological replicates to account for biological variability. Statistical significance was assessed using an unpaired t-test, and error bars represent the standard deviation across tomograms.

### Differential quantitative cilia proteome analyses

#### Sample Preparation

Cilia purified from 5 × 10^7^ cells from mid-log phase culture were resuspended in 10 mM Tris pH 7.4 and protein concentration was determined with Pierce™ BCA Protein Assay Kit (Thermo Scientific, Bartlesville, OK, USA). Three hundred micrograms of protein were precipitated using a ReadyPrep 2-D Cleanup Kit (Bio-Rad Laboratories, USA). Protein pellets were dissolved in 0.1% RapiGest in 500 mM tetraethylammonium bromide (TEAB) and then incubated at 850 rpm for 45 min at 37 °C (Eppendorf Comfort Thermomixer, Eppendorf, USA). Proteins were digested by trypsin (Trypsin Gold, Mass Spectrometry Grade, Promega; protein: enzyme (w/w) ratio – 100:1, at 37 °C for 16 h) according to a standard protein digestion protocol including reduction (by 1,4-dithiothreitol) and alkylation (by iodoacetamide). The digestion reaction was stopped by the addition of 55% trifluoroacetic acid (final concentration of 5%), samples were centrifuged at 20,000 x g for 30 min at 4 °C to precipitate RapiGest, and pellets were discarded. Supernatants containing the obtained peptides were purified using Pierce™ Peptide Desalting Spin Columns (Thermo Scientific, Bartlesville, OK, USA) according to the manufacturer’s protocol, dried in a vacuum concentrator at RT (SpeedVac Concentrator Plus, Eppendorf, USA) and stored at −80 °C for further analysis.

#### LC‒MS/MS analysis of labeled peptides (TMT analysis)

Desalted peptides were dissolved in 100 µl of 100 mM TEAB solution, and peptide concentrations were determined using the Pierce™ Quantitative Fluorescent Peptide Assay (Thermo Scientific, USA). Next, a TMT labeling reaction was performed according to the procedure provided by the manufacturer (Thermo Fisher Scientific). Briefly, a volume of a sample containing 30 µg of peptides was labeled with a corresponding tandem mass tag. The labeling reaction was carried out for 1 h at room temperature and quenched with 5% hydroxylamine. Additionally, a test sample was prepared to check the efficiency of labeling. Labeled peptides were purified and fractionated using liquid chromatography at high pH. Separation was carried out for 26 min at a flow rate of 0.8 ml/min using a UPLC system (Acquity UPLC Class H system, Waters). The mobile phases consisted of water (A), acetonitrile (B), and 100 mM ammonia solution (C). The percentage of phase C was kept constant at 10% throughout the separation. Fractions were collected every 1 min, starting from the second minute of the run. The peptide elution was monitored spectrophotometrically at 214 nm. Twenty-four fractions were collected and combined to obtain 12 measurement samples. Samples were dried in a vacuum concentrator at room temperature. Peptides were resuspended in 100 µl of 5% acetonitrile and 0.1% formic acid.

Peptides were analyzed on the Evosep One system (Evosep Biosystems, Odense, Denmark) coupled to the Orbitrap Exploris 480 mass spectrometer (Thermo Fisher Scientific, USA) according to [112]. One µg of peptides was loaded onto Evotips C18 trap columns (Evosep Biosystems, Odense, Denmark) according to the manufacturer’s protocol with some modifications. Chromatographic separation of peptides was carried out using a mobile phase flow rate of 500 nl/min in gradient elution mode for 44 min on an EV1106 analytical column (Dr. Maisch C18 AQ, particle size 1.9 μm, 150 μm x 150 mm, Evosep Biosystems, Odense, Denmark). The following gradient elution was used: 0 min – 1% B, 120 min – 35% B, 121 min – 95% B, 124 min – 1% B, 127 min – 95% B, 130 min – 1% B. The eluted peptides were ionized in the positive ion mode in the nano-ESI source with a capillary voltage of 2.1 kV and a temperature of transfer capillary at 275 °C. Survey scans from 300 *m/z* to 1700 *m/z* were acquired by an Orbitrap mass analyzer (Thermo Fisher Scientific, Waltham, MA, USA) at a resolving power of 60 000. The resolving power in the MS2 spectrum measurement mode was 30 000 with the TurboTMT function set to TMT Reagents. HCD-MS/MS spectra (normalized collision energy of 30%) were generated for 25 multiply charged precursor ions from each survey scan. A precursor fit filter was applied to reduce peptide co-fragmentation. Dynamic exclusion was set to 20 s, and the precursor ion intensity threshold was set to 5 × 10^3^. Supplementary Table 18 for Source data.

#### LC-MS/MS analysis of non-labeled peptides (label-free analysis)

Desalted peptides were resuspended in 100 µl of 5% acetonitrile and 0.1% formic acid, and loaded on Evotips C18 trap columns and separated for 88 min as described above. Positive ionization and DDA (data-dependent acquisition) mode were applied to collect data. MS1 parameters were: resolving power 60,000, AGC target 300%, and the m/z range of 300 to 1600. MS2 parameters were: resolving power 15,000, normalized AGC target. Forty of the most abundant precursor ions within an isolation window of 1.6 m/z were fragmented. The intensity threshold was set up at 5 × 10^3^. The higher energy collisional dissociation mode with a normalized collision energy of 30% was applied for precursor ion fragmentation.

### Data analysis

MS data were analyzed with FragPipe (v. 17.1) (Nesvilab, University of Michigan, Ann Arbor, MI), MSFragger (v. 3.4) [113] and Philosopher (v. 4.2.1) [114]. ProteoWizard’s MSConvert (v. 3.0.1908) (Palo Alto, CA) [115] was used to convert the raw MS data to mzML format. The *Tetrahymena thermophila* UniProt database (canonical and isoform sequences; 27,027 entries) was searched using the following search parameters: (i) digestion enzyme-trypsin/P, up to two missed cleavage sites were allowed, (ii) precursor and fragment ion mass tolerance ± 10.0 ppm and ± 20 ppm, respectively, (iii) fixed modifications: carbamidomethyl (C), (iv) variable modifications: oxidation (M), deamidation (N), (Q). For TMT samples, additional fixed modification was used (v) TMT modification of lysine (+ 229.16293) and variable modification (vi) TMT modification of N-termini of protein and peptide (+ 229.16293). Proteins and peptides were identified using the target-decoy approach with a reversed database. The peptide mass range was set from 500 Da to 5 000 Da. The results were processed with FDR (false discovery rate) set to 1% at the PSM, peptide and protein levels. Quantitative analysis was performed using IonQuant (label-free quantitation) and TMT Integrator (TMT-based quantitation). Statistical analysis was performed with Perseus (v. 2.0.3) (Max Planck Institute of Biochemistry, Martinsried, Germany). In label-free quantitation, missing values were replaced based on quantile regression imputation of left-censored data (QRILC) [116]. Student T-test was used for statistical analysis. Proteins were considered to be differentially expressed if the difference in abundance was statistically significant (FDR adjusted p-value < 0.05) and the fold change was equal to or higher than 1.5. Supplementary Table 19 for Source data.

### Reagents

All reagents used and the reagent source are provided in Supplementary Table 20.

### Quantification and statistical analyses

Data are given as mean +/- SD and were compared via two-tailed distribution Student’s t-tests.

For statistical analysis, a t-student test was used in the majority of experiments. Perseus (v. 2.0.3) program was used for statistical analysis of TMT proteomic data.

## Supplementary Information

Below is the link to the electronic supplementary material.

Supplementary Material 1

Supplementary Material 2

Supplementary Material 3

Supplementary Material 4

Supplementary Material 5

Supplementary Material 6

Supplementary Material 7

Supplementary Material 8

Supplementary Material 9

Supplementary Material 10

Supplementary Material 11

Supplementary Material 12

## Data Availability

All plasmids and Tetrahymena mutants are available on request from Dorota Wloga. All source data are provided as Supplementary Tables.
