# Supplementary figures and images for: Heterogeneity of radial spoke components in Tetrahymena cilia
Source: Cell Mol Life Sci. 2025 Aug 31;82(1):329. doi: 10.1007/s00018-025-05871-x (PMC12399478; doi:10.1007/s00018-025-05871-x)

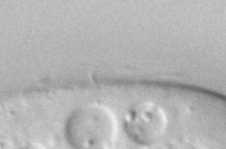

Supplement: Supplementary file 1 — Supplementary Material 1 [file 18_2025_5871_MOESM1_ESM.gif]

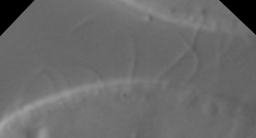

Supplement: Supplementary file 2 — Supplementary Material 2 [file 18_2025_5871_MOESM2_ESM.gif]

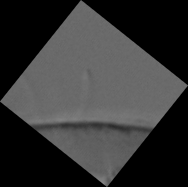

Supplement: Supplementary file 3 — Supplementary Material 3 [file 18_2025_5871_MOESM3_ESM.gif]

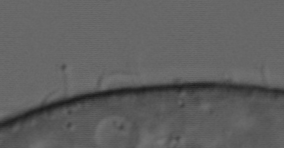

Supplement: Supplementary file 4 — Supplementary Material 4 [file 18_2025_5871_MOESM4_ESM.gif]

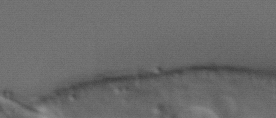

Supplement: Supplementary file 5 — Supplementary Material 5 [file 18_2025_5871_MOESM5_ESM.gif]
