# Supplementary material for: Heterogeneity of radial spoke components in Tetrahymena cilia
Source: Cell Mol Life Sci. 2025 Aug 31;82(1):329. doi: 10.1007/s00018-025-05871-x (PMC12399478; doi:10.1007/s00018-025-05871-x)
Supplement: Supplementary file 12 — Supplementary Material 12 [file 18_2025_5871_MOESM12_ESM.pdf]

Supplementary Information

Heterogeneity of radial spoke components in *Tetrahymena* cilia.

Marta Bicka[1,2#], Avrin Ghanaeian[3#], Corbin Black[3#], Ewa Joachimiak[1], Anna Osinka[1], Sumita Majhi[1], Anna Konopka[4,5], Ewa Bulska[2,4*], Khanh Huy Bui[3*], Dorota Wloga[1*]

Table of content

Supplementary Figures……………………………………………………………….2 - 27

Supplementary Tables……………………………………………………………….28 - 49

Supplementary Movies………………………………………………………………49

References…………………………………………………………………………49 - 50

Supplementary Figures

A

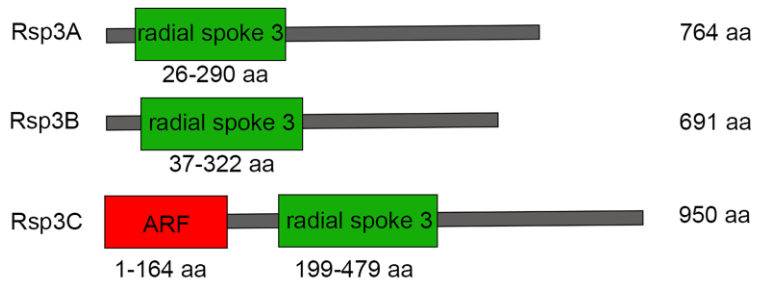

B

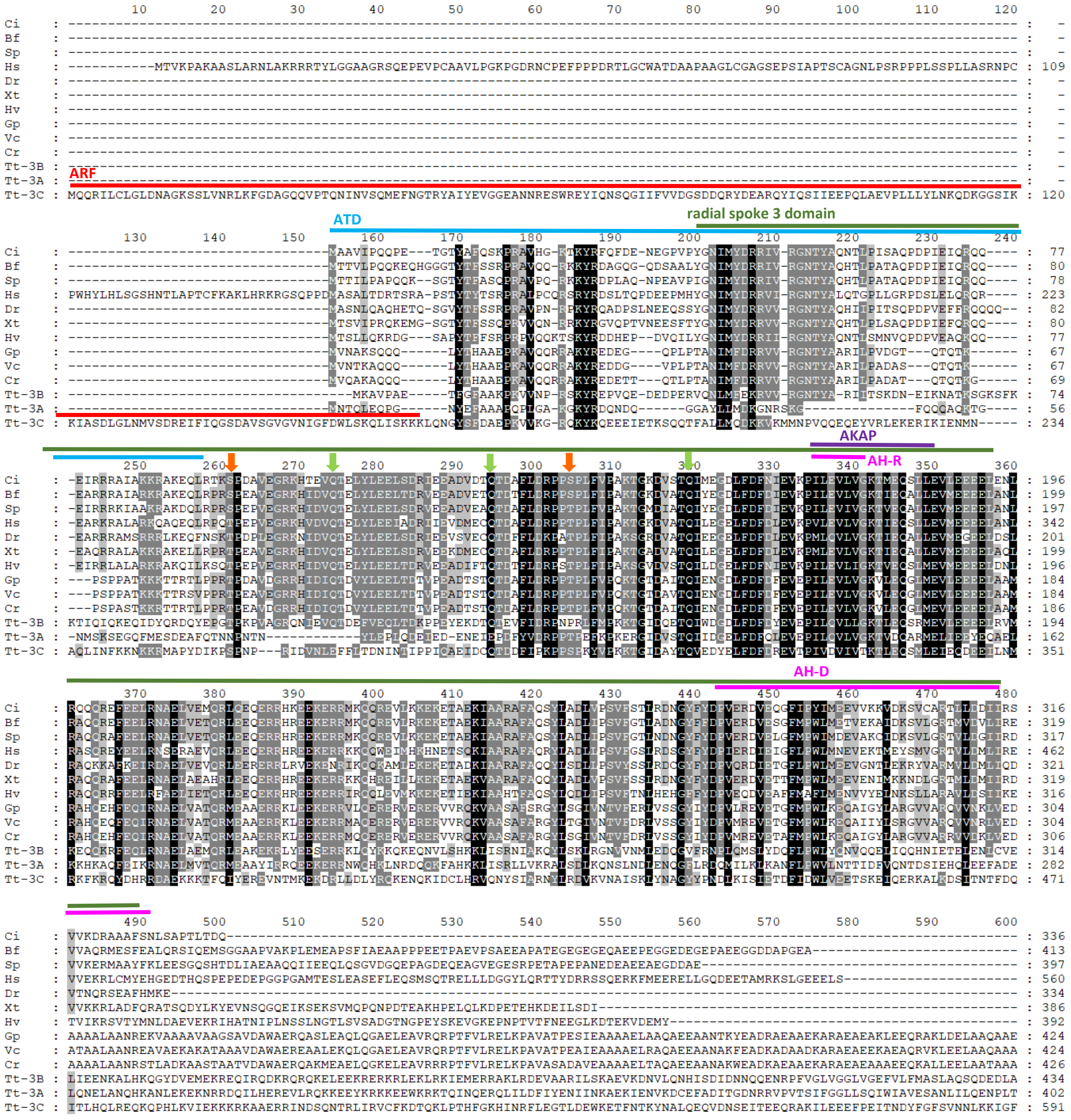

```
                610       620       630       640       650       660       670       680       690       700       710       720
Ci     : ------------------------------------------------------------------------------------------------------------------------ :   -
Bf     : ------------------------------------------------------------------------------------------------------------------------ :   -
Sp     : ------------------------------------------------------------------------------------------------------------------------ :   -
Hs     : ------------------------------------------------------------------------------------------------------------------------ :   -
Dr     : ------------------------------------------------------------------------------------------------------------------------ :   -
Xt     : ------------------------------------------------------------------------------------------------------------------------ :   -
Hv     : ------------------------------------------------------------------------------------------------------------------------ :   -
Gp     : AAAERGEDPPEEPPQLDE--PLVDVEAVVAAAADAVQRPPVREVADIDILSYMLDKGIVTKDAIIQSLAVHALGDKAYVNHPAFADSS--------------------------------- : 510
Vc     : EAEERGEEPPEEPPTLDE--PLVDVEAVVAAAVEAVVKPPVKEVEDIDIISYMLDQGIITKDAIIQSLAVHALGDKAYINHPAFAEGA-------------------------------- : 510
Cr     : EAEERGEEPPAEPPSLPDGVEPVDVEAEVAKAVEAVPKPPVKEVTDIDILSYMMDKGAITKDAIIQALAVHALGDKAYTNHPAFAEAEGA------------------------------ : 516
Tt-3B  : SGARELINNQEAILNSFKELLTSVLSEGSIDLPINSQIDALLAQKGEDLSLATLATAQLGDAQDDVIDLIVSNVSSQILNAIREHSNEFGFPQETIDQLIYLIVQSYFSEESLQAKIKFS : 554
Tt-3A  : QNQIRDIVDLVLREFKHGLTIKFSESQKEFYDELVKQLSEATENIPEAREQFINNILENEEIYINDLDTISYLRNAIEKRNGSTQTFKEILKILLEYHAKNIKPDPIDEQKSKEEHNSSI : 522
Tt-3C  : TLAHSQFYVQTKKDRLLELVAFAIQKDGKIISRIDKDNLRPNNQNPSLNKYIRYKRNIRDLTKKASDDEAIVVDFSKVPDDVWGFFFFVKLPNLNLLADKLEKENEVLKNSRMGIEDYSN : 711

                730       740       750       760       770       780       790       800       810       820       830       840
Ci     : ------------------------------------------------------------------------------------------------------------------------ :   -
Bf     : ------------------------------------------------------------------------------------------------------------------------ :   -
Sp     : ------------------------------------------------------------------------------------------------------------------------ :   -
Hs     : ------------------------------------------------------------------------------------------------------------------------ :   -
Dr     : ------------------------------------------------------------------------------------------------------------------------ :   -
Xt     : ------------------------------------------------------------------------------------------------------------------------ :   -
Hv     : ------------------------------------------------------------------------------------------------------------------------ :   -
Gp     : ------------------------------------------------------------------------------------------------------------------------ :   -
Vc     : ------------------------------------------------------------------------------------------------------------------------ :   -
Cr     : ------------------------------------------------------------------------------------------------------------------------ :   -
Tt-3B  : PVKPVKGFSQAGIAYLRPDLESLTINTRSPKFGQEPEIGEDEEPIKVVPENLVESEFSNKVSLLNPKDEDLEVLVQHAVAEQVVREDILRVIAGSIKGVDGHEYPILYEALSKTQEYQRK : 674
Tt-3A  : SHQRGSMNDLDDKLYRQKIRILFENTNEYIRNHANVIIRLRVPFLTQQEMEKIRQEAEKQSQEEQKSQNNSQLQQEQEEGSNQVQENQKEQEAQGVQEQLEQGQEQEQEQQPEEEKCKYD : 642
Tt-3C  : NIAIDGVNLMEKIQFDQLIQQPPSEESPTIQNSYYLGYSLTKRPITGWYLENIKIASKGMFEQAEEQEYLNKLVSFWTSTEGIPPKNIEASQVADGEEANVNGSPAQANNLRVMENQSQM : 831

                850       860       870       880       890       900       910       920       930       940       950       960
Ci     : ------------------------------------------------------------------------------------------------------------------------ :   -
Bf     : ------------------------------------------------------------------------------------------------------------------------ :   -
Sp     : ------------------------------------------------------------------------------------------------------------------------ :   -
Hs     : ------------------------------------------------------------------------------------------------------------------------ :   -
Dr     : ------------------------------------------------------------------------------------------------------------------------ :   -
Xt     : ------------------------------------------------------------------------------------------------------------------------ :   -
Hv     : ------------------------------------------------------------------------------------------------------------------------ :   -
Gp     : ------------------------------------------------------------------------------------------------------------------------ :   -
Vc     : ------------------------------------------------------------------------------------------------------------------------ :   -
Cr     : ------------------------------------------------------------------------------------------------------------------------ :   -
Tt-3B  : VLDSIHNDFPVLDMKSY------------------------------------------------------------------------------------------------------- : 691
Tt-3A  : VFVETFDGEGYKRNLIENLNNSQRRPLNEDHPSQINIWVNEERRFLYFDERTLFYLEAVLHKVIRDVLGNKVEPINASQFSQYFDEIQDLVFENFLGDVYEEEKIEKIPIFDIDFGFNLP : 762
Tt-3C  : SLSNGTQFFQKCYKEYKVEFAKNENGELTANGKNYEEFIEELSQYIQSQDAALDALCQWGFEFYFRGKLLKRLQTLKKCKNAFEFEVKKKPEPIVEKVENGEEGEEGEGEEGEEEPEED- : 950

Ci     : -- :   -
Bf     : -- :   -
Sp     : -- :   -
Hs     : -- :   -
Dr     : -- :   -
Xt     : -- :   -
Hv     : -- :   -
Gp     : -- :   -
Vc     : -- :   -
Cr     : -- :   -
Tt-3B  : -- :   -
Tt-3A  : SE : 764
Tt-3C  : -- :   -
```

Supplementary Fig. 1. Domain analyses and multiple alignment of Rsp3 orthologs.
(**A**) Domain organization of *Tetrahymena* Rsp3 paralogs as predicted using SMART (http://smart.embl-heidelberg.de/) [1, 2], showing radial spoke 3 domain (green rectangle) [3] and ARF (ADP-ribosylation factor) domain (red rectangle), uniquely present at the Rsp3C N-termini. (**B**) Multiple alignment of RSP3 orthologs. The amino acid sequences of RSP3 orthologs were obtained from the NCBI protein database (National Center for Biotechnology Information) using Blastp search and human RSPH3 as a bait. The *Tetrahymena* Rsp3 paralogs were obtained from the Tetrahymena Genome Database (https://tet.ciliate.org/). Protein amino acid sequences were aligned using ClustalX2 software [4] and edited using SeaView [5]. The identical and similar amino acid residues were shaded using GeneDoc [6] and the following color code: white letter on a black background (100% conserved residues); white letter on a grey background (80% conservation); black letter on a light grey background (60% conservation).
The color lines indicate the position of the RSP3 domains as follows: red: ARF domain in *T.thermophila RSP3C* (1-164 aa); please note that there is no homology to the N-termini of human RSPH3; green: SMART predicted radial spoke 3 domain (position: 188-470 aa in RSPH3); blue: region corresponding to *Chlamydomonas* RSP3 axoneme targeting domain (ATD) located within amino acids 1-85 aa [7]; purple: AKAP domain in Hs 317-332 aa [8]; a corresponding region in *Chlamydomonas* was named an “amphipathic helix” (AH-R, 161-178 aa) and is marked in pink [9]; AKAP/AH-R anchors PKA (via interaction with RIIa); pink: an amphipathic helix (AH-D, 269-316 aa) binding DPY-30 domain in *Chlamydomonas* RSP3 [9]; green arrow: TQT-like motifs mediating interactions with LC8 [10]; orange arrow: position of the threonine residues, T243 and T286, phosphorylated in RSPH3 [8].

Abbreviations in alphabetical order with the accession numbers.
Branchiostoma floridae (Bf, XP_035686603.1), *Chlamydomonas reinhardtii* (Cr, XP_001695406.1), *Ciona intestinalis* (Ci, NP_001027624.1), *Danio rerio* (Dr, AAI34868.1), *Gonium pectorale* (Gp, KXZ49444.1), *Homo sapiens* (Hs, BAB71615.1), *Hydra vulgaris* (Hv, XP_047128590.1), *Strongylocentrotus purpuratus* (Sp, XP_783727.2), *Tetrahymena thermophila* (Tt, 3A, TTHERM_01044600; 3B, TTHERM_00566810; 3C, TTHERM_00418270), *Volvox carteri f. nagariensis* (Vc, XP_002953748.1), *Xenopus tropicalis* (Xt, NP_998863.1).

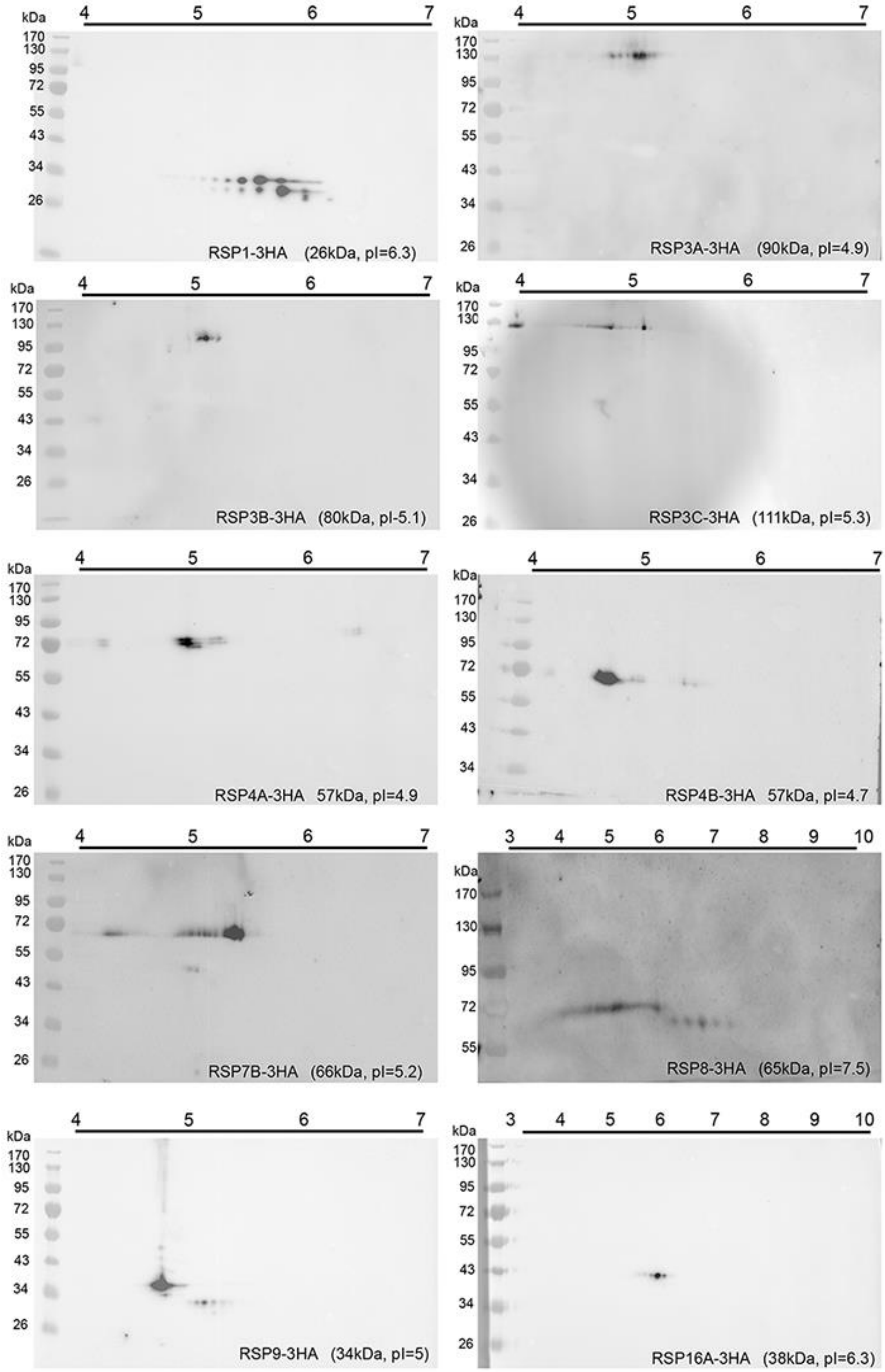

Supplementary Fig. 2. Two-dimensional gel analyses of selected *Tetrahymena* Rsps showing more than one Rsp isoform. The Rsp-3HA fusions were expressed under the control of the transcriptional promoters. The isoelectric focusing of the ciliary proteins (30 μg) was performed using 7 cm 4-7 or 3-10 ready strips. The theoretical pI and Mw values were calculated using https://web.expasy.org/compute_pi/. The Rsp isoforms were detected using anti-HA antibodies. Please note that in *Tetrahymena*, the alternative splicing is a rare phenomenon [11]. Thus, the existence of more than one isoform most likely suggests its posttranslational modification.

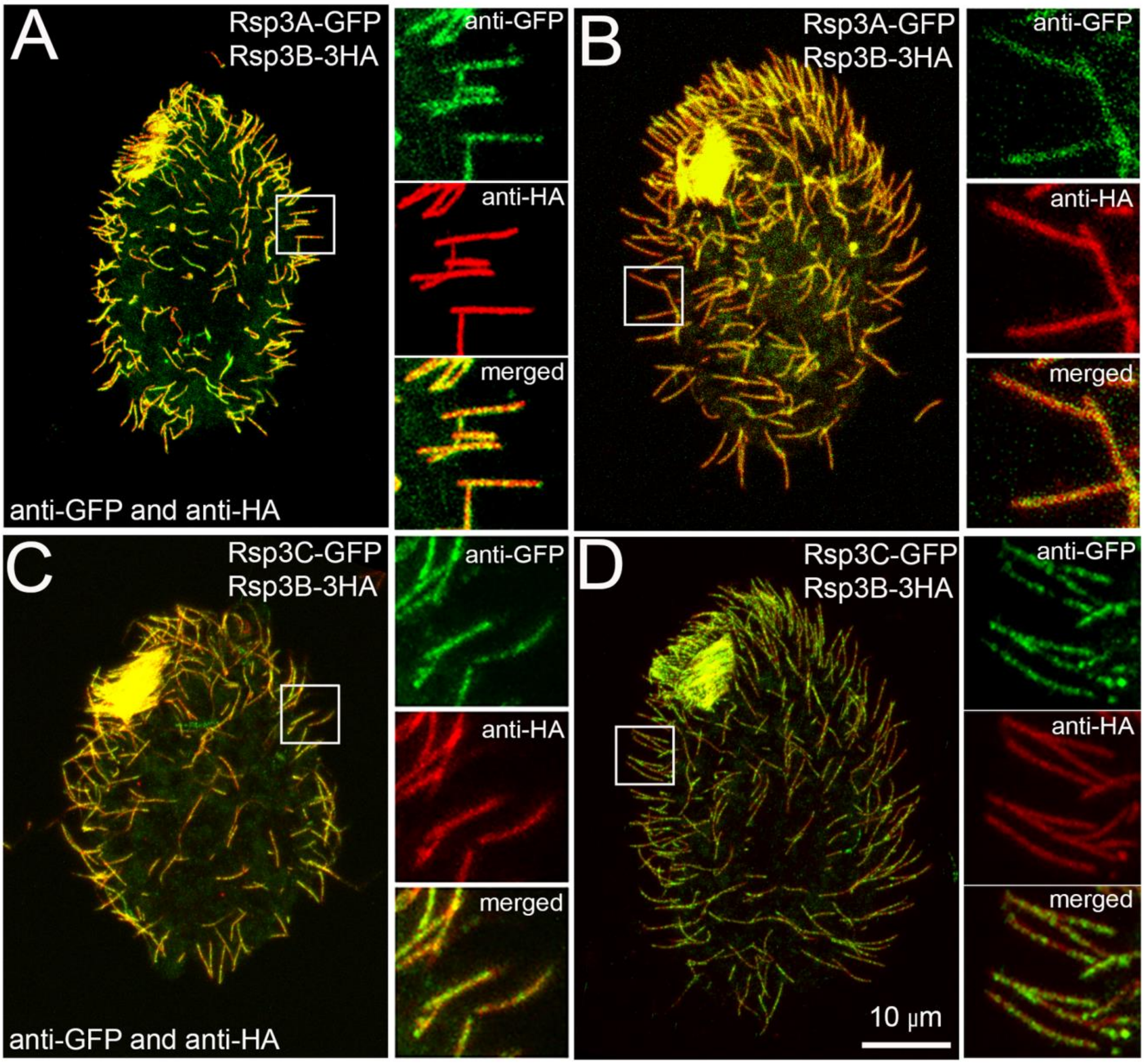

Supplementary Fig. 3. Rsp3 paralogs are simultaneously incorporated into regenerating cilia.

(**A-D**) Confocal immunofluorescence images of *Tetrahymena* cells co-expressing Rsp3B-3HA and either Rsp3A-GFP (**A-B**) or Rsp3C-GFP (**C-D**) under the control of transcriptional promoters, stained with anti-HA and anti-GFP antibodies. Cells were experimentally deciliated and grown in medium for 30 min (**A, C**) or 60 min (**B, D**) to enable cilia regeneration. To the right are magnified areas, marked in the main image by a white frame.

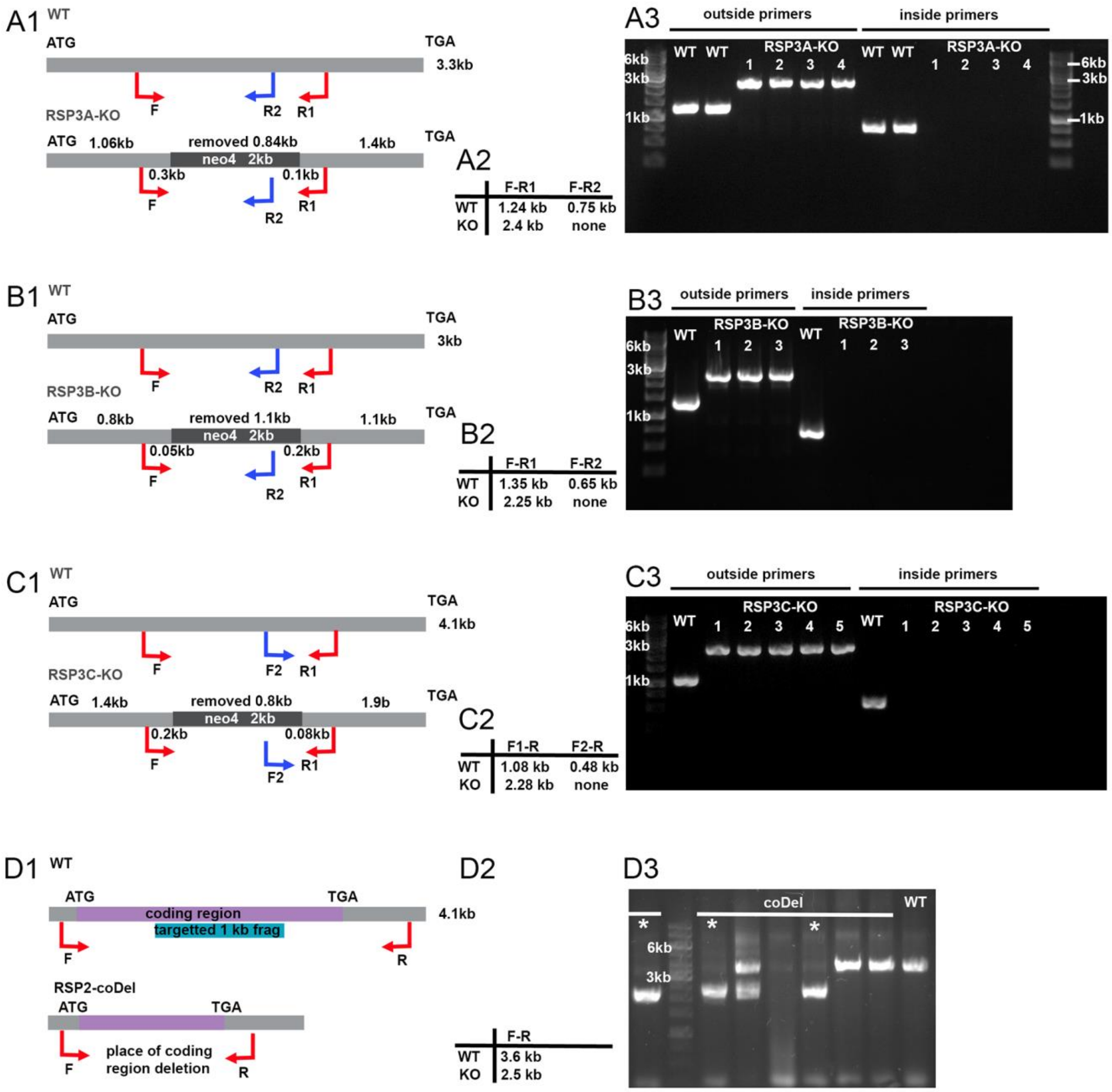

Supplementary Fig. 4. Alterations in *RSP3A*, *RSP3B*, *RSP3C*, and *RSP2* loci in engineered *Tetrahymena* knockout mutants. (**A1, B1, C1, D1**) Schematic representations of the *RSP3A* (**A1**), *RSP3B* (**B1**), *RSP3C* (**C1**), and *RSP2* (**D1**) loci in the wild-type (WT) genome (WT, upper gray bar) and obtained knockout cells (RSP mutants, lower gray bar). The size of a removed fragment of the open reading frame in the knockout cells is indicated. The annealing position of primers (blue and red arrows) used to test changes in the analyzed loci is marked. Numbers near the red arrows indicate the distance between the primer and the neo4 cassette, which replaced the removed fragment of the open reading frame in mutated loci. Note that the primer represented by a blue arrow recognizes the nucleotide sequence deleted in mutant cells and thus anneals only to the genomic DNA from WT cells. Numbers above the gray bars representing the open reading frame indicate the distance between either the start or stop codons and the neo4 cassette insertion site. (**A2, B2, C2, D2**) Table showing the expected size of the PCR products obtained using genomic DNA purified from WT and knockout cells (KO) and primers as indicated. (**A3, B3, C3, D3**) PCR analyses of the RSP3 loci *RSP3A* (**A3**), *RSP3B* (**B3**), and *RSP3C* (**C3**), and *RSP2* (**D3**) loci in independently obtained mutants using, in the case of RSP3 mutants, either both primers annealing outside the neo4 cassette or one of the primers recognizing the deleted gene fragment as indicated in A1, B1, and C1, respectively. (**D3**) A star indicates clones with *RSP2* gene deletion (RSP2-coDel mutants).

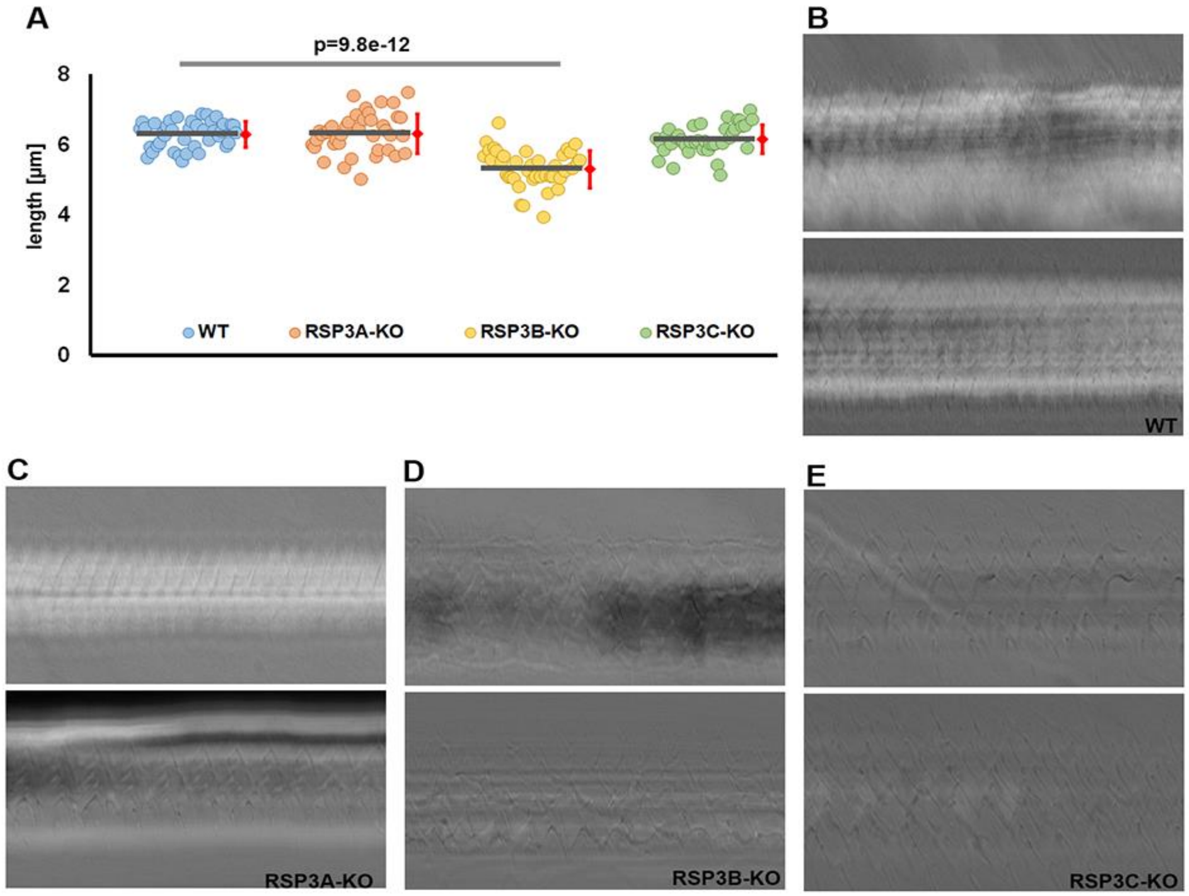

Supplementary Fig. S5. Deletion of a single *RSP3* gene had no effect on *Tetrahymena* cilia length but affected cilia beating. (**A**) Graphical representation of cilia length measurements. WT and *RSP3* knockout cells were double labeled with anti-acetylated tubulin and polyG (anti-polyglycylated tubulin) antibodies, and cilia length was measured in confocal microscopy images using the ImageJ program (N=40 cilia). Red bars represent standard deviation. Note that deletion of *RSP3B* slightly but significantly affects cilia length (t-test). (**B-E**) Examples of the kymographs showing cilia beating. Swimming cells were recorded using a high-speed camera and a 40x objective and analyzed using ImageJ. Note that only knockout of *RSP3B* causes apparent cilia asynchrony.

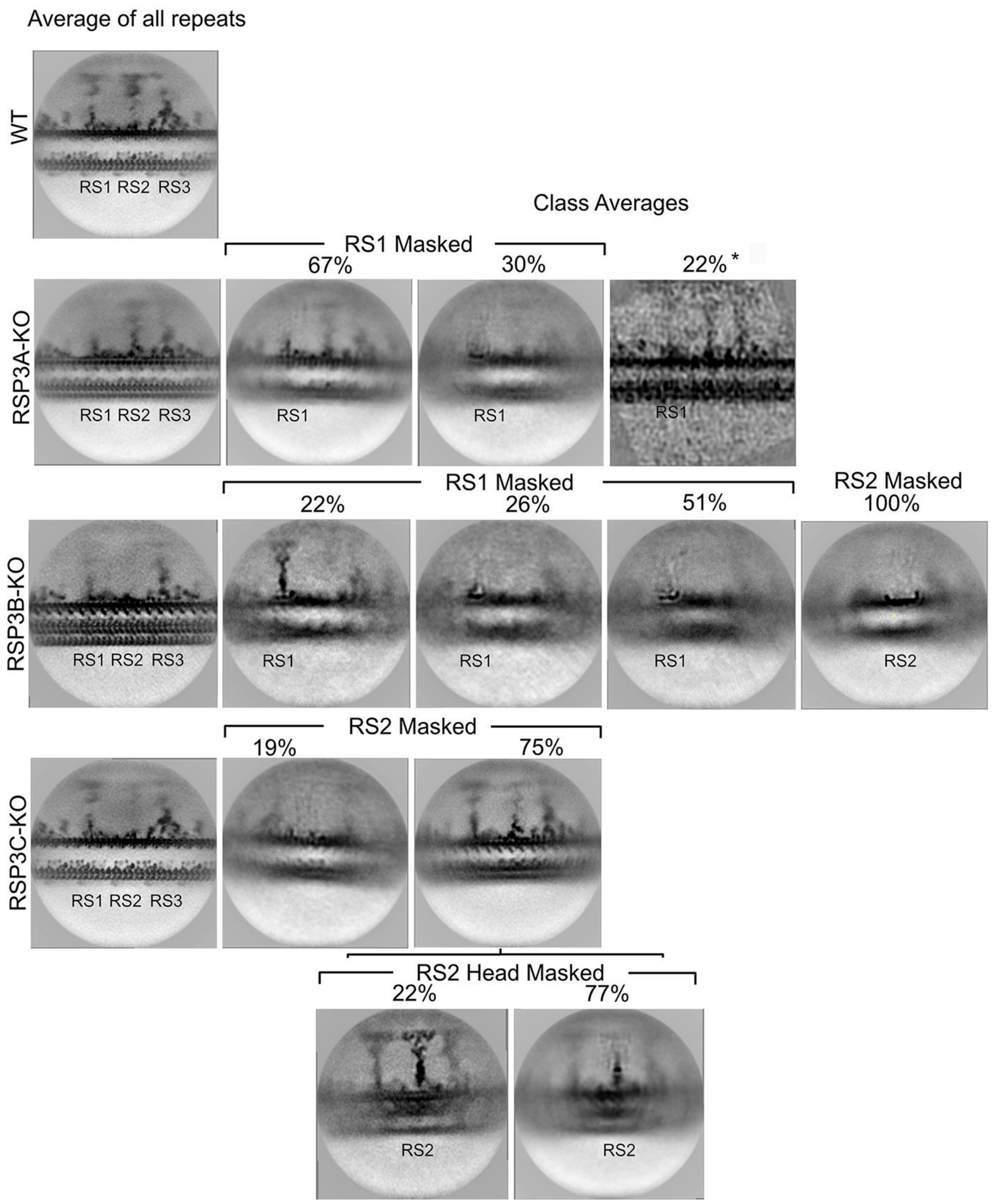

Supplementary Fig. 6. Consensus subtomogram averages and three-dimensional classification of the RSs in *Tetrahymena RSP3* knockout mutants. The 3D classification in Relion of n=2099 axonemal repeats of RSP3A-KO revealed either the lack of the entire RS1 (30%, n=639 units) or RS1 except for the RS1 base (67%, n=1417). Three percent of the recorded repeats were not classified due to the low number of particles. A 3D classification for intact RS1 in Relion was unsuccessful; therefore, we performed manual classification using visual inspection in ArtiaX. Subtomogram coordinates were mapped onto the denoised tomogram, and those containing intact RS1 were manually selected in ArtiaX. Due to the limitation of the missing wedge, we could only inspect two doublets showing RS1 in the side view of each tomogram. On average, each doublet contained three intact particles, leading to an estimated 13 intact particles across all nine doublets. With 35 tomograms analyzed, the dataset contained approximately 455 out of 2090 intact particles (~22%). The selected subtomograms were then rotated based on a reference and averaged using a Python script. In RSP3B-KO mutant cilia, all analyzed axonemal repeats (n=2092) lacked RS2. Additionally, 77% of the axonemal repeats (n=1622) had RS1 spoke defects. The RS1 spokes were either completely missing (51%, n=1078) or only their base was clearly visible (26%, n=544). We collected n=2790 axonemal repeats of RSP3C-KO mutant cilia. Among them, 173 axonemal repeats (6%) were unclassified, and ~19% (n=527) lacked the entire RS2. The 3D classification of the remaining repeats using the RS2 mask covering an RS2 head and stalk showed two RS2 categories: (i) intact RS2 structure (~22%, n=469) and (ii) only well-visible RS2 base (~77%, n=1621). To summarize, among classifiable RSP3C-KO axonemal repeats, 19% lacked the entire RS2, 58% showed only the RS2 base, while in 17% of repeats the RS2 was intact.

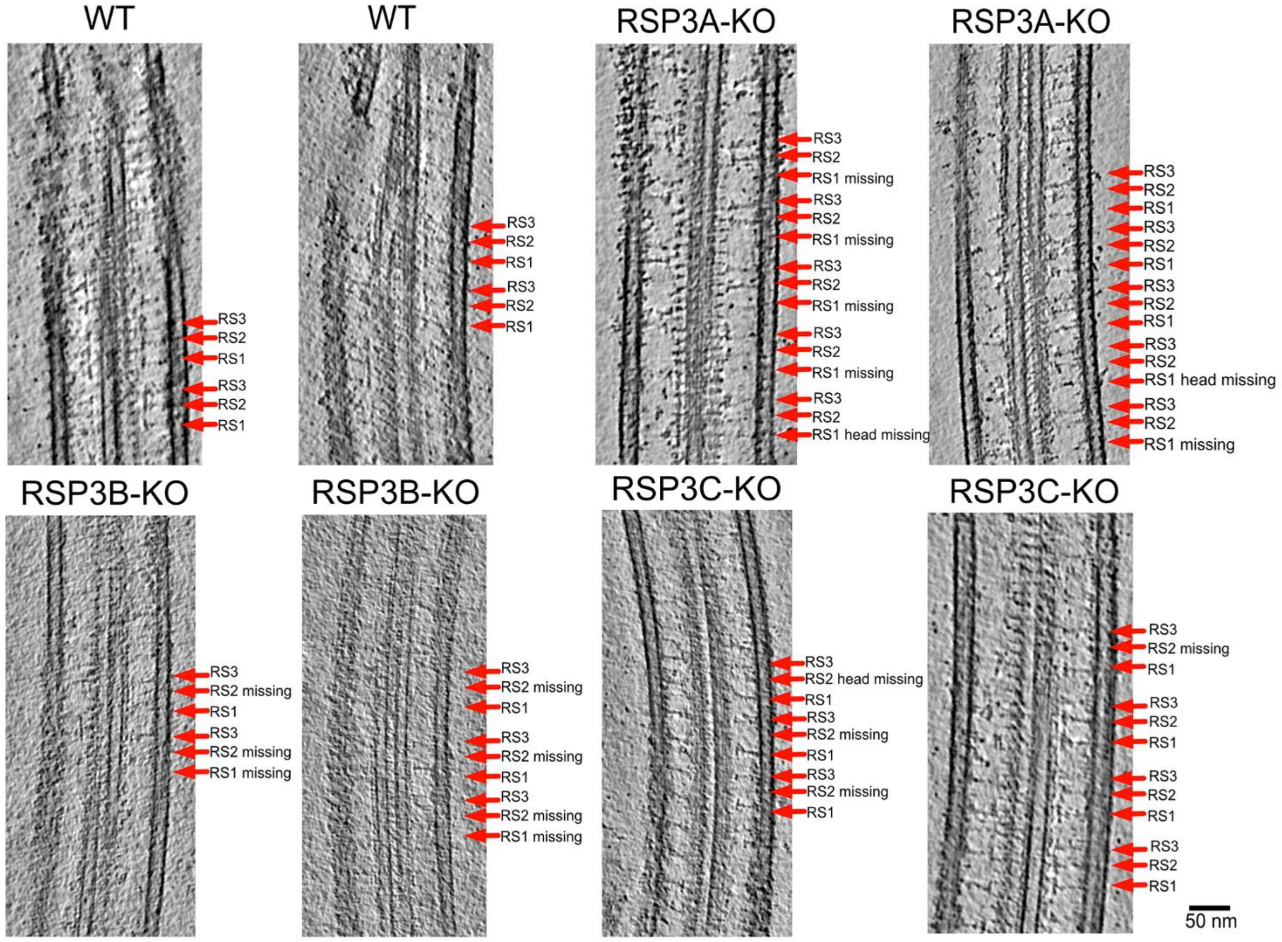

Supplementary Fig. 7. Tomographic slices from WT, RSP3A-KO, RSP3B-KO, and RSP3C-KO cilia showing the heterogeneity of distributions of RS with structural defects. Scale bar: 50 nm.

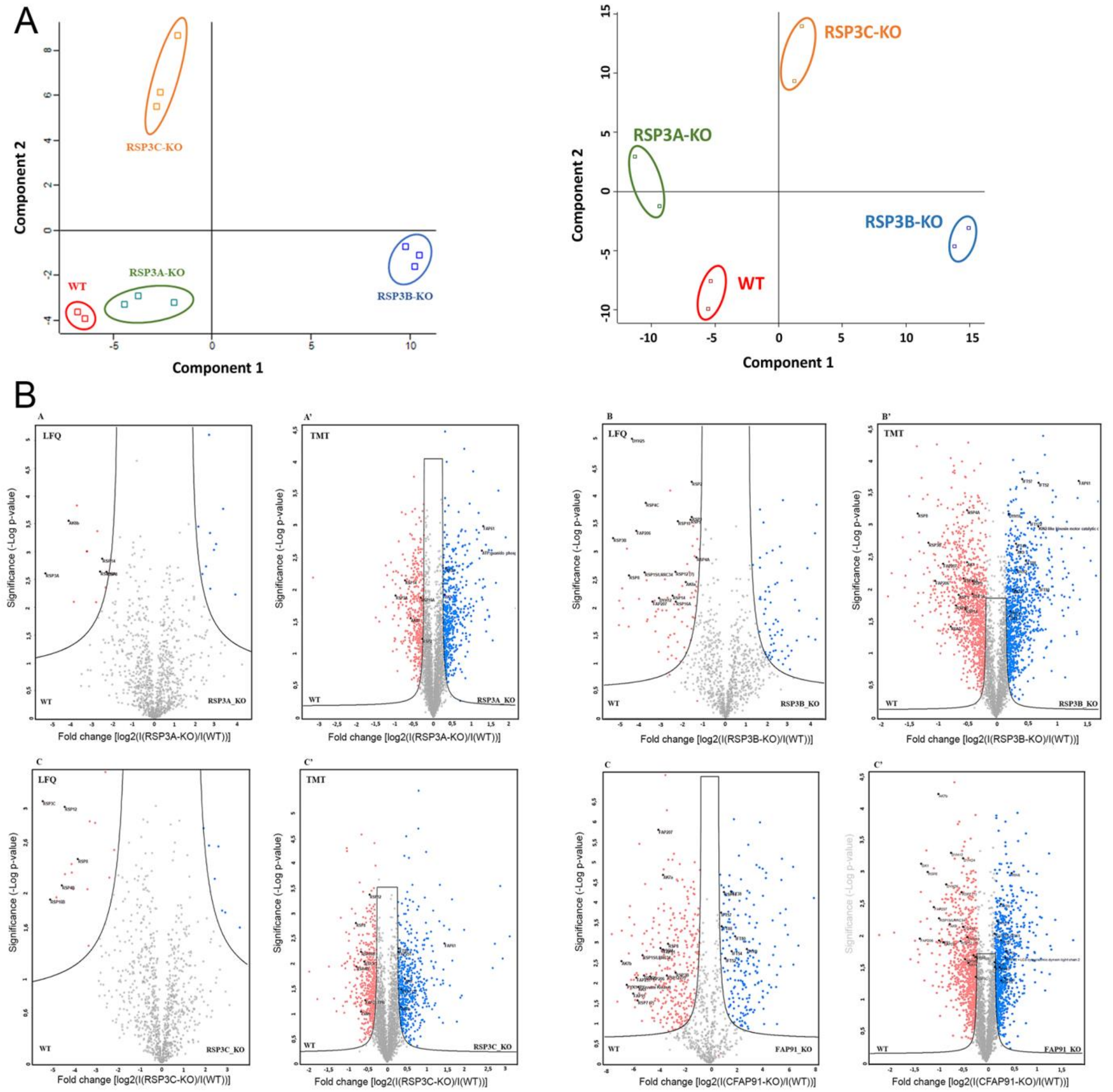

Supplementary Fig. 8. Graphical illustration of quantitative proteomics data. (**A**) Principal component analysis (PCA) score plot from fold-change values of the differentially expressed proteins showing similar grouping of ciliomes identified by mass spectrometry using either the LFQ (graphs to the left) or TMT (graphs to the right) strategy based on two independent variables. The analyzed ciliary protein samples were color-coded as follows: WT (red), RSP3A-KO (green), RSP3B-KO (blue), and RSP3C-KO (orange). (**B**) Volcano plots of WT and RS mutant ciliary proteins identified by mass spectrometry using either the LFQ (graphs to the left) or TMT (graphs to the right) strategy (N=3) showing changes in mutant ciliomes. FDR=0.05. Red dots represent proteins that are at a significantly lower level in RSP mutants compared to WT cells, while blue dots represent proteins that are more abundant in mutant cilia. The statistical significance of the difference in protein enrichment between WT and RS mutant ciliomes was calculated using Student's t-test.

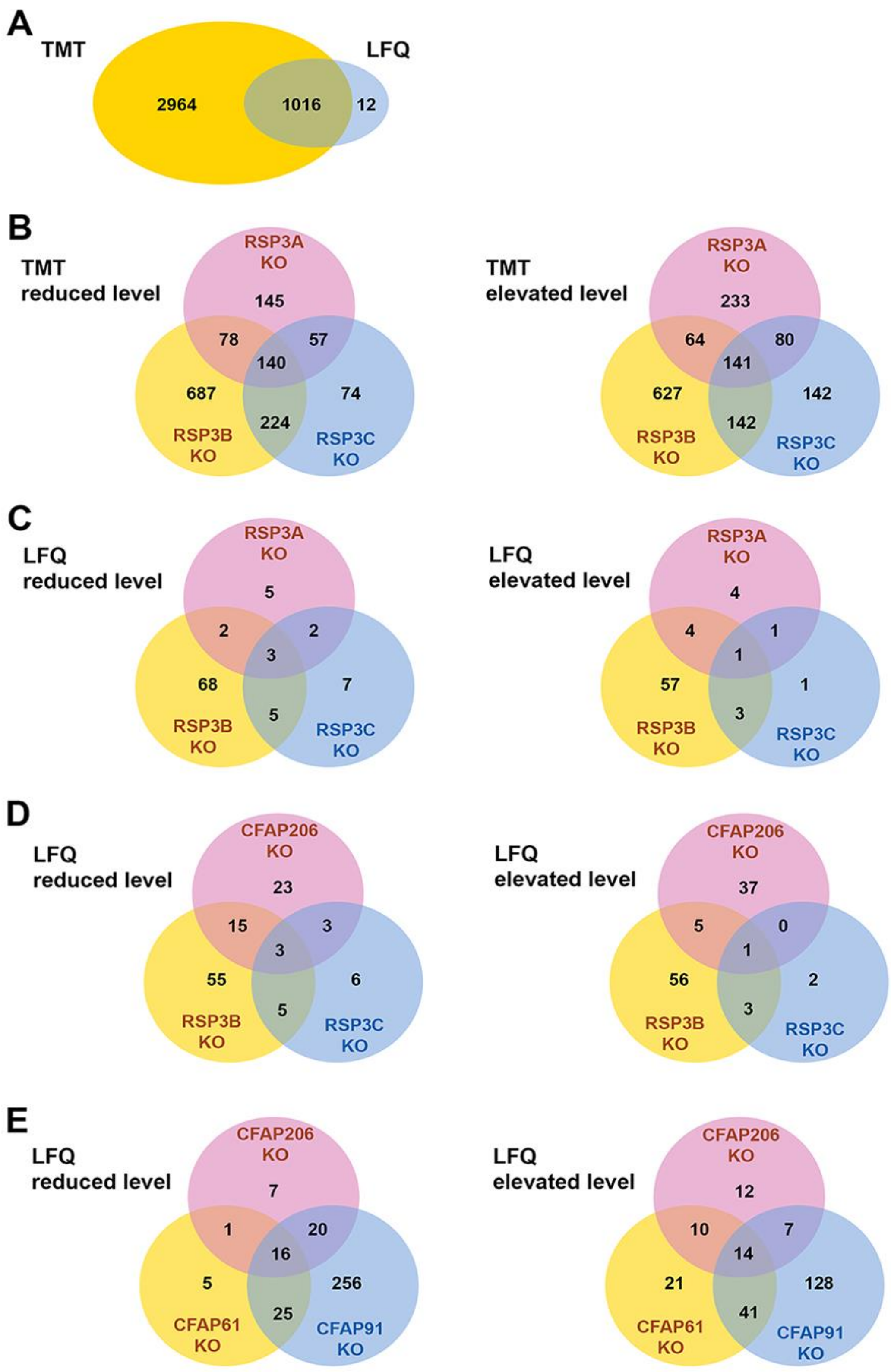

Supplementary Fig. 9. Venn diagrams indicating the overlap of differentially expressed proteins between different RS mutants based on ciliomes obtained either by LFQ or TMT approaches. (**A**) Comparison of the total number of proteins identified using LFQ and TMT approaches. (**B-E**) The overlap of proteins that were either reduced (to the left) or elevated (to the right) in different RS mutants as detected using (**B**) TMT and (**C-E**) LFQ methods. Images were prepared in Adobe Photoshop based on data sorted using the Microsoft Excel program.

RSP1 and RSP10

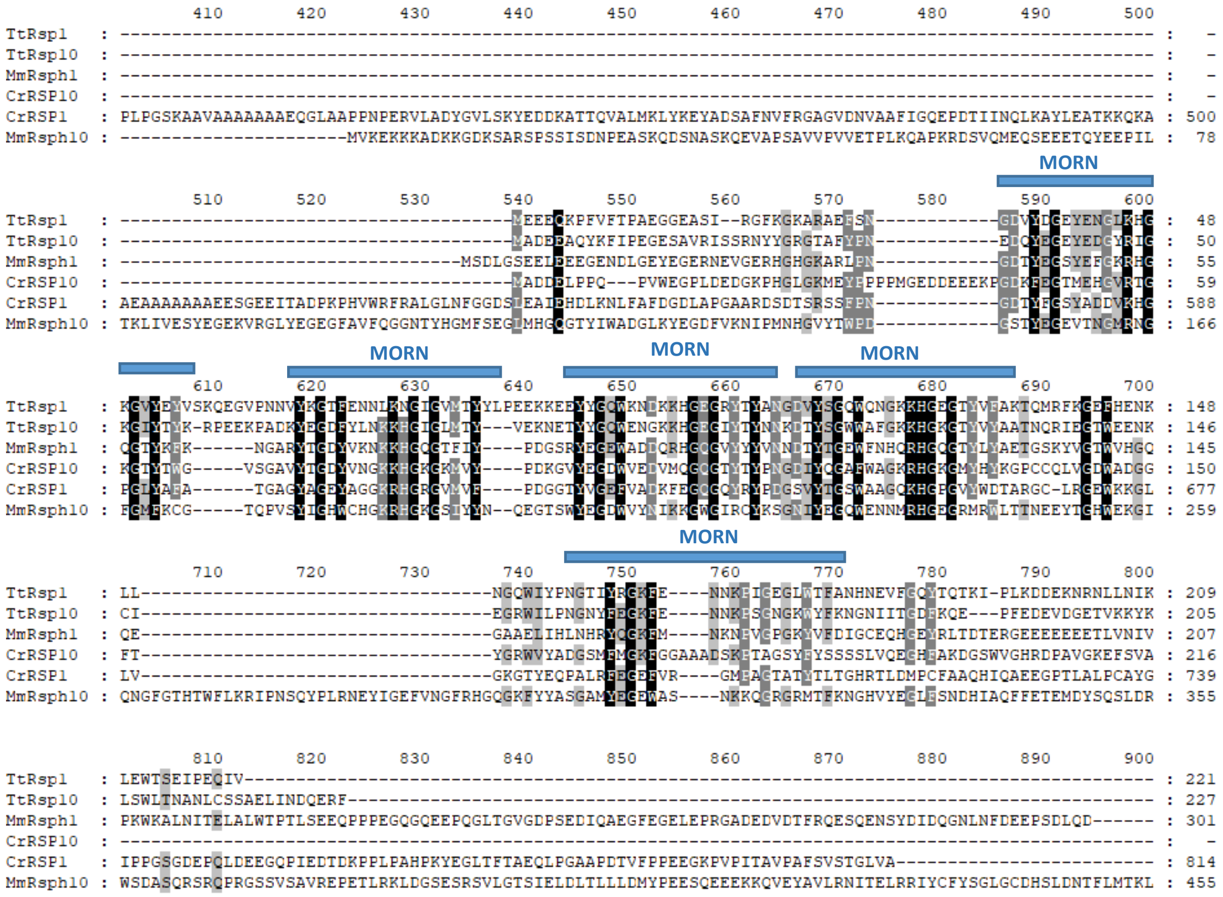

The following fragments: CrRSP1 M1-A400 and MmRsph10 H456-K876 are not shown.

Domain position in *Tetrahymena* orthologs.

**TtRsp1**	**domain**	**aa position**	**e-value**
	MORN	35-56	0.00000506
	MORN	64-85	0.172
	MORN	91-112	0.00000236
	MORN	114-135	1.95e-7
	MORN	160-181	2.35

**TtRsp10**	**domain**	**aa position**	**e-value**
	MORN	37-58	0.108
	MORN	65-86	0.0547
	MORN	89-110	0.0000134
	MORN	112-133	0.00298
	MORN	158-179	3.1

RSP2

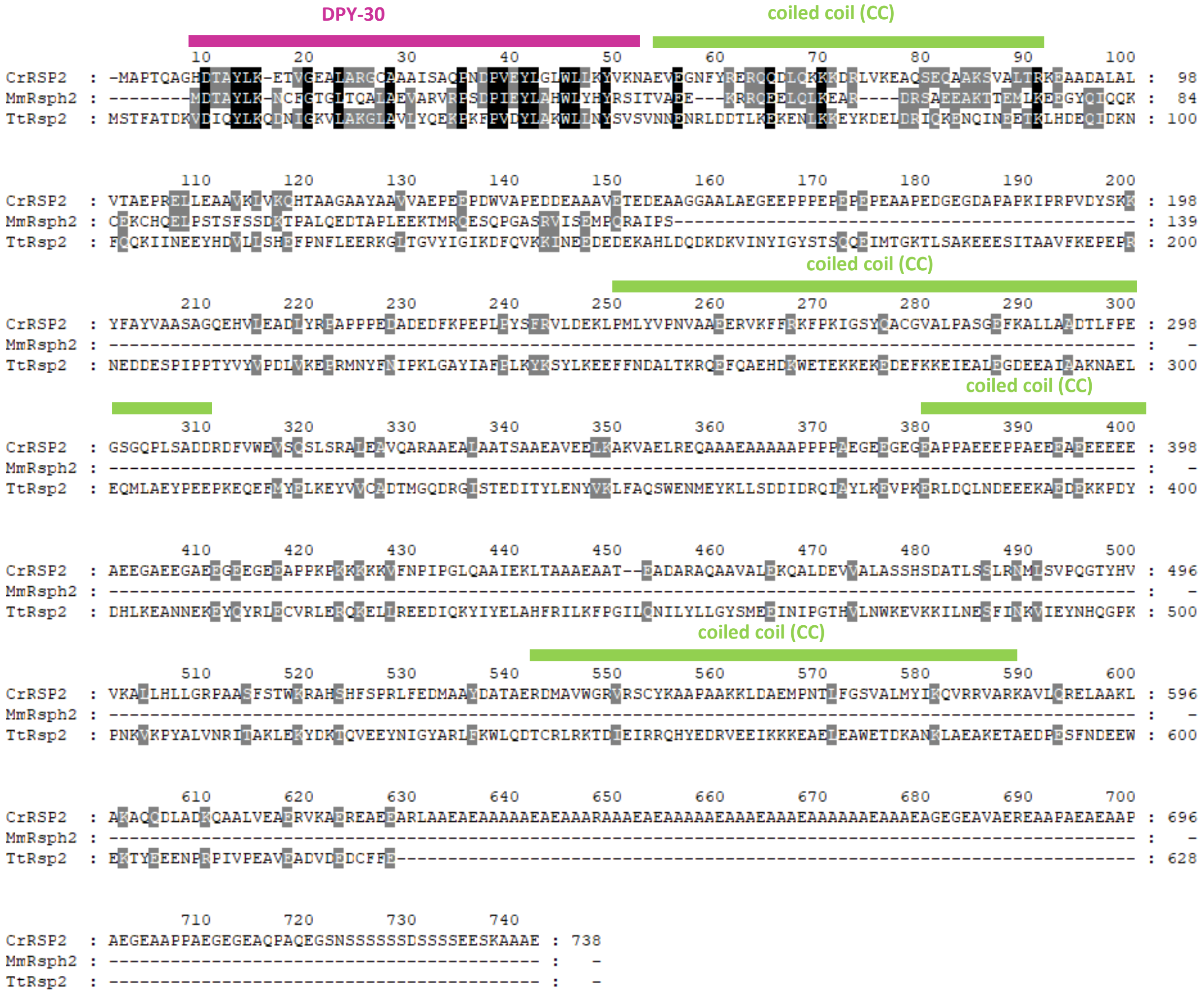

TtRsp2	domain	aa position	e-value
	DPY-30	10-51	9.6e-9
	CC	52-92	n/a
	CC	251-310	n/a
	CC	379-399	n/a
	CC	544-591	n/a

RSP4/6

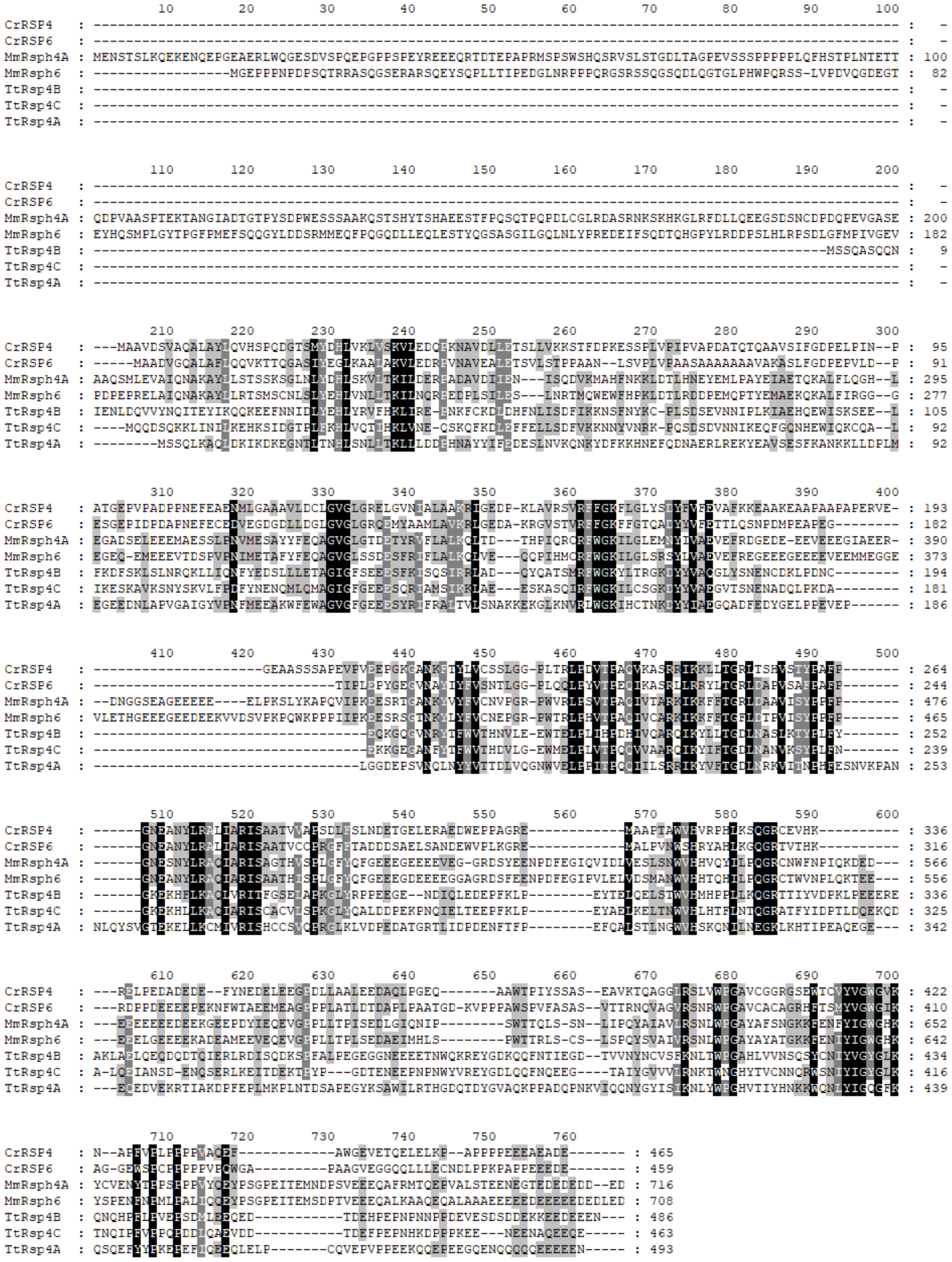

RSP7

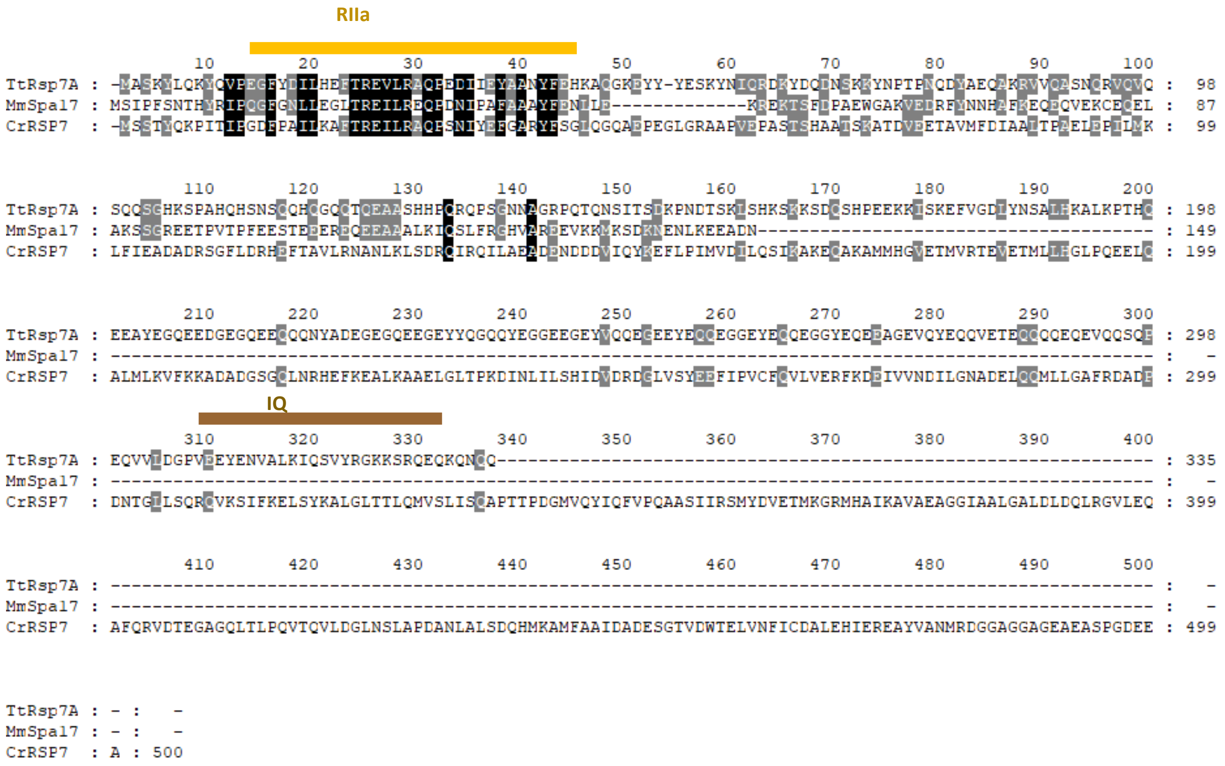

**TtRsp7A**	**domain**	**aa position**	**e-value**
	RIIa	13-45	1.4e-11
	IQ	309-331	6.06

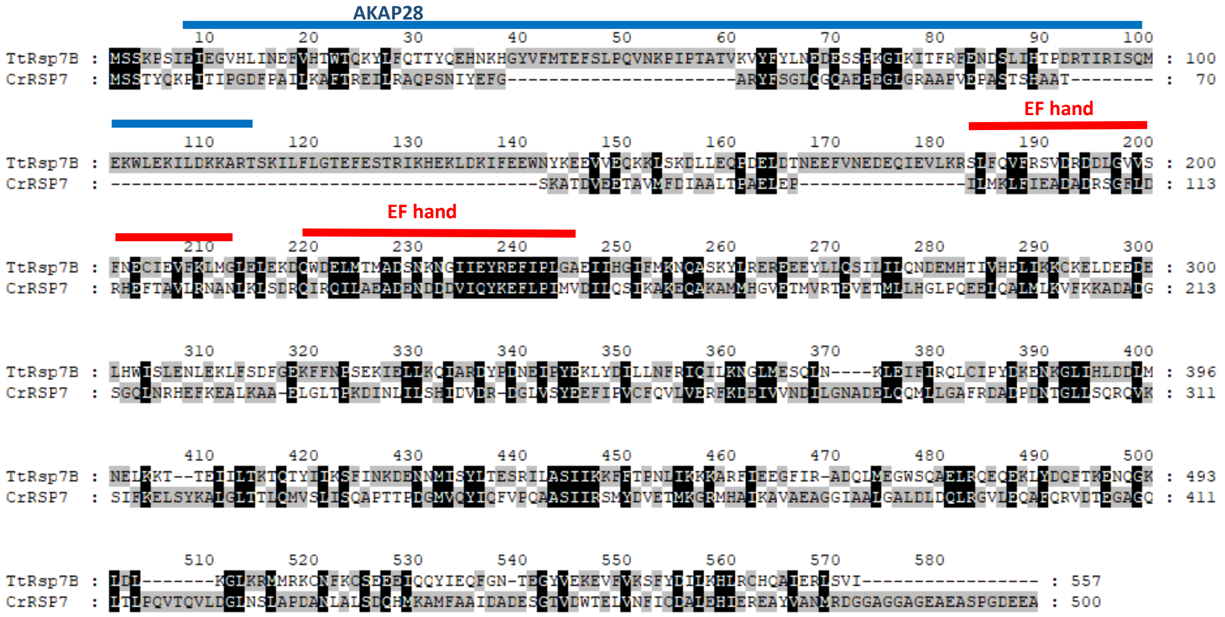

**TtRsp7B**	**domain**	**aa position**	**e-value**
	AKAP28	8-115	5.9e-19
	EF hand	183-211	0.65
	EF hand	219-247	0.614

RSP8

Armadillo-type

```
                   10        20        30        40        50        60        70        80        90       100
TtRsp8 : MQSYK-LHVVSEHLQDRYFYLEADFNDRYDNTEVYHAVFDRSIPKLIEILLMPEIEEFKYRDALITLNEMVSHQEMKDQMISQGLIS-IASAFLHHKIVE :  98
CrRSP8 : MQSHSSRHVVSEHLKDLHPDLSEPFPKHVIEDEVAEAHGRRAIPKLVAVLALPELPDDQRAHALRVLNGLLSTQEHKTNAVAEGAAPPLCQLASQCRDDE : 100

                  110       120       130       140       150       160       170       180       190       200
TtRsp8 : IRREAVLLLGSLVSIMRGRQIVDDLTYEGFKNLLFDENLKAREANAWALCRFLTGRDGVDRLAQSKIIQYMVESFLKYTEQPKIEEAKFIIMLLEGFLNI : 198
CrRSP8 : VRRLSCSALASLGQVMAGRNGIVAAGGLPVLTEALQTTPEQAAAALKSFAASNDGAAQLNLERAAIVPALVTLLSQPTDPAFTLTAFSNALSTLEGMTRT : 200

                  210       220       230       240       250       260       270       280       290       300
TtRsp8 : LQYDNGIVFFVKTGIVARLNKILRNEDDTYYDRQWSLRINYLCLDCLAKICVNTQGKEEGIELKVINTANNFLDSPINEEKKYSVVLIMNCSIHLDGKKQ : 298
CrRSP8 : DDGVLAALDGGVPACLVALARRGLEGDLKFEGRLMELLQLVATCLEQICHHADGKAACRQAEAHKVLAELLTLQHREIIKHAAAALMGLAVEKESKVNVM : 300

                  310       320       330       340       350       360       370       380       390       400
TtRsp8 : CTYVDDDNLIRKLIALLDSKDENLRVDVKQALKNIAELPDGFLVITAKLAHNLNYLKEVFFVKQGNHRVFEQPVLLALAKLLPKTSELQNPENLPANKVL : 398
CrRSP8 : LYAGVSLVRLMRGSDAELAANARDTVAAAAEHLEARRTAEMLLSMEERELLLWRG----------------------------------PPPETPP---- : 362

                  410       420       430       440       450       460       470       480       490       500
TtRsp8 : DYEKYCIAICYFILNQEEVIHDALEQIVKLVEKLAPFLLFKNNKQLQDMVAHTLQKLIQDSETKRQLIQYIMKYGNVAHQSNNSTLNEEIAKYKKLSEML : 498
CrRSP8 : DYRYHVDLPKFTPQAK------------------------------------------------------------------------------------ : 378

TtRsp8 : SK : 500
CrRSP8 : -- :   -
```

TtRsp8	domain	aa position	e-value
	Armadillo-type	42-468	n/a

RSP9

```
                    10        20        30        40        50        60        70        80        90       100
TtRsp9  : --MDIYRLSSDIKYVNQIGVTLNVEEKLKLELSLLQITETEKFDNILFWGKINGVKFDYYIAVG-----LNFKGKFEFPTKKFYWAKSDTFVFDELPEIN :  93
MmRsph9 : --MDADSLLLSLELASGSGQGLSPDRRASLLTSLMLVKRDYRFARVLFWGRILGLVADYYIAQG-----LSED--QLAPRKTLYSLN--CTEWSLLPPAT :  89
CrRSP9  : MVQLEPNITLVLKHLASCGAVVSAEQQAALDHSIPIKRIEAGLRSLTLWGRLTTLNGKDYLVAEGYNVASSKEGAAVYETKYFYSQD--GARWSDLQPVD :  98

                   110       120       130       140       150       160       170       180       190       200
TtRsp9  : LEYRAQVDSFRNLFTGDHKFVLIPLPEDQQAEECNQEQAEGEEKPEDSDEEENIKVKPKAFTELDRLAYVVRAIENDCALVEVGAFKLTETHELRYNDSF : 193
MmRsph9 : EEMAMQISVVSGRFMGD--------PSHEYEHTELQKVNEGEK---VFDEEVVVQIK-----EETRIVSIIDQIDKAVAIIPRGALFKTEFGVTHVNRTF : 173
CrRSP9  : SETATRCARIKGMLSGD--------PAKNYELEEKDPNAPEPS----PEAEEEVKPLVFQIPELAVIRCRVDAIATATSVIETDSTIINAASQVVPNRLF : 186

                   210       220       230       240       250       260       270       280       290       300
TtRsp9  : RGASLDSVANINTYQHFRAPQSEEKKKFIARDIALFHFNFLDPIEHDLPKGCWSVQIISSKTSATVRSLLWPGFVGYHRANSQIFGYAYIGNGIKNSDLP : 293
MmRsph9 : EGLPLSEVRKLSSYFHFREAIDLKNKTLLEKSDLEPSLDFLDSLEYDIPRGSWSIQMERGNAIVVLRSLLWPGLTFYHAPRTKNYGYIYVGTGEKNMDLP : 273
CrRSP9  : AGAAYP--EKLESYQHR-----------------FSLPGSGVTLSQDLRGTWAVCYIAFKGVACVRSLLFPGYFFYYAANELTWGSLYVGDGLRNNDLI : 266

TtRsp9  : FLL : 296
MmRsph9 : FML : 276
CrRSP9  : FML : 269
```

RSP11

RIIa

```
                     10        20        30        40        50        60        70        80        90       100
TtRsp11  : MADVREQRIYCAEQIVVPQELPIILKHYSKEVIRNNPVSIYDFSAKYFRDLIE-----------KRK--NNSDKHIMNELEKPDSSSRV----------- :  76
CrRSP11  : -MDVEP---IFCAEQIVIPHNLADILKAYTKEVIRRQPTDLIAFSAKYFTNLAN-----------VASGVSNSSAPAKEQLRQVYTRGGSGGATLTESQVT :  86
MmRsph11 : --MPLPDTMFCAQQIHIPPELPDILKQFTKAAIRTQPADVIQWSAGYFSALSRGDPLPVKDRIEMPVATQKTDTGLTQGLLKVLHKQCSHKQYVELADLE :  98

                    110       120       130       140       150       160       170       180       190       200
TtRsp11  : ---------------------------------------------------------------------------------------------------- :   -
CrRSP11  : GLCQQAGIADAVVAKVMEVGAFTPAAVDLSKFVFLCLAMS---CEDFNRVCMGVFDVFSDNGSLPAQDLLTLIAHLGPDMDPEVTPAFLDAVAAELPAGG : 183
MmRsph11 : KKWKNLCLPVEKLRTILELDPCEDKIEWIKFLALGCSSLGRTLNTAMKNVCEILTSDPEGGPARIPFETFAYVYQYLSGLDPELPAVETENYLTSLRLMS : 198

                    210       220
TtRsp11  : --------------------- :   -
CrRSP11  : GAVTYMELCEAPSLKPKLGLS : 204
MmRsph11 : ESRKNGMIGLSDFFVGKKII- : 218
```

TtRsp11	domain	aa position	e-value
	RIIa	21-56	2e-7

RSP12

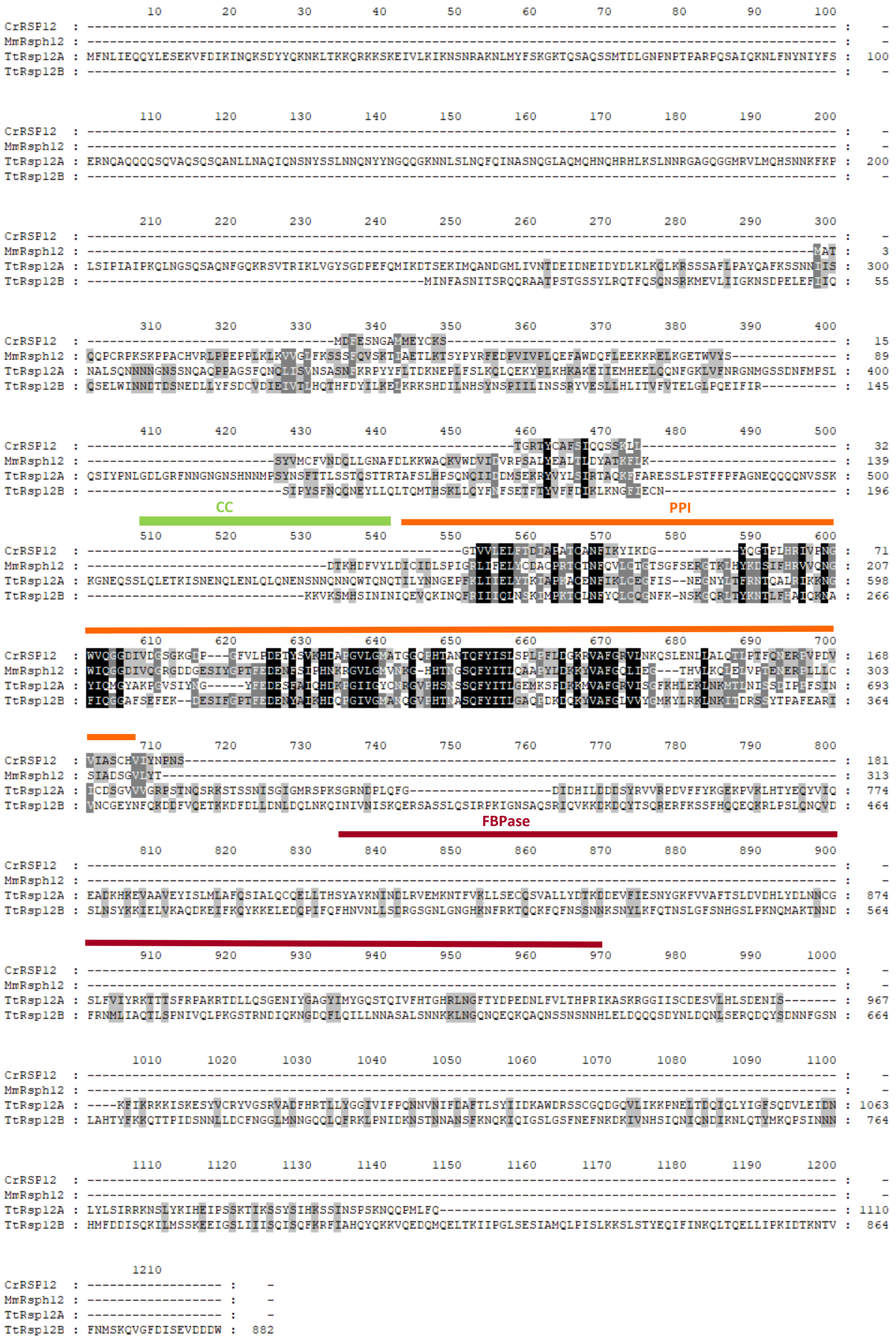

	domain	aa position	e-value	
**TtRsp12A**	PPI	544-697	1.4e-27	
	FBPase	809-941	3.8e-18	
	CC	509-542	n/a	
	**TtRsp12B**	PPI	209-369	6.3e-35

RSP14

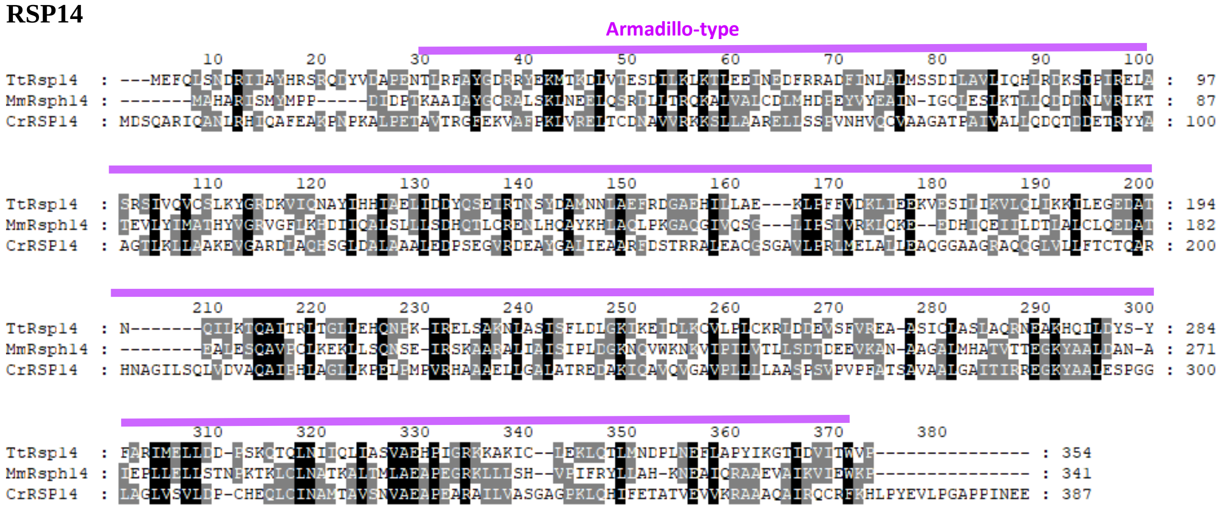

TtRsp14	domain	aa position	e-value
	Armadillo-type	26-347	n/a

RSP15

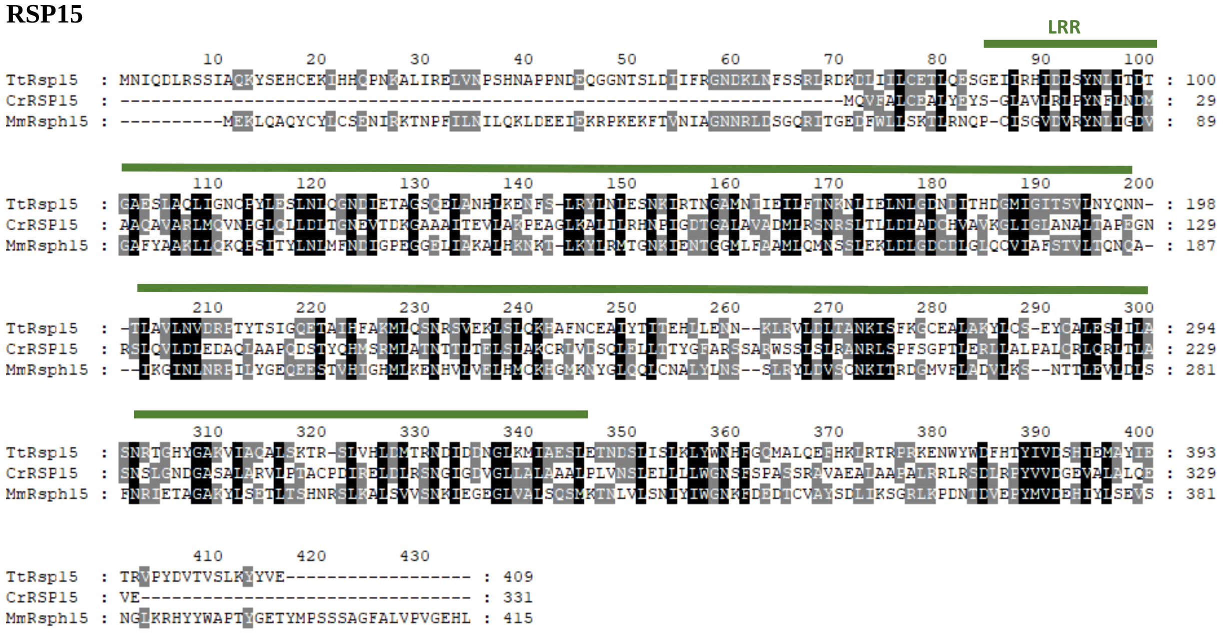

TtRsp15	domain	aa position	e-value
	LRR	84-111	5.96
	LRR	112-139	1.73
	LRR	140-167	6.57
	LRR	168-195	1.78
	LRR	228-255	112
	LRR	256-283	1.33
	LRR	285-312	3.1
	LRR	313-340	5.06

RSP16

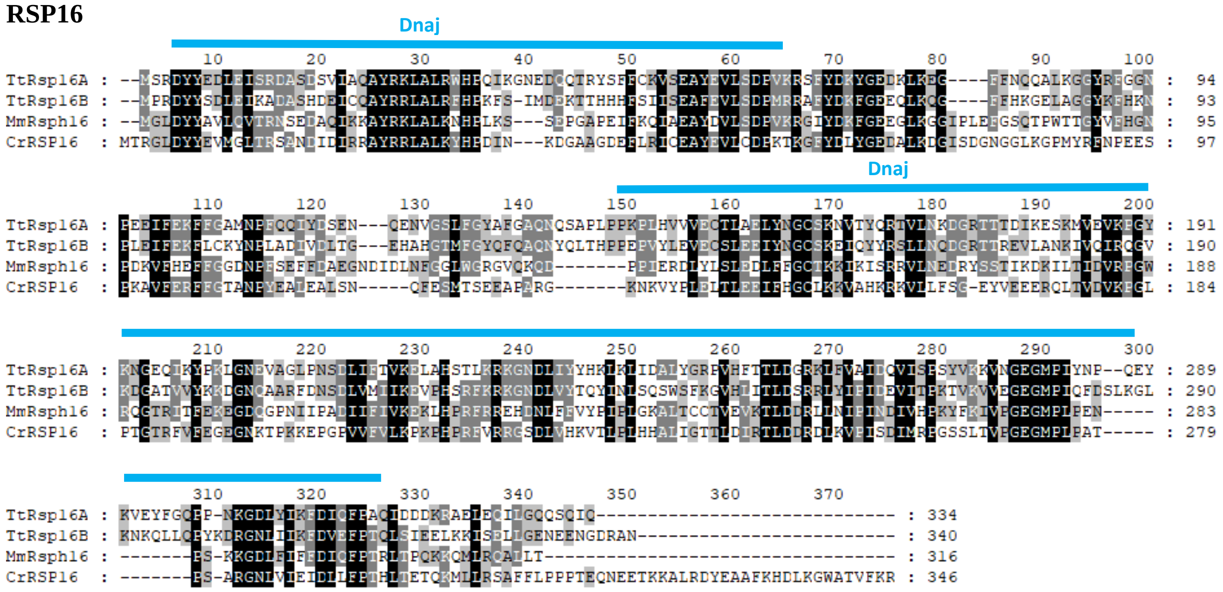

	domain	aa position	e-value
**TtRsp16A**	Hsp40/Dnaj	3-63	1.16e-14
	Hsp40/Dnaj	143-312	1.8e-36
**TtRsp16B**	Hsp40/Dnaj	3-62	1.31e-8
	Hsp40/Dnaj	142-314	1e-31

RSP20

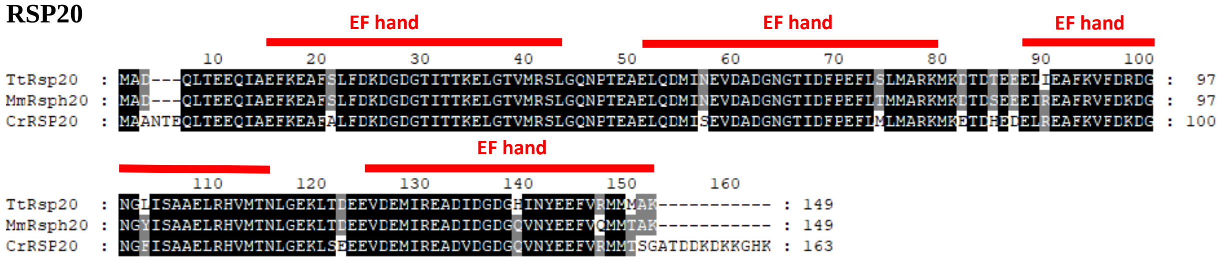

**TtRsp20**	domain	aa position	e-value
	EF hand	12-40	6.39e-9
	EF hand	48-76	2.39e-8
	EF hand	85-113	5.04e-7
	EF hand	121-149	3.67e-9

RSP22

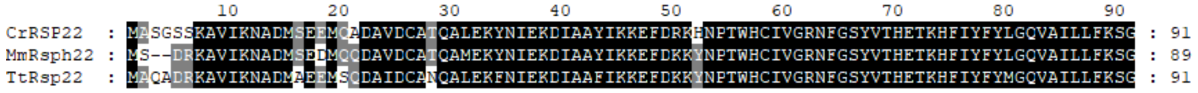

RSP23

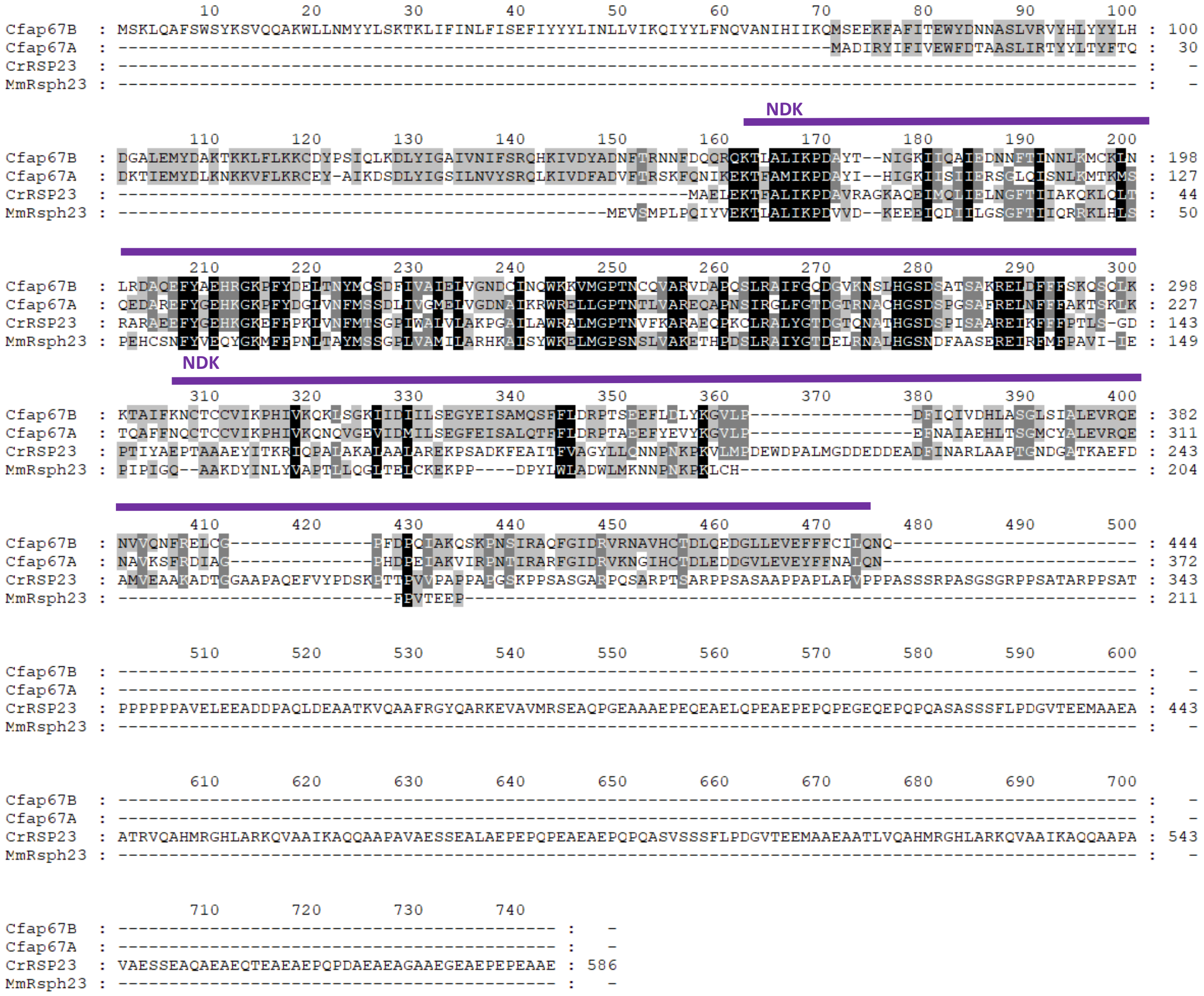

TtRsp23	domain	aa position	e-value
	NDK	89-227	4.94e-68
	NDK	233-372	1.56e-38

FAP198

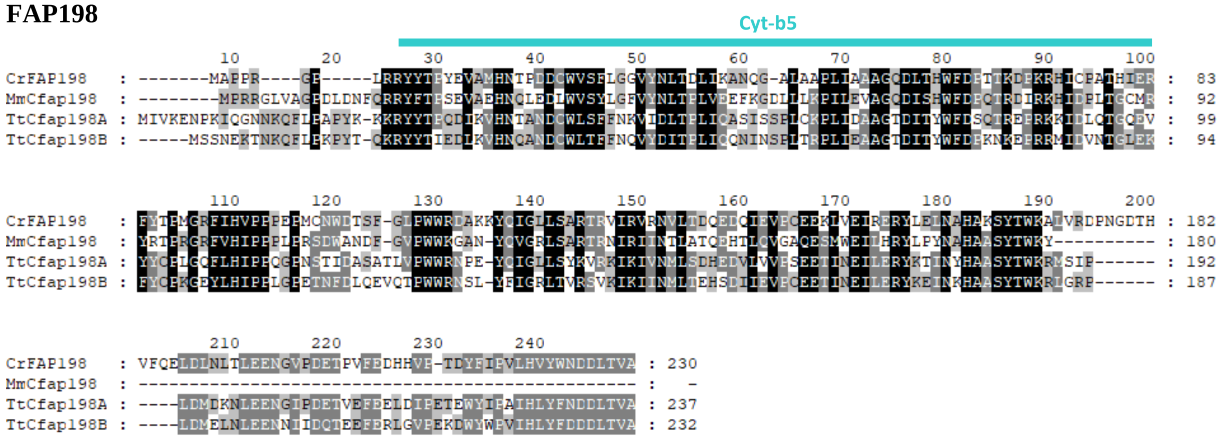

	domain	aa position	e-value
**TtCfap198A**	**Cyt-b5**	27-99	0.0288
**TtCfap198B**	**Cyt-b5**	22-103	0.0102

FAP207

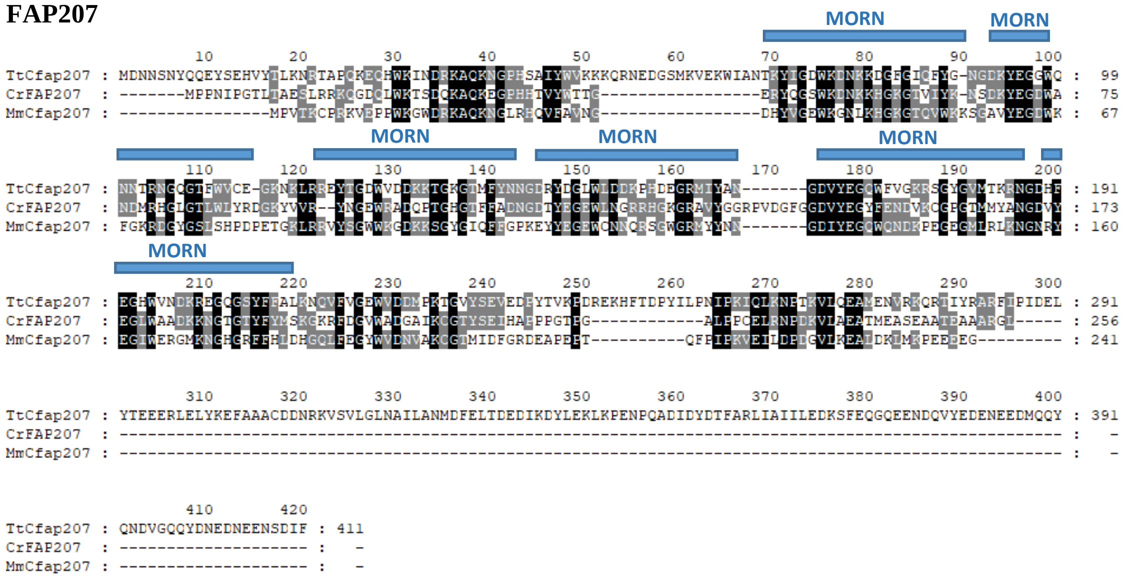

TtCfap207	domain	aa position	e-value
	MORN	69-90	0.0314
	MORN	92-113	0.148
	MORN	120-141	0.0274
	MORN	143-164	0.0293
	MORN	166-187	0.0000405
	MORN	189-210	0.22

FAP253

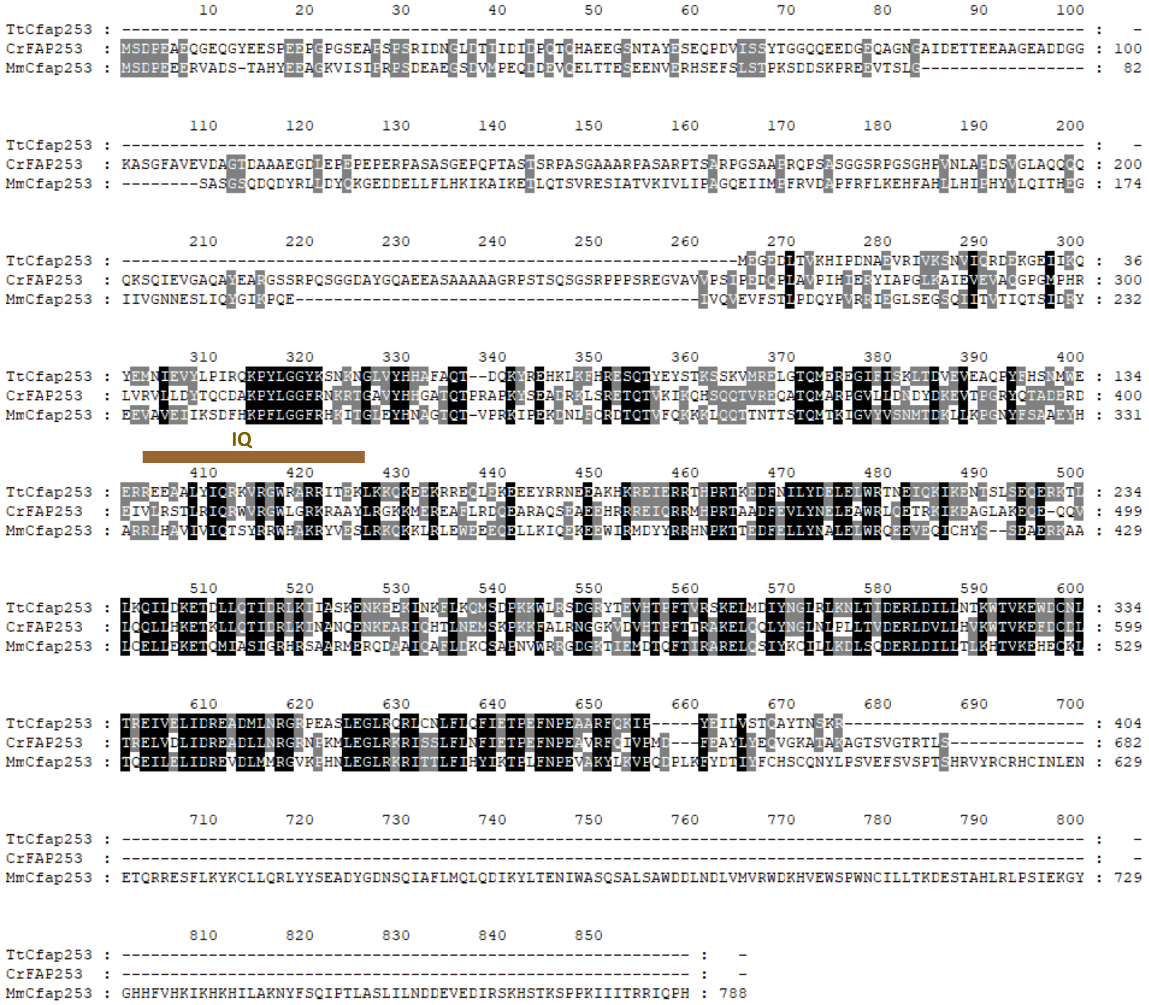

TtCfap253	domain	aa position	e-value
	IQ	136-158	1.3

Supplementary Fig. 10. Multiple alignments and domain analyses of RSP orthologs. Domain organization is predicted using SMART (http://smart.embl-heidelberg.de/) [1, 2] or UniProt (htttps://www.uniprot.org) [12]. Domains are marked according to position in *Tetrahymena* proteins. The amino acid sequences of RSP orthologs were obtained from the NCBI protein database (National Center for Biotechnology Information). The accession numbers are provided in Supplementary Table 3. The *Tetrahymena* Rsp orthologs were obtained from the Tetrahymena Genome Database (https://tet.ciliate.org/). Protein amino acid sequences were aligned using ClustalX2 software [4] and edited using SeaView [5]. The identical and similar amino acid residues were shaded using GeneDoc [6] and the following color code: white letter on the black background (100% conserved residues); white letter on a grey background (80% conservation); black letter on a light grey background (60% conservation). The color lines show the position of the indicated domains.

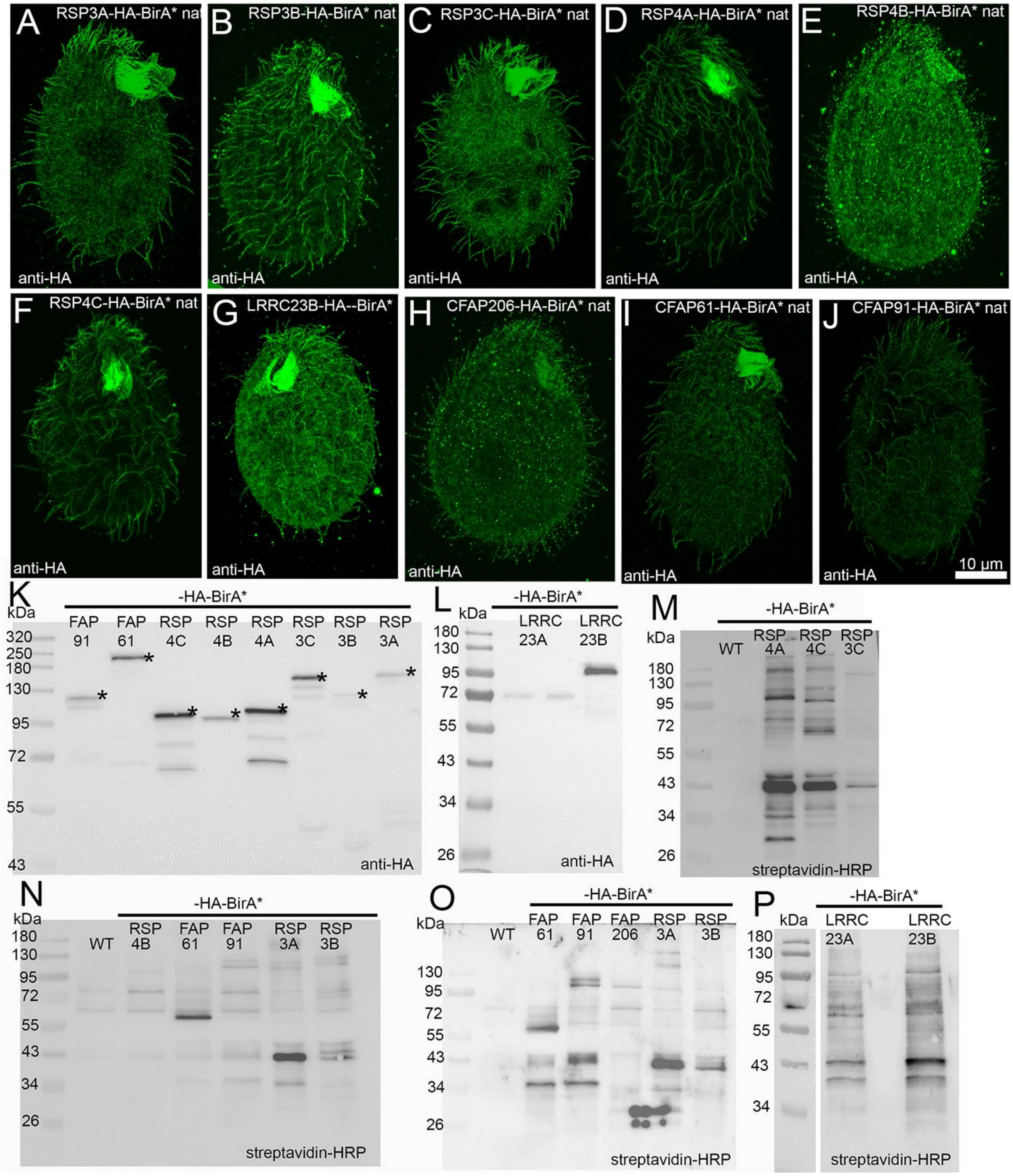

Supplementary Fig. 11. (**A-J**) Immunofluorescence confocal images of *Tetrahymena* cells expressing RS proteins as C-terminal –HA-BirA* fusions under the control of respective native promoters detected using anti-HA antibodies. (**A**) Rsp3A-HA-BirA*, (**B**) Rsp3B-HA-BirA*, (**C**) Rsp3C-HA-BirA*, (**D**) Rsp4A-HA-BirA*, (**E**) Rsp4B-HA-BirA*, (**F**) Rsp4C-HA-BirA*, (**G**) Lrrc23B-HA-BirA* (**H**) Cfap206-HA-BirA*, (**I**) Cfap61-HA-BirA*, (**J**) Cfap91-HA-BirA*. (**K-L**) Western blot analyses of the ciliary proteins isolated from *Tetrahymena* cells expressing RS proteins as –HA-BirA* fusions under the control of respective native promoters. A star indicates a band corresponding to the position of fusion protein. (**N-P**) Western blot-based analyses of the biotinylated ciliary proteins in either WT cells or cells expressing Rsp-HA-BirA* under the control of the respective native promoter, all grown for 4 hrs in a 10 mM Tris-HCl buffer (pH 7.4) supplemented with biotin, detected using HRP-conjugated streptavidin.

LRRC23

```
                     IQ
                10        20        30        40        50        60        70        80        90       100
TtLrrc23A : -----------------MSKKEKKPAPKDAKKVDEK-----PKINFEELIMKNLSEPKLSIDRTGIHETVLDLNSKSIDSIQAIFANYKALTKIDIHANN :  78
TtLrrc23B : ----MSEQEIEAEEGQVVQPEEVDEEQDDIYPPEEE-----QRRLTHEILKKGLERIGKIWTGKNYALVNMNCEKKKIKNLFNILFEYPHIRQINFSNNW :  91
MmLrrc23  : MSDEDDVDDVDAEQDEVESDKEIEEWEDYRKETEEASEEWLETPITEAMMKEGLSLICKIGSGLAHAYIKLEAKDRDITDIS-LIRSYIHLRYVDISENH :  99

               110       120       130       140       150       160       170       180       190       200
TtLrrc23A : IQDITVLGNLPNLIWLDVSQNNIKDLKSLQNEEGFRNLKYLNISKNRISELLTPKAPNLIHLNLNENLVDKMETFEG--HESLKILELRGNRIQTTQQLV : 176
TtLrrc23B : ISDISTVTSIKYLTHLNLTNNFIENLDAFRVEDTLTELEDLNLTGNKIKELVNIEAKNLQRLNLSSNEITTCENFTG--HQSINVLILKKNKLKNLKGIQ : 189
MmLrrc23  : ITDISPLNSLTHLLWLKADGNQLRSARMNELE----YLQIASFSYNQIIDTEGIFHERLGSLDLKGNRIHQVTGLDPERLSSLHTLELRGNQLESTKGIY : 195

               210       220       230       240       250       260       270       280       290       300
TtLrrc23A : NMPKLQELYLTANKIKTVVGIDSLVSLTKLHLRLNNIEQFEEN-------------------------------------------FPNLENLQYLNLRE : 233
TtLrrc23B : NMRNLEQIYAAENPITNFYDLNNLPHLKKLHLRKTEIKNLDTTKPQEVQKEEGEEGAEEENQQKEEKKRIILRPEQDQIEKELLKQMPHLPLLHYINLRE : 289
MmLrrc23  : -LPKLKNLYLAQNLLKKVEGLENLSNLTTLHLRDNQIETLNGFS-------------------------------------------QEMKSLQYLNLRS : 251
                                                                                     coiled coil (CC)
               310       320       330       340       350       360       370       380       390       400
TtLrrc23A : NKIDKFEEILKLAALPNLKTLVHSFNPLINKNPNYLYETING------LLKLQRINKVEVTRSLKLN---------AEKYAEDKWRVQEEEKKRLEEEER : 318
TtLrrc23B : TKVYDVKEVFLFQAFPNLTSINVIGTDLAESTEHSIKPELIMNNKQLKHINKEVVEEEEVTEALNKRKERDEELVRQYEEEQERLKAEEEERQRLLEEER : 389
MmLrrc23  : NMISDLAELAKLRDLEKLRALVLLDNECADETD----------------YRQEALVQMAHLERLDK------------EEYEDDDRAEAEEIRQRLKEEQ : 323

               410       420
TtLrrc23A : ---LKQEQEQQEEN------------ : 329
TtLrrc23B : ERLLEEERQRKEEEAAAAQNQQQQDE : 415
MmLrrc23  : DQDLDPDQDMEPYLPPV--------- : 340
```

STPG2

```
                10        20        30        40        50        60        70        80        90       100
TtStpg2 : MAFVFRSEK------KASHIRTTTNCLIGPGSYVQHQ-QYTIQKAYAPFN--TKEDRSGKKNKI-VELFPGPGQYQIEKSNLNEKVVVSSANDDIKIVEV :  90
Cr      : MSHAIEAATGGFVYRAGRSPQRAVAAQPGPGSYDLAR-GGDVNPAPAPFH--TSTERALASNQTTAAITPGPGAYSGPGSGIGTSGTVAG-----FVSGV :  92
MmStpg2 : MYDRAPR-------WLDCANRGSTEEHVGPGTYQVPFPKQQATGCYAPFLSLSSKTSACVVSSLAGQAVPGPAHYNVSQACYNIRG----------GRSL :  83

               110       120       130       140       150       160       170       180       190       200
TtStpg2 : PKPQANFKSQTKRFQNNEVKAKEQLPGPGQYEQEDQLKKMLEKGQSQFQNYQKQNIIDKIMKMNRYQSTPSIPSKQHTFGYTETENNDLAINKIPGHQYL : 190
Cr      : PRLPADY----------ELRARKEVPGPGAYDDGN----QWVKGTNRYKPEGGSGMARALHLLPRRPTAPSVPGRGESYGYEEAQDGSLVLQPAP-LQHT : 177
MmStpg2 : QNREKREK-----------KLISDGPGPGSYNWPY-------LGTLCITTRQKT---PRTPAVSRNIDIPSIPSSGKSHGYHLNDDDTIMRRTPE----- : 157

               210       220       230       240       250       260       270       280       290       300
TtStpg2 : GTQKDSVGPGHYIQKTFIEQQKLKGVPWHKLQAARMQNPISKSTLTVGPGSYDVQTQGLPLYKMKQSPGFASKTLRQIENRKGAVASQIVKQRMAMSQQS : 290
Cr      : GVGRDIAGPGTYELAGPGEFSRPSGTAWATSKSGRST---KFGSPAPGPGTYNLADSGP-----RRRGAGLLVAIGGVEVVFGGTTG-----TSSFVSKA : 264
MmStpg2 : -PSDNTIGPAYYNPQFDYEKASLKYKGVNFGNATGRQEFLKYSG--PGPGQYDIIQKRK-------------LHCENINIKREQEHN-----YYTYVPRL : 236

               310       320       330       340       350       360       370       380       390       400
TtStpg2 : NKNIPLNEEDSDSEEEYIEDAVPGPGHYNFE-NSSFMKANNTHSHSAGNFGSLSKRFTQSNFSNPIGPGEYN-EAVAG----IIGKNEHEVKVRNPPELS : 384
Cr      : PRPLQKSAPASPGPGEYHPPVVTAGDPAGAG-LRASAAVFGSSAARGSWEMDPTQVRSAPSYWRTPGPGSYDDPRAAGRRGSPNGASALAAAAAAAAAPF : 363
MmStpg2 : YEAIILQEEKKG---------VPGPGKYNIKSEFDMIKSMSALVNSPSEIFFSETERFEEIKSCTEAPGTYNEIRTAF-------KCEKKRFGLSLPENQ : 320

               410       420       430       440       450       460       470       480       490       500
TtStpg2 : SDTREQIKKIEEVKPGPGAYEE--RINLEDKLIQKIQKG-YRGNFGSTERRFKN---------------------ANVVDEQPGPGAYIDIATQQKFDEL : 460
Cr      : TTTALRFGSVGSAAPGPGQYHEDAVASLEYDTFKRVTGSRHAGGFGGSTGRESYNSGTSSPKRIGVPPVLGAGEGGEGLGDTPGPGAYSTDAKLGGTGRS : 463
MmStpg2 : SAARETEDSKAQKLPGPGFYDIS-TNIVKAQVKKPCLKKQPKTGFGSSVPRTLET-------------------AQKKAFRGPGPSDYQVRGTHDELPNL : 400

               510       520       530       540       550       560       570       580       590       600
TtStpg2 : EAQLPTSVFKSSSQRAIFKNQKDQKPPVGSYKLNYYDIEKKVNQ-----EPDDPDIKIKVPNLGFNTSDVRFKGPDPKKITDEDEEE---LNVIKEKR-- : 550
Cr      : VGRGGTSTFASRTGRFRPVTAPPAVPDLTDFDGAAAVKGDPSRLGPGAYSPERPTGRTRYTQVGAKSVPFGSGAKRAAMELPSKETPGPGRYGAAGDP-- : 561
MmStpg2 : N---KSAAFLSRAEKTPPVRKMRLPAPGRYDVQKSYDMSQVKHKYMPPRTSVAKKRHSSFLSAAPRCLGKIADGPGPATYSPVLMKSGAIISFVKGPKRF : 497

               610       620       630       640       650       660
TtStpg2 : ------KEETKFKKENKHFLSKADRFNYKLKNQGPSPGQYYDNAANPWNKRTFNIHEADF---- : 604
Cr      : ------HKPVSVPPPKAGFATQSDRFGPGAPKYTPGPGAYLGPGSGPVRK-SYNVTYGA----- : 613
MmStpg2 : QEFHGEFSEGPTTYELSPFLRHSLLKRTYNVTLPCSSSPNRENTGCPSQKATQKFQREKLQYFN : 561
```

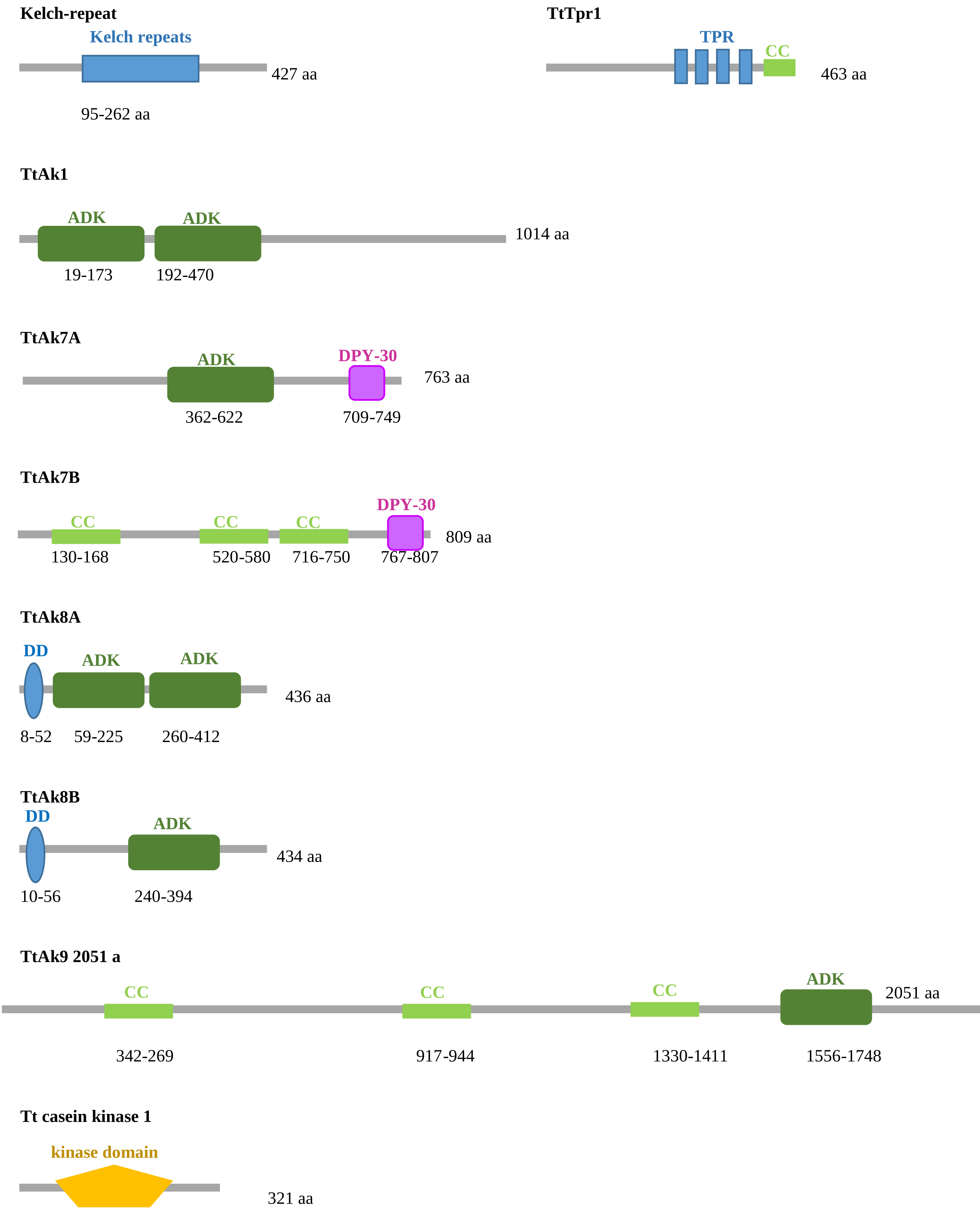
Kelch-repeat
Kelch repeats
427 aa
95-262 aa
TtTpr1
TPR
CC
463 aa
TtAk1
ADK
ADK
1014 aa
19-173
192-470
TtAk7A
ADK
DPY-30
763 aa
362-622
709-749
TtAk7B
CC
CC
CC
DPY-30
809 aa
130-168
520-580
716-750
767-807
TtAk8A
DD
ADK
ADK
436 aa
8-52
59-225
260-412
TtAk8B
DD
ADK
434 aa
10-56
240-394
TtAk9 2051 a
CC
CC
CC
ADK
2051 aa
342-269
917-944
1330-1411
1556-1748
Tt casein kinase 1
kinase domain
321 aa

Supplementary Fig. 12. Domain analyses of RS candidate proteins. Multiple alignments and domain analyses of RSP orthologs. Domain organization is predicted using SMART (http://smart.embl-heidelberg.de/) [1, 2] or UniProt (htttps://www.uniprot.org) [12]. Domains are marked according to position in *Tetrahymena* proteins. The amino acid sequences of RSP orthologs were obtained from the NCBI protein database (National Center for Biotechnology Information). The accession numbers are provided in Supplementary Table 3. The *Tetrahymena* Rsp orthologs were obtained from the Tetrahymena Genome Database (https://tet.ciliate.org/). Protein amino acid sequences were aligned using ClustalX2 software [4] and edited using SeaView [5]. The identical and similar amino acid residues were shaded using GeneDoc [6] and the following color code: white letter on the black background (100% conserved residues); white letter on a grey background (80% conservation); black letter on a light grey background (60% conservation). The color lines show the position of the indicated domains**.**

Supplementary Tables

Supplementary Table 1. Data collection and analysis parameters for cryo-ET and subtomogram averaging.

**Method**	**Subtomogram Averaging**			
**Dataset**	***WT***	***RSP3A-KO***	***RSP3B-KO***	***RSP3C-KO***
**Microscope**	Titan Krios	Titan Krios	Titan Krios	Titan Krios
**Electron Detector**	Gatan K3	Gatan K3	Gatan K3	Gatan K3
**Zero-loss filter (eV)**	20	20	20	20
**Magnification**	42,000	42,000	42,000	42,000
**Voltage (keV)**	300	300	300	300
**Exposure (e/A^2)**	160	160	80	160
**Defocus (µm)**	1.0-3.0	3.5-5.0	3-4	2.5-4.0
**Pixel size (Å)**	2.12	2.12	2.12	2.12
**Tilt range (increment)**	-60° - 60° (3°)	-60° - 60° (3°)	-60° - 60° (3°)	-60° - 60° (3°)
**Tilt scheme**	dose symmetric	dose symmetric	dose symmetric	dose symmetric
**Tilt series acquired**	58	37	50	38
**Subtomo averaged**	2608	2099	2093	2790
**Symmetry imposed**	C1	C1	C1	C1
**Map resolution (Å)**	19	20	22	17

Supplementary Table 2. Global mass spectrometry analyses (LFQ) of wild-type (WT) and RS mutant showing total number of peptides.

		Number of total and unique peptides (X/Y) in RS knock-out mutant cilia																						
Protein	TGD accession numbers	WT1	WT2	RSP 3A-1	RSP 3A-2	RSP 3A-3	RSP 3B-1	RSP 3B-2	RSP 3B-3	RSP 3C-1	RSP 3C-2	RSP 3C-3	WT1	WT2	WT3	CFAP 206	CFAP 206	CFAP 206	CFAP 91	CFAP 91	CFAP 91	CFAP 61	CFAP 61	CFAP 61	WT	RSP 2
Rsp1	TTHERM_000196029	0	20	17	24	21	13	14	15	20	20	21	19	20	21	15	14	14	12	10	13	17	17	17	15	13
Rsp2	TTHERM_00394410	45	46	29	27	31	24	30	24	27	37	36	50	46	47	36	31	32	40	48	40	46	49	53	88	9
Rsp3A	TTHERM_01044600	21	15	0	0	0	7	10	9	11	14	16	17	17	15	15	8	13	18	18	17	18	18	22	25	24
Rsp3B	TTHERM_00566810	22	24	28	25	29	0	0	0	27	26	24	40	37	36	26	20	23	19	16	15	31	30	30	52	53
Rsp3C	TTHERM_00418270	43	41	26	25	35	30	35	33	0	0	0	46	42	43	4	2	4	32	30	22	49	45	47	61	41
Rsp4A	TTHERM_00427590	36	35	25	22	23	24	26	27	23	30	30	50	42	49	32	37	35	39	38	36	44	46	47	75	53
Rsp4B	TTHERM_00502580	16	18	6	6	8	9	14	13	1	3	2	15	14	13	8	8	8	12	11	11	17	13	16	29	24
Rsp4C	TTHERM_00444180	23	25	15	10	15	5	5	5	17	23	20	29	25	27	22	19	23	25	26	25	29	27	28	34	24
Rsp7A	TTHERM_00670610	10	10	9	10	7	10	8	9	8	9	8	17	15	18	9	8	8	6	6	5	15	14	13	40	45
Rsp7B	TTHERM_00492840	15	15	8	7	10	8	12	12	9	10	11	12	11	11	9	7	9	1	1	0	13	9	12	43	30
Rsp8	TTHERM_00313520	15	14	5	3	6	1	1	1	1	2	2	8	9	10	2	1	2	2	2	1	13	11	11	35	25
Rsp9	TTHERM_00430020	15	14	18	17	19	12	11	11	20	20	15	23	25	22	17	19	18	16	17	19	21	23	19	54	41
Rsp10	TTHERM_00378600	25	23	10	10	12	7	11	11	12	15	12	23	20	21	18	18	20	21	19	19	23	24	24	33	30
Rsp11	TTHERM_00540090	5	4	3	2	3	3	4	2	2	5	4	5	5	7	2	2	1	2	4	2	6	5	6	8	4
Rsp12A	TTHERM_01018330	24	20	15	18	12	7	9	11	1	1	1	26	24	23	6	6	8	2	2	0	22	23	24	16	3
Rsp12B	TTHERM_00466090	8	2	0	1	0	0	0	0	3	5	3	7	4	3	5	2	4	3	5	5	9	8	9	2	0
Rsp14	TTHERM_000773538	17	18	8	3	6	6	11	9	10	17	14	18	15	15	12	14	14	15	12	12	13	13	15	29	29
Rsp15	TTHERM_00695730	13	14	7	5	8	0	0	0	4	9	8	13	9	10	0	0	0	1	1	0	11	12	12	14	10
Rsp16A	TTHERM_00471260	30	30	15	15	13	16	18	16	16	25	23	28	25	29	25	22	20	30	29	29	32	34	36	41	35
Rsp16B	TTHERM_00238810	21	17	18	21	22	19	16	17	8	7	5	19	22	19	8	9	9	15	16	13	19	18	17	24	18
Rsp20/CaM1	TTHERM_00630500	3	2	1	1	0	1	1	1	0	0	2	1	1	1	2	2	1	6	7	4	2	1	2	40	39
Rsp22	TTHERM_000649439	9	10	7	10	8	7	7	7	6	8	8	17	11	13	9	8	10	9	7	8	12	10	14	21	13
Rsp23/Cfap67A	TTHERM_000372529	18	16	12	13	12	12	14	12	11	15	15	18	15	14	14	18	17	13	13	13	14	13	13	39	25
Cfap61	TTHERM_00641200	61	64	45	42	51	54	59	59	49	64	51	64	53	59	56	54	51	11	9	6	0	0	0	90	76
Cfap251	TTHERM_01262850	51	47	41	41	46	37	37	44	42	45	41	47	45	51	39	40	43	0	0	0	46	49	45	54	53
Cfap91	TTHERM_00578560	33	34	28	27	32	33	26	29	31	33	23	36	27	33	25	21	26	0	0	0	34	35	40	52	42
Cfap206	TTHERM_00820660	31	35	20	21	24	7	2	4	19	21	17	28	28	27	0	0	0	1	1	1	36	34	34	53	40
Cfap253	TTHERM_00316930	26	25	12	10	12	19	22	22	11	15	16	21	18	24	19	16	19	19	19	19	23	25	23	26	24
Cfap207	TTHERM_00529880	20	18	17	17	21	8	4	7	21	21	15	22	21	21	2	0	2	1	1	2	19	21	21	19	9
Cfap198A	TTHERM_01092470	9	8	0	0	1	0	0	0	7	10	8	9	9	10	6	6	7	7	7	4	7	8	8	10	11
Cfap198B	TTHERM_00476710	3	5	4	4	4	2	3	3	2	2	3	8	7	6	3	4	2	4	4	5	7	6	6	17	11
TtTpr	TTHERM_00623040	11	12	3	6	10	8	11	9	0	0	0	10	9	10	1	1	2	5	5	5	9	10	9	19	20
Kelch-motif	TTHERM_00760390	15	13	11	9	14	12	11	13	13	16	15	19	13	17	15	19	20	1	2	1	15	17	19	25	20
Lrrc23A	TTHERM_000703669	11	11	11	12	6	9	12	10	5	9	11	11	13	13	12	7	10	0	0	1	10	10	11	23	24
Lrrc23B	TTHERM_00105260	13	11	8	6	8	7	7	8	5	10	9	15	14	13	10	7	9	0	1	0	12	11	13	31	24
Lrrc	TTHERM_01084360	14	10	5	3	6	1	0	0	6	3	4	9	8	10	2	4	4	0	0	0	2	5	6	10	10
Stpg1	TTHERM_00549640	12	8	3	7	2	1	0	1	8	7	5	12	8	11	3	0	2	0	0	0	6	3	6	4	2
Stpg2	TTHERM_00420710	19	16	15	16	10	7	9	7	22	14	19	30	26	28	18	15	19	11	9	8	25	27	25	7	16
Ak1	TTHERM_00317200	78	76	59	50	64	54	59	55	61	69	61	84	73	80	61	60	65	59	68	62	83	78	79	175	152
Ak7A	TTHERM_00558060	28	29	23	24	24	26	27	24	22	23	25	42	37	38	28	31	33	24	22	21	35	35	38	67	74
Ak7B	TTHERM_000569069	18	21	13	15	13	12	15	14	14	17	16	19	21	19	15	15	17	1	0	0	16	16	17	35	28
Ak8A	TTHERM_00227800	36	35	27	26	31	21	23	24	26	33	30	40	39	38	28	29	27	27	29	29	35	31	33	78	68
Ak8B	TTHERM_00455540	21	19	0	0	2	13	11	13	13	15	16	20	19	16	17	16	16	16	15	17	22	21	19	40	40
Ak9	TTHERM_00148750	84	89	55	53	59	65	70	67	54	68	62	94	82	88	74	61	58	1	0	0	77	81	75	137	109
Ck1	TTHERM_00938880	11	11	6	6	7	7	10	8	6	8	8	9	8	8	7	6	8	0	0	0	9	8	8	15	16
PKA catalytic sub	TTHERM_00433420	7	6	8	7	5	2	6	3	4	5	11	23	19	20	12	12	12	10	9	11	21	22	21	18	30
PKA catalytic sub	TTHERM_00658860	5	5	5	3	2	1	5	0	2	4	7	14	12	13	8	9	7	8	7	7	13	13	17	20	23
PKA regulatory sub	TTHERM_00623090	36	32	34	34	23	7	5	5	26	29	36	64	63	68	46	39	42	25	26	24	55	52	54	61	76

Supplementary Table 3. *Tetrahymena* RSP orthologs

Protein	TGD accession numbers	UniProt / NCBI accession numbers	Bait e-value	Length (residues)	Mass (kDa)	pI	Domains in Tt orthologs	C.reinchardtii ortholog	C.reinchardtii UniProt/NCBI Accession number	M.musculus Ortholog	M.musculus UniProt/NCBI Accession number
Rsp1	TTHERM_000196029 TTHERM_00196025	W7X469 / XP_012653365.1	CrRSP10 1.78444e-19	221	26	6.3	MORN	RSP1	Q27YU0 / XP_001693353.1	Rsph1	Q8VIG3 / NP_079566.1
Rsp2	TTHERM_00394410	Q233B2 / XP_001011913.1	MmRsph2 2.0127e-05	628	75	4.7	DPY-30 CC	RSP2	Q6UBQ3 / XP_001702718.1	Rsph2 Dydc2	Q9D3X8 / NP_081993.1
Rsp3A	TTHERM_01044600	Q22CF9 / XP_001030633.2	CrRSP3 2.61477e-19	764	90	4.9	radial spoke 3	RSP3	A8J2J7 / XP_001695406.1	Rsph3b	Q9DA80 / NP_001077414.1
Rsp3B	TTHERM_00566810	I7M308 / XP_001022082.2	CrRSP3 2.4593e-41	691	80	5.1	Radial spoke 3				
Rsp3C	TTHERM_00418270	Q22NW8 / XP_001007288.2	CrRSP3 8.91033e-20	950	111	5.3	ARF radial spoke 3				
Rsp4A	TTHERM_00427590	Q23A99 / XP_001013838.2	CrRSP4 1.56162e-13 CrRSP6 2.18558e-12	493	57	4.9	Radial spoke 4/6	RSP4	A8I550 / XP_001700728.1	Rsph4a	Q8BYM7 / NP_001156429.1
Rsp4B	TTHERM_00502580	I7M9L5 / XP_001022312.3	CrRSP4 5.89158e-26 CrRSP6 2.55436e-20	486	57	4.7	Radial spoke 4/6	RSP6	A8I554 / XP_001700729.1	Rsph6a	Q8CDR2/ NP_112545.2
Rsp4C	TTHERM_00444180	I7M3F3 / XP_001023280.2	CrRSP4 2.26135e-31 CrRSP6 3.55275e-33	463	54	4.9	Radial spoke 4/6				
Rsp7A	TTHERM_00670610	I7MMP3 / XP_001026382.1	CrRSP7 3.69931e-05	335	39	4.7	RIIa IQ	RSP7	PNW81221.1 (A0A2K3DL29**)**	SPA17	Q62252 / NP_035579.1
Rsp7B	TTHERM_00492840	I7M9W2 / XP_001023176.2	CrRSP7 7.40409e-05	557	66	5.2	AKAP28 Ef hands			AKAP14	Q3V0I7 NP_001028957.1
Rsp8	TTHERM_00313520	Q22KC9 / XP_001033533.2	CrRSP8 4.83879e-16	500	58	6	Armadillo	RSP8	Q27YU6 / XP_001701869.1	nd	nd
Rsp9	TTHERM_00430020	Q231F8 / XP_001011326.2	CrRSP9 7.0382e-12	296	34	5	nd	RSP9	Q27YU5 / XP_001690441.1	Rsph9	Q9D9V4 / NP_083614.1
Rsp10	TTHERM_00378600	Q23FF7 XP_001015441.2	CrRSP10 1.90468e-19	227	27	5.3	MORN	RSP10	Q27YU4 / XP_001702125.1	Rsp10b	E9PYQ0
Rsp11	TTHERM_00540090	I7MDI8 / XP_001007934.2	CrRSP11 1.85137e-11	76	9	7.9	RIIa	RSP11	Q27YU3 / XP_001698630.1	Rsph11 Ropn1l	Q9EQ00 / NP_665851.2
Rsp12A	TTHERM_01018330	Q22XP3 / XP_001010281.2	CrRSP12 3.42243e-19	1110	126	9.3	CC, PPI, FBPase	RSP12	A8I2U9 / XP_001699890.1	Rsph12 Ppil6	Q9D6D8 / NP_082706.1
Rsp12B	TTHERM_00466090	I7MIU8 / XP_001025007.2	CrRSP12 4.4317e-18	882	102	9.0	PPI				
Rsp14	TTHERM_000773538 TTHERM_00773533	W7XHA5 / XP_012655051.1	CrRSP14 0.535804	354	41	5.7	Armadillo	RSP14	A8HNV0 / XP_001690282.1	Rsph14	Q9D3W1 / NP_001157006.1
Rsp15	TTHERM_00695730	Q24C99 / XP_001025584.1	CrRSP15 5.73734e-19	409	47	5.7	LRR	RSP15	A0A2K3DEQ5 / PNW79021	Rsph15 LRRC34	Q9DAM1 / NP_082217.1
Rsp16A	TTHERM_00471260	I7MGN3 / XP_001033013.2	CrRSP16 1.38908e-43	334	38	6.3	Dnaj	RSP16	A8IKR9 / XP_001690875.1	Rsph16 Dnajb13	Q80Y75 / NP_705755.2
Rsp16B	TTHERM_00238810	I7M3Y8 / XP_001024806.2	CrRSP16 4.01687e-40	340	39	6.2	Dnaj				
Rsp20/CaM1	TTHERM_00630500	Q241P0 / XP_001022775.2	CrRSP20 7.97936e-92	149	17	4	EF hand	RSP20 calmodulin	A8IDP6 / XP_001703420.1	Rsph20 Calm1	P0DP26 / NP_033920.1

Rsp22/LC8	TTHERM_000649439	W7X4R1 / XP_012653183.1	CrRSP22 7.5e-56	91	10	6.8	LC	RSP22 LC8	A8JH45 / XP_001702907.1	Rsph22 Dynll2	Q9D0M5 / NP_080832.1
Rsp23/ Cfap67A	TTHERM_000372529 TTHERM_00372533	W7XGD1 / XP_012651451.1	CrRSP23 9.32902e-33	372	43	6.2	NDK	FAP67	Q69B19 / XP_001698136.1	Rsph23 Nme5	Q99MH5 / NP_542368.2
Cfap61	TTHERM_00641200	Q23F13 / XP_001015337.1	CrFAP61 5.54686e-36	1699	198	5.4	DUF4821, CC FAD/NAD(P) binding	FAP61	A8IF44 / XP_001703513.1	Cfap61	Q8CEL2 / XP_030108106.1
Cfap251	TTHERM_01262850	Q24DE2 / XP_001026044.2	CrFAP251 3.4439e-75	996	109	5	WD40	FAP251	A8IRK7 / XP_001691834.1	Cfap251 WDR66	E9Q743 / NP_001357769.1
Cfap91	TTHERM_00578560	I7LWP7 / XP_001022857.1	CrFAP91 1.59648e-19	644	76	7.3	PaaSYMP CC	FAP91	A8IH47 / XP_001690436.1	Cfap91 MAATS1	Q8BRC6 NM_001081025.1
Cfap206	TTHERM_00820660	Q23H79 / XP_001016174.1	MmCfap206 3.71623e-63	635	73	6	FAP206 domain	FAP206	A0A2K3DUY6 / PNW84338.1	CFap206	Q6PE87 / NP_001074494.1
Cfap253	TTHERM_00316930	I7M989 / XP_001021374.2	CrFAP253 1.1375e-102	404	49	9	IQ	FAP253	A0A2K3D359 / PNW74974.1	Iqub	Q8CDK3 / NP_766123.2
Cfap207	TTHERM_00529880	I7MCW2 / XP_001032699.2	CrFAP207 2.1404e-46	411	48	4.9	MORN	FAP207	A0A2K3DJP7 / PNW80755.1	Morn3	Q8C5T4 / NP_083388.1
Cfap198A	TTHERM_01092470	Q24BN4 / XP_001025454.2	CrFAP198 1.34852e-59	237	28	5	Cyt-b5	FAP198	A0A2K3DCN8 / PNW78294.1	Cyb5d1	Q5NCY3 / NP_001038990.1
Cfap198B	TTHERM_00476710 TTHERM_00476715	I7MEL3 / XP_001017378.2	CrFAP198 2.05639e-56	232	17	8	Cyt-b5				
TtTpr	TTHERM_00623040	Q240Y0 / XP_001022529.2	-	463	54	6	TPR	nd	nd	nd	nd
Kelch-motif	TTHERM_00760390	I7M609 / XP_001031655.2	-	427	48	5.3	Kelch repeat	nd	nd	nd	nd
Lrrc23A	TTHERM_000703669 TTHERM_00703665	W7X6G9 / XP_012655513.1	MmLrrc23 5.55138e-27	329	38	8.4	LRR	nd	nd	Lrrc23	O35125 / NP_001289484.1
Lrrc23B	TTHERM_00105260	Q234H2 / XP_001012276.1	MmLrrc23 2.11729e-11	415	49	4.98	LRR	nd	nd		
Lrrc	TTHERM_01084360	Q22BT2 / XP_001030431.1	MmLrrc74B 9.70234e-18	1014	117	6.4	LRR	nd	nd	Lrrc74B	Q14BP6 NP._001138907.1
Lrrc	TTHERM_00046820	Q23DK9 XP_001014677.2	-	401	46	5.6	LRR	nd	nd	nd	nd
RIIa domain-containing protein 1	TTHERM_00537370	I7MLY6 XP_001023547.1	Mm RIID 2.1451e-13	137	16	5.8	DD-RIIAD1	nd	nd	RIIa domain-containing protein 1	Q3KNY5.1 NP_001404721.1
MRNN04	TTHERM_00324550	Q237E4 XP_001013042.2	MmMORN5 1.01265e-39	197	23	7	MORN	nd	nd	MORN5	Q9DAI9 NP_083585.1
Stpg1	TTHERM_00549640	I7M0B4 / XP_976712.2	-	349	39	9.8	PGP motif	nd	nd	nd	nd
Stpg2	TTHERM_00420710	I7M6R0 / XP_001033326.2	MmStpg2 1.7267e-05	604	69	9.4	PGP motif	CHLRE_09g41 5650v5	A0A2K3DFZ1 XP_001696826.2	Stpg2	Q8C8J0 NP._941061.1
adenylate kinase 1, Ak1	TTHERM_00317200	I7M2R5 / XP_001021401.2	Cr 5.58533e-48	1014	119	5.4	ADK	CHLRE_01g02 9750v5	A0A2K3E6L7 XP_042928532.1	Ak1	Q9R0Y5 / NP_001185719.1
adenylate kinase 7A, Ak7A	TTHERM_00558060	I7MLH8 / XP_001022370.2	MmAk7 3.68184e-72	763	88	5	ADK DPY-30	-	-	Ak7	Q9D2H2
adenylate kinase 7B, Ak7B	TTHERM_000569069	W7WZL8 / XP_012655235.1	MmAk7 5e-22	809	95	4.6	DPY-30 CC				
adenylate kinase 8A, Ak8A	TTHERM_00227800	Q23BQ2 / XP_001014311.2	MmAk8 7.20501e-37	436	50	6.6	DD ADK	nd	nd	Ak8	Q32M07 / NP_001029046.2
adenylate kinase 8B, Ak8B	TTHERM_00455540	I7MA87 / XP_001024160.1	MmAK8 5.04591e-17	434	51	8.5	DD ADK				
adenylate kinase 9, Ak9	TTHERM_00148750	I7LWA5 / XP_001021510.2	MmAk9 8.11956e-41	2051	240	5.6	CC ADK	nd	nd	Ak9	G3UYQ4 NP._001357742.1

caseine kinase 1,<br>Ck1,	TTHERM_00938880	Q22DQ4 /<br>XP_001031063.1	MmCk1<br>1.69772e-139	321	38	6	Kinase domain	CHLRE_12g54<br>9750v5	A8iYG8<br>XP_001693999.1	Ck1	Q07954<br>NP._620690.1
Guanylate kinase,<br>Gk1	TTHERM_00781030	I7MMN2<br>XP_001026259.1	MmGuk1<br>6.7636e-40	264	31	5.9	GuKc	CHLRE_09g39<br>4102v5	A0A2K3DEF1<br>XP_042921222.1	Guk1	Q64520<br>NP_001390764.1
Phosphodiesterase<br>PDE	TTHERM_00293350	I7M0W2<br>XP_001013067.3	MmPDE<br>5.6219e-70		111	6.3		CHLRE_13g60<br>5100v5	A0A2K3D1E3<br>XP_042917831.1	PDE1	A2ASF9<br>NP_001342073.1
PKA catalytic<br>subunit-1	TTHERM_00433420	Q231B5 /<br>XP_001011369.2	Cr<br>2.23002e-94	319	36	9	Kinase domain	CHLRE_16g66<br>9800v5	XP_001699230.2	PKA catalytic<br>subunit	P05132<br>NP_032880.1
PKA catalytic<br>subunit-2	TTHERM_00658860	I7MM26 /<br>XP_001024087.2	Cr<br>2.24894e-89	321	37	9	Kinase domain			PKA catalytic<br>subunit	P22735<br>NP._058675.1
PKA regulatory<br>subunit	TTHERM_00623090	Q240X5 /<br>XP_001022534.2	Mm<br>4.05793e-63	414	48	5	cNMP	CHLRE_03g19<br>9050v5	A0A2K3DZH4<br>XP_042926603.1	PKA regulatory<br>subunit	Q9DBC7<br>NP_001300902.1

Supplementary Table 4. Summary of co-immunoprecipitation experiments showing number of total and unique peptides.

Protein	TGD accession numbers	RSP-3HA (bait)				
		WT	Rsp3A	Rsp4A	Rsp4B	Rsp4C
Rsp1	TTHERM_000196029	0	1/1	0	0	0
Rsp2	TTHERM_00394410	3/2	15/13	10/9	9/8	1/1
Rsp3A	TTHERM_01044600	0	10/9	6/6	5/5	1/1
Rsp3B	TTHERM_00566810	0	7/7	6/6	2/2	0
Rsp3C	TTHERM_00418270	0	11/8	6/5	2/2	1/1
Rsp4A	TTHERM_00427590	0	21/13	22/17	6/6	4/4
Rsp4B	TTHERM_00502580	0	4/4	5/5	2/2	2/2
Rsp4C	TTHERM_00444180	0	18/11	8/5	2/2	1/1
Rsp7A	TTHERM_00670610	0	8/5	2/2	2/2	1/1
Rsp7B	TTHERM_00492840	0	4/3	4/3	1/1	0
Rsp8	TTHERM_00313520	0	10/8	6/6	3/3	1/1
Rsp9	TTHERM_00430020	1/1	31/11	23/11	9/7	0
Rsp10	TTHERM_00378600	0	1/1	5/5	2/2	0
Rsp11	TTHERM_00540090	0	1/1	2/2	1/1	0
Rsp12A	TTHERM_01018330	0	1/1	0	0	0
Rsp12B	TTHERM_00466090	0	0	0	0	0
Rsp14	TTHERM_000773538	0	9/5	4/3	0	0
Rsp15	TTHERM_00695730	0	4/4	0	0	0
Rsp16A	TTHERM_00471260	0	4/4	4/3	2/2	3/2
Rsp16B	TTHERM_00238810	0	0	4/4	0	0
Rsp20/CaM1	TTHERM_00630500	0	0	0	0	0
Rsp22/LC8	TTHERM_000649439	0	11/5	6/4	0	0
Rsp23/Cfap67A	TTHERM_000372529	0	14/10	4/4	4/4	3/3
Cfap61	TTHERM_00641200	0	19/15	15/13	2/2	4/4
Cfap251	TTHERM_01262850	0	7/7	11/10	9/8	4/3
Cfap91	TTHERM_00578560	0	2/2	7/6	5/5	1/1
Cfap206	TTHERM_00820660	0	2/2	5/5	0	0
Cfap253	TTHERM_00316930	0	5/4	7/7	3/3	1/1
Cfap207	TTHERM_00529880	0	2/1	4/4	3/3	0
Cfap198A	TTHERM_01092470	0	1/1	0	0	0
Cfap198B	TTHERM_00476710	0	0	0	0	0
TtTpr	TTHERM_00623040	0	1/1	0	0	0
Kelch-motif protein	TTHERM_00760390	0	4/4	6/6	3/3	1/1
Lrrc23A	TTHERM_000703669	0	4/3	3/3	0	0
Lrrc23B	TTHERM_00105260	0	4/4	0	0	0
Lrr-containing	TTHERM_01084360	0	0	0	0	0
Stpg1	TTHERM_00549640	0	0	0	0	0
Stpg2	TTHERM_00420710	0	0	0	0	0
adenylate kinase 1, Ak1	TTHERM_00317200	0	76/43	35/28	18/18	17/16
adenylate kinase 7A, Ak7A	TTHERM_00558060	0	7/7	8/6	4/3	0
adenylate kinase 7B, Ak7B	TTHERM_000569069	0	7/7	9/9	5/5	2/2
adenylate kinase 8A, Ak8A	TTHERM_00227800	0	12/10	9/8	7/7	5/5
adenylate kinase 8B, Ak8B	TTHERM_00455540	0	11/8	5/5	4/4	1/1
adenylate kinase 9, Ak9	TTHERM_00148750	0	47/37	16/16	4/4	9/9
caseine kinase 1, Ck1,	TTHERM_00938880	0	6/5	2/2	3/3	1/1
PKA catalytic subunit	TTHERM_00433420	0	4/3	0	0	2/2
PKA catalytic subunit	TTHERM_00658860	0	0	0	0	0
PKA regulatory protein	TTHERM_00623090	0	1/1	3/3	2/2	0

Supplementary Table 5. Summary of BioID experiments showing number of total and unique peptides.

Protein	TGD accession numbers	Rsp 3A	Rsp 3A	Rsp 3A	Rsp 3B	Rsp 3B	Rsp 3B	Rsp 3B	Rsp 3B	Rsp 3C	Rsp 4A	Rsp 4A	Rsp 4A	Rsp 4A	Rsp 4B	Rsp 4C	Rsp 4C	Cfap 206	Cfap 206	Cfap 206	Cfap 61	Cfap 61	Cfap 61	Cfap 91	Lrrc 23A	Lrrc 23B	Lrrc 23B
Rsp1	TTHERM_000196029	2/2	2/2	1/1	1/1	5/4	0	0	5/4	0	0	16/9	2/2	16/9	2/2	0	2/2	0	0	0	0	0	0	0	7/3	0	8/7
Rsp2	TTHERM_00394410	21/17	25/15	28/15	11/9	72/32	6/5	12/6	86/33	0	0	63/38	2/2	55/32	0	10/5	2/2	8/8	0	0	0	0	2/2	1/1	38/21	0	44/20
Rsp3A	TTHERM_01044600	16/12	16/9	21/11	6/6	46/26	1/1	2/1	52/28	0	1/1	27/18	0	25/17	0	3/2	1/1	0	0	0	0	0	0	0		0	5/5
Rsp3B	TTHERM_00566810	14/11	2/2	5/4	12/9	42/20	1/1	1/1	49/23	9/7	0	32/20	1/1	25/16	1/1	6/5	1/1	6/6	7/5	7/5	4/4	0	0	1/1	32/20	0	45/18
Rsp3C	TTHERM_00418270	0	0	0	3/3	4/3	0	0	6/5	10/7	0	25/22	0	23/20	0	1/1	0	11/11	4/4	6/5	5/5	7/6	11/8	11/9	60/27	0	77/32
Rsp4A	TTHERM_00427590	3/3	14/8	12/7	0	36/18	1/1	3/2	39/19	0	5/4	76/27	15/13	67/25	9/9	13/10	20/16	1/1	0	0	0	0	0	0	15/13	0	26/14
Rsp4B	TTHERM_00502580	0	0	0	0	1/1	0	0	1/1	0	0	26/17	1/1	22/14	8/7	0	0	0	0	0	0	0	0	1/1	14/8	0	11/7
Rsp4C	TTHERM_00444180	6/6	6/5	7/5	4/4	36/18	0	0	40/20	0	2/2	51/23	3/3	43/20	0	11/9	24/16	0	0	0	0	0	0	0	0	0	2/2
Rsp7A	TTHERM_00670610	5/2	0	1/1	4/1	12/5	0	0	13/5	11/3	0	16/4	0	14/3	0	3/2	1/1	4/2	0	0	0	0	0	0	27/4	0	20/3
Rsp7B	TTHERM_00492840	0	0	0	2/2	0	0	0	0	1/1	0	1/1	0	1/1	0	0	0	2/2	1/1	1/1	5/5	0	3/2	0	82/32	0	88/29
Rsp8	TTHERM_00313520	0	0	0	3/3	1/1	0	0	1/1	5/4	2/2	18/3	0	14/11	0	0	0	9/9	4/2	6/4	1/1	4/3	5/4	5/3	37/21	0	61/24
Rsp9	TTHERM_00430020	10/9	16/6	20/8	2/2	52/16	2/2	5/4	54/18	0	2/2	79/20	11/9	74/20	10/9	16/9	22/15	6/6	1/1	2/2	0	0	3/2	1/1	15/7	2/1	19/7
Rsp10	TTHERM_00378600	3/3	2/2	2/2	0	18/10	0	0	21/11	0	1/1	22/14	0	19/11	0	2/2	5/5	0	0	0	0	0	0	0	6/5	0	18/11
Rsp11	TTHERM_00540090	1/1	0	2/1	0	7/2	0	0	8/3	0	0	3/2	0	3/2	0	0	0	2/2	0	1/1	0	0	0	2/1	8/3	0	8/3
Rsp12A	TTHERM_01018330	0	0	0	0	0	0	0	0	0	0	0	0	0	4/4	0	0	0	0	0	0	0	0	0	0	0	0
Rsp12B	TTHERM_00466090	0	0	0	0	0	0	0	0	0	1/1	0	0	0	0	0	0	0	0	0	0	0	0	0	0	0	0
Rsp14	TTHERM_000773538	13/12	4/2	5/3	3/3	40/18	1/1	1/1	42/19	0	1/1	27/20	0	23/17	0	4/3	0	0	0	0	0	0	0	0	0	0	8/4
Rsp15	TTHERM_00695730	0	0	0	2/2	0	0	0	0	1/1	1/1	10/8	0	8/8	0	0	0	3/3	3/2	3/2	0	0	0	2/2	32/11	0	31/10
Rsp16A	TTHERM_00471260	6/5	6/4	8/5	3/2	19/11	1/1	1/1	21/12	0	0	16/11	0	14/10	0	1/1	0	2/2	0	0	0	0	0	0	18/11	0	22/10
Rsp16B	TTHERM_00238810	0	0	0	0	1/1	0	1/1	2/2	1/1	0	8/6	0	8/6	0	0	0	2/2	3/2	3/2	0	0	0	4/3	26/12	0	31/13
Rsp20/CaM1	TTHERM_00630500	0	0	0	0	9/5	0	1/1	11/6	0	0	3/3	0	2/2	0	0	0	1/1	0	0	0	0	0	0	0	0	0
Rsp22	TTHERM_000649439	1/1	5/2	6/2	1/1	26/3	0	0	28/4	3/1	2/2	20/5	0	19/15	1/1	2/2	1/1	2/2	4/2	4/2	0	0	0	7/2	26/3	1/1	33/3
Rsp23/Cfap67A	TTHERM_000372529	0	0	0	0	2/2	0	0	3/3	0	0	9/9	0	8/8	0	0	0	0	0	0	0	0	0	0	0	0	0
Cfap61	TTHERM_00641200	0	0	0	6/6	2/2	0	1/1	3/3	10/9	1/1	7/7	1/1	6/6	1/1	1/1	0	12/11	6/5	6/5	13/10	16/10	21/14	18/10	146/59	0	160/60
Cfap251	TTHERM_01262850	0	0	0	2/2	1/1	0	0	1/1	4/2	0	17/15	0	16/14	0	0	0	8/8	2/2	2/2	6/6	10/6	13/9	26/14	96/42	0	123/39
Cfap91	TTHERM_00578560	0	0	0	1/1	0	1/1	1/1	0	8/4	0	21/15	0	18/12	0	1/1	0	6/6	2/2	2/2	3/3	3/1	4/2	9/6	74/29	0	84/28
Cfap206	TTHERM_00820660	0	0	0	3/3	0	0	0	1/1	7/4	1/1	15/10	0	10/7	1/1	0	0	12/12	9/6	10/6	8/8	2/1	3/2	5/2	60/29	0	68/26
Cfap253	TTHERM_00316930	5/5	0	2/2	2/2	35/17	0	0	41/20	0	1/1	36/18	0	28/15	0	0	0	0	0	0	0	0	0	0	0	0	7/6
Cfap207	TTHERM_00529880	0	0	1/1	0	0	0	0	0	0	0	9/8	0	7/7	0	0	0	0	0	0	0	0	0	0	2/2	0	3/3
Cfap198A	TTHERM_01092470	2/2	1/1	1/1	1/1	11/5	0	0	11/5	0	0	10/9	0	8/5	0	0	0	0	0	0	0	0	0	0	0	0	0
Cfap198B	TTHERM_00476710	0	0	0	1/1	0	0	0	0	0	0	6/4	0	5/4	0	0	0	2/2	0	0	0	0	0	0	17/6	0	19/6
TtTpr	TTHERM_00623040	0	0	0	2/2	0	0	0	1/1	6/3	0	6/6	0	5/5	1/1	0	0	7/6	7/5	9/6	0	1/1	2/2	6/3	19/8	0	24/12
Kelch-motif	TTHERM_00760390	0	0	0	2/2	2/2	1/1	1/1	3/3	1/1	0	7/6	0	7/6	0	1/1	0	1/1	4/3	4/3	1/1	0	0	3/3	27/12	0	20/11
Lrrc23A	TTHERM_000703669	0	0	0	0	0	0	0	1/1	0	0	1/1	0	1/1	0	0	0	0	0	0	0	0	0	0	26/12	3/1	2/2
Lrrc23B	TTHERM_00105260	0	0	0	0	1/1	0	0	1/1	0	0	1/1	0	1/1	0	0	0	0	0	0	0	0	0	0	0	0	49/13
Lrrc	TTHERM_01084360	0	0	0	0	0	0	0	0	0	0	0	0	0	0	0	0	0	0	0	0	0	0	0	0	0	0
Stpg1	TTHERM_00549640	0	0	0	0	0	0	0	0	0	0	0	0	0	0	0	0	0	0	0	0	0	0	0	0	0	0
Stpg2	TTHERM_00420710	0	0	0	0	0	0	0	0	0	0	0	0	0	0	0	0	0	0	0	0	0	0	0	0	0	0
Ak1	TTHERM_00317200	17/15	21/9	25/11	7/7	12/12	2/2	2/2	16/15	2/2	1/1	37/29	1/1	29/23	1/1	17/12	3/3	8/8	1/1	1/1	0	0	0	0	47/27	1/1	54/31
Ak7A	TTHERM_00558060	0	0	0	0	0	0	0	0	0	0	2/2	0	2/2	0	0	0	0	0	0	0	0	0	0	11/9	0	13/8
Ak7B	TTHERM_000569069	0	0	0	0	1/1	0	0	1/1	0	0	3/3	0	3/3	0	0	0	0	0	0	0	0	0	0	28/17	25/13	9/7
Ak8A	TTHERM_00227800	5/5	16/10	16/10	7/7	36/19	7/4	8/4	41/21	7/4	3/3	37/20	2/2	30/18	3/3	7/5	7/7	16/11	13/8	14/9	1/1	6/4	10/6	23/14	67/25	0	69/23
Ak8B	TTHERM_00455540	8/7	11/7	13/8	4/4	31/19	1/1	1/1	36/20	0	1/1	24/18	1/1	22/17	0	0	1/1	0	0	0	0	0	0	0	1/1	0	16/9
Ak9	TTHERM_00148750	0	0	0	0	5/5	0	0	7/7	1/1	0	18/18	0	16/16	0	1/1	0	1/1	0	0	0	0	0	0	156/63	0	13/10
Ck1	TTHERM_00938880	0	0	0	0	0	0	0	0	0	0	3/3	0	2/2	0	0	0	0	0	0	0	0	0	0	0	0	5/4
PKA catalytic sub	TTHERM_00433420	0	0	0	0	0	0	0	0	0	0	0	0	0	0	0	0	0	0	0	0	0	0	0	0	0	0
PKA catalytic sub	TTHERM_00658860	0	0	0	0	0	0	0	0	0	0	0	0	0	0	0	0	0	0	0	0	0	0	0	0	0	0
PKA regulatory sub	TTHERM_00623090	0	0	0	0	0	0	0	0	0	0	0	0	0	0	0	0	0	0	0	0	0	0	0	0	0	0

Supplementary Table 6. *Tetrahymena* RSP orthologs identified with multiple hits in TGD. When numerous proteins were identified with similar scores in a blastp search of the *Tetrahymena* total proteome (TGD) using *Chlamydomonas* RSP as a bait, we analyzed which of those proteins are present in the *Tetrahymena* ciliome (LFQ and TMT data) and whether they co-immumoprecipitated (co-IP, Table S5) with known RSPs or are biotinylated (BioID, Table S6) in cells expressing known RS proteins as fusions with mutated BirA* ligase. Arrows indicate a significant change (↑ increase or ↓ decrease) in the protein level in studied knockout mutants (based on TMT analyses). ns – change not significant, nd – protein not detected in the *Tetrahymena* ciliome.

Bases of the identification of less conserved RSPs.

Rsp1 and Rsp10 are MORN-domain-containing RS head proteins. The *Tetrahymena* genome encodes 129 proteins having a MORN domain [13], numerous with similarity to *Chlamydomonas* RSP1 and RSP10. Only two *Tetrahymena* MORN proteins, both with higher similarity to *Chlamydomonas* RSP10, co-precipitated with Rsp4-3HA and were identified in BioID assays, clearly indicating that these proteins are RSP1 and RSP10 orthologs. Of note, using *Chlamydomonas* RSP1 as a bait, *Tetrahymena* Rsp1 and Rsp10 were identified as the 9th and 36th hits; using *Chlamydomonas* RSP10 as a bait, *Tetrahymena* Rsp1 and Rsp10 were identified as the 1st and 2nd hits (see a Supplementary Table below).

A search of the *Tetrahymena* proteome with *Chlamydomonas* RSP5 aldo-keto reductase as a query failed to identify an obvious ortholog. Thirteen proteins annotated in the Tetrahymena Genome Database (TGD) as aldo-keto reductases were present in our ciliomes, of which four were significantly reduced in the *RSP3* knockouts. However, neither of these proteins was detected in co-IP or BioID assays (Supplementary Tables 4 and 5). Thus, similar to mammals [14], *Tetrahymena* seems to lack an RSP5 ortholog. Interestingly, a search of the NCBI protein database using *Chlamydomonas* RSP5 as a query identified RSP5 orthologs only in *Chlamydomonas*-related algae. *Chlamydomonas* RSP7 has an RIIa domain (dimerization-anchoring domain of PKA regulatory subunit) at its N-terminus and several EF-hand motifs in the middle and C-terminal regions. A search of the *Tetrahymena* proteome led to the identification of two proteins, Rsp7A and Rsp7B, both with limited similarity to *Chlamydomonas* RSP7 (Supplementary Fig. 10). The Rsp7A is a 39 kDa protein containing an N-terminal RIIa and a C-terminal IQ calmodulin-binding motif, while the 66 kDa Rsp7B has a predicted AKAP (A-kinase anchoring protein) domain and two EF-hand motifs. RSP12 is a peptidyl-prolyl cis-trans isomerase (PPIase) [14]. Out of 15 *Tetrahymena* genome-encoded PPIases, seven were present in cilia, but only two, named here Rsp12A and Rsp12B, were significantly reduced in RS mutants.

		E-value (TGD blastp)	In TMT ciliome	In co-IP or BioID	RSP3A-KO	RSP3B-KO	RSP3C-KO	FAP91-KO
**Rsp1**	**TTHERM_000196029**<br>new number<br>**TTHERM_00196025**	1.78444e-19	yes	coIP 3A<br>Bir 3A, 3B, 4A, 4C	ns	↓	ns	ns
**Rsp10**	**TTHERM_00378600**	1.90468e-19	yes	coIP 4A<br>Bir 3A, 3B, 4A, 4C	ns	↓	ns	ns
	TTHERM_00338260	7.69708e-19	no	-	-	-	-	-
	TTHERM_00561720	4.08366e-17	yes	nd	ns	ns	ns	ns
	TTHERM_00086700	3.34742e-16	yes	Bir 3B (1), 4A (1)	ns	↓	↓	↓

	TTHERM_00940320	4.65599e-16	no	-	-	-	-	-
	TTHERM_00471440	3.45586e-15	no	-	-	-	-	-
	TTHERM_00522150	6.02902e-15	no	-	-	-	-	-
	TTHERM_00013150	6.59314e-15	no	-	-	-	-	-
	TTHERM_00122390	1.03558e-14	no	-	-	-	-	-
	TTHERM_01044720	4.35786e-14	no	-	-	-	-	-
	TTHERM_00058629	8.15103e-14	yes	nd	ns	↓	ns	ns
	TTHERM_00047490	8.87354e-14	yes	nd	↑	ns	ns	↓
	TTHERM_00080020	1.83831e-13	yes	nd	ns	ns	ns	ns
	TTHERM_00138540	1.85468e-13	no	-	-	-	-	-
	TTHERM_00133460	4.62151e-13	no	-	-	-	-	-
	TTHERM_00149910	5.33197e-13	no	-	-	-	-	-
	TTHERM_00691730	6.19233e-13	no	-	-	-	-	-
	TTHERM_00142460	6.24492e-13	no	-	-	-	-	-
	TTHERM_01248870	6.40885e-13	no	-	-	-	-	-
	TTHERM_00724730	1.06173e-12	no	-	-	-	-	-
	TTHERM_00078940	1.13884e-12	yes	nd	ns	↓	ns	ns
	TTHERM_00685930	1.18943e-12	yes	nd	ns	↓	↓	↓
	TTHERM_00509110	2.45575e-12	yes	nd	ns	ns	↑	ns
	TTHERM_00486410	4.33653e-12	yes	nd	ns	ns	ns	ns
	TTHERM_00590130	5.60043e-12	yes	nd	ns	↓	↓	↓
	TTHERM_00077350	6.44267e-12	yes	nd	ns	ns	ns	ns
**Rsp5**	TTHERM_00962090	none	yes	nd	↑	ns	↑	↑
	TTHERM_00338200	none	yes	nd	↑	ns	ns	↓
	TTHERM_01080330	none	yes	nd	↑	↑	ns	ns
	TTHERM_00058260	none	yes	nd	↑	↑	ns	ns
	TTHERM_00526310	none	yes	nd	↑	↑	↑	ns
	TTHERM_00300640	none	yes	nd	↑	ns	ns	ns
	TTHERM_00300630	none	yes	nd	ns	↓	↓	ns
	TTHERM_00526449	none	yes	nd	ns	ns	↑	↓
	TTHERM_00274520	none	yes	nd	ns	↓	↓	↓
	TTHERM_00266330	none	yes	nd	ns	↓	ns	ns
	TTHERM_00300620	none	yes	nd	ns	ns	ns	↑
	TTHERM_00703880	none	yes	nd	ns	↑	↑	ns
	TTHERM_01002600	none	yes	nd	ns	ns	↓	↓
**Rsp7**	TTHERM_00353490	7.66049e-13	yes	nd	ns	↑	ns	↑
	TTHERM_00686010	2.22411e-09	no	-	-	-	-	-
	TTHERM_00384910	5.51363e-09	yes	nd	↓	ns	ns	↑
	TTHERM_00632950	1.57971e-08	no	-	-	-	-	-
	TTHERM_00486220	8.14257e-08	no	-	-	-	-	↑
	TTHERM_00523059	1.054e-07	yes	nd	↓	ns	↓	-
	TTHERM_00059190	1.12651e-07	yes	nd	↓	↓	↓	↓
	TTHERM_00668030	2.30983e-07	yes	nd	ns	ns	ns	ns
	TTHERM_000442899 (Stk36-like)	4.65608e-07	yes	co-IP RSP4C (1) Bir RSP4A (1-2), 3B (1) 4B (3/3)	ns	↑	ns	↑
	TTHERM_00300120	6.8876e-07	yes	nd	ns	↑	ns	ns

	TTHERM_00630500 (CaM1)	9.98653e-07	yes	Bir 4A (2-3), 3B (1-11) F206 (1)	ns	↓	↓	ns
	TTHERM_00622900	2.83945e-06	no	-	-	-	-	-
	TTHERM_00267880 (Bbc37)	4.07889e-06	yes	coIP 3A(1), 4A(4)	ns	↓	↓	↑
	TTHERM_00194410	5.09536e-06	no	-	-	-	-	-
	TTHERM_00616570	5.2479e-06	yes	nd	↓	ns	ns	ns
	TTHERM_00218310	9.61136e-06	yes	nd	ns	↓	↓	↓
	TTHERM_00589960	1.36054e-05	yes	nd	ns	ns	ns	ns
	TTHERM_00899430	1.57e-05	no	-	-	-	-	-
	TTHERM_00495950	1.90483e-05	yes	nd	↑	ns	ns	↑
	TTHERM_01052880	2.2429e-05	yes	nd	ns	↓	ns	↑
**Rsp7A**	**TTHERM_00670610**	3.31578e-05	yes	co-IP 4A(2), 4B(2), 4C(1), 3A(8) Bir 3A(1-5), 3B(4-13), 3C(11), 4A(14-16), 4C(1-3), 206 (4)	ns	↓	ns	↓
	TTHERM_00637390	4.33175e-05	yes	nd	↑	↓	ns	↓
	TTHERM_000841299	5.08855e-05	no	-	-	-	-	-
	TTHERM_00147620	6.24849e-05	yes	nd	ns	ns	ns	ns
**Rsp7B**	**TTHERM_00492840**	6.65092e-05	yes	coIP 3A (4), 4A(4), 4B (1)	↑	↑	↑	↓
**Rsp12**	TTHERM_00051740	1.0883e-25	yes	Bir 3 A(1 pep)	ns	↑	ns	ns
	TTHERM_00997720	1.64934e-25	no	-	-	-	-	-
	TTHERM_00890120	2.17043e-25	yes	nd	↑	ns	↑	↑
	TTHERM_00243800	1.07131e-22	no	-	-	-	-	-
	TTHERM_00118710	5.99925e-22	yes	nd	ns	↓	↓	ns
	TTHERM_00492720	6.01654e-22	no	-	-	-	-	-
	TTHERM_00548130	4.03513e-21	yes	nd	ns	↓	↓	↑
	**TTHERM_01018330**	2.06701e-19	yes	coIP 3a (1) BioID4B (4)	ns	↓	↓	↓
	TTHERM_00865350	5.9945e-19	yes	nd	↑	↓	ns	ns
	TTHERM_00877050	1.9254e-18	no	-	-	-	-	-
	**TTHERM_00466090**	3.9832e-18	yes	Bir 4a (1)	↓	↓	ns	ns
	TTHERM_00075590	7.37871e-18	no	-	-	-	-	-
	TTHERM_00772040	1.51296e-17	no	-	-	-	-	-
	TTHERM_00538480	7.1862e-15	no	-	-	-	-	-

Supplementary Table 7. Comparative analyses of the levels of the RS proteins in wild-type (WT) cells and RS mutants

Radial spoke proteins	Acc numer in TGD	RSP3A-KO/WT				RSP3B-KO/WT				RSP3C-KO/WT				CFAP206-KO/WT		CFAP61-KO/WT		CFAP91-KO/WT			
		LFQ		TMT		LFQ		TMT		LFQ		TMT		LFQ		LFQ		LFQ		TMT	
		q-value	diffe-rence	q-value	diffe-rence	q-value	diffe-rence	q-value	diffe-rence	q-value	diffe-rence	q-value	diffe-rence	q-value	diffe-rence	q-value	diffe-rence	q-value	diffe-rence	q-value	diffe-rence
Rsp1	TTHERM_000196029	0.7795	-0.15	0.2168	-0.13	0.0171	-1.54	0.0016	-0.64	0.8424	-0.12	0.5512	-0.07	0.3602	-0.37	0.9789	-0.01	0.6253	0.21	0.1372	-0.13
Rsp2	TTHERM_00394410	0.4621	-0.50	0.0414	-0.29	0.0196	-1.54	0.0012	-0.59	0.3111	-0.74	0.3073	-0.13	0.3215	-0.42	0.4980	0.24	0.6309	-0.21	0.5120	-0.05
Rsp3A	TTHERM_01044600	0.0025	-5.23	0.0124	-0.99	0.0748	-1.12	0.0029	-0.40	0.7104	-0.22	0.6405	0.05	0.3774	-0.41	0.9426	0.02	0.8836	-0.08	0.0548	0.18
Rsp3B	TTHERM_00566810	0.4992	0.41	0.3772	-0.09	0.0010	-5.10	0.0019	-1.15	0.6048	0.30	0.8883	0.02	0.0768	-1.05	0.5058	-0.24	0.0040	-2.61	0.0041	-0.43
Rsp3C	TTHERM_00418270	0.9072	-0.06	0.8769	0.02	0.2197	-0.53	0.1760	-0.10	0.0009	-5.47	0.0044	-0.59	0.0085	-3.39	0.3490	0.43	0.0554	-1.69	0.0399	-0.21
Rsp4A	TTHERM_00427590	0.7013	-0.20	0.3759	-0.08	0.0320	-1.32	0.0011	-0.49	0.9359	-0.05	0.4944	0.07	0.1033	-0.83	0.7104	0.12	0.2889	-0.40	0.2864	-0.08
Rsp4B	TTHERM_00502580	0.3863	-0.80	0.9895	0.002	0.2212	-0.75	0.9626	0.00	0.0082	-4.58	0.0125	-0.79	0.0954	-0.94	0.9786	0.01	0.0484	-2.05	0.0219	-0.26
Rsp4C	TTHERM_00444180	0.3744	-0.74	0.1361	-0.18	0.0003	-3.62	0.0017	-0.68	0.5952	-0.27	0.5776	0.07	0.9839	-0.01	0.9724	0.01	0.8082	-0.11	0.6438	-0.03
Rsp7A	TTHERM_00670610	0.4480	0.61	0.0700	-0.29	0.0441	-2.04	0.0019	-0.77	0.4556	0.57	0.8805	0.02	0.0963	-1.34	0.8033	-0.08	0.0248	-1.16	0.0044	-0.48
Rsp7B	TTHERM_00492840	0.5596	-0.30	0.0471	0.25	0.7444	-0.16	0.0031	0.35	0.6300	-0.27	0.0271	0.36	0.0815	-3.00	0.3705	-0.42	0.0043	-5.51	0.0020	-0.51
Rsp8	TTHERM_00313520	0.0444	-2.31	0.2889	-0.11	0.0180	-4.38	0.0006	-1.34	0.0040	-3.86	0.0017	-0.80	0.0247	-2.51	0.0381	1.12	0.0056	-3.19	0.0006	-1.21
Rsp9	TTHERM_00430020	0.7701	-0.15	0.4773	-0.08	0.0190	-1.59	0.0024	-0.40	0.6139	-0.27	0.1883	0.16	0.4841	-0.30	0.3612	-0.37	0.1901	-0.57	0.5680	0.04
Rsp10	TTHERM_00378600	0.3694	-0.78	0.4403	-0.08	0.0148	-2.16	0.0012	-0.58	0.3030	-0.79	0.7289	-0.04	0.5293	-0.23	0.7611	0.10	0.6808	-0.18	0.2148	-0.11
Rsp11	TTHERM_00540090	0.2115	-1.53	0.0307	-0.31	0.0111	-4.64	0.0011	-0.49	0.0869	-2.04	0.0367	-0.35	0.5919	1.14	0.1085	2.76	0.6309	1.01	0.0548	-0.20
Rsp12A	TTHERM_01018330	0.9256	0.05	0.2392	0.11	0.0161	-2.27	0.0079	-0.29	0.0010	-4.46	0.0118	-0.45	0.0096	-2.20	0.5560	-0.26	0.0036	-3.28	0.0019	-0.53
Rsp12B	TTHERM_00466090	nd		0.0102	-0.48	nd		0.0000	-0.68	nd		0.0807	0.24	nd		nd		nd		0.5789	0.04
Rsp14	TTHERM_000773538	0.0377	-2.51	0.0045	-0.74	0.0146	-2.40	0.0010	-0.52	0.3733	-0.65	0.8355	0.02	0.6305	-0.24	0.1018	0.72	0.0130	1.08	0.0172	0.31
Rsp15	TTHERM_00695730	0.2194	-1.21	0.5601	-0.06	0.0120	-3.67	0.0019	-0.86	0.1759	-1.49	0.2321	-0.17	0.0080	-2.91	0.3356	-0.88	0.0042	-5.13	0.0038	-0.97
Rsp16A	TTHERM_00471260	0.0406	-2.62	0.0253	-0.33	0.0216	-2.25	0.0013	-0.56	0.0968	-1.59	0.5844	-0.07	0.3365	-0.51	0.2292	0.54	0.4597	0.35	0.4636	0.05
Rsp16B	TTHERM_00238810	0.8796	-0.08	0.3494	0.10	0.3395	-0.40	0.6381	0.03	0.0121	-5.13	0.0064	-0.67	0.0092	-1.92	0.8685	-0.05	0.0078	-1.24	0.0354	-0.22
Rsp20/CaM1	TTHERM_00630500	nd		0.1719	-0.20	nd		0.0240	-0.32	nd		0.0117	-0.67	0.1659	-2.02	0.0406	1.00	0.0049	-3.10	0.3203	0.10
Rsp22/Lc8	TTHERM_000649439	0.0666	-2.53	0.4513	-0.08	0.0439	-1.62	0.0027	-0.42	0.0461	-3.36	0.2872	0.11	0.0952	-1.00	0.2773	0.5	0.4440	-0.38	0.2457	0.08
Rsp23/Cfap67A	TTHERM_000372529	0.6087	-0.29	0.9553	-0.01	0.7482	-0.16	0.6381	0.03	0.6874	-0.26	0.6799	0.04	0.5172	-0.25	0.5812	0.19	0.9600	0.03	0.0188	0.27
Cfap61	TTHERM_00641200	0.5334	-0.39	0.0010	1.32	0.5872	-0.23	0.0002	1.41	0.5468	-0.36	0.0044	1.46	0.6809	-0.16	0.0128	-3.60	0.0044	-3.56	0.1182	0.13
Cfap251	TTHERM_01262850	0.7834	-0.15	0.3688	0.09	0.6187	-0.23	0.4797	0.04	0.9708	-0.02	0.0437	0.28	0.3754	-0.34	0.9176	-0.03	0.0049	-5.61	0.0095	-0.88
Cfap91	TTHERM_00578560	0.6777	-0.21	0.3425	0.10	0.2540	-0.54	0.1045	-0.13	0.5640	-0.34	0.2188	0.14	0.3221	-0.40	0.7473	-0.10	0.0035	-5.85	0.0089	-0.97
Cfap206	TTHERM_00820660	0.8793	-0.08	0.4950	0.07	0.0008	-4.04	0.0074	-1.04	0.6660	-0.22	0.3175	-0.16	0.0139	-4.67	0.2723	-0.46	0.0041	-4.55	0.0083	-1.37
Cfap253	TTHERM_00316930	0.6415	-0.24	0.3314	-0.09	0.8457	-0.09	0.1084	0.12	0.5416	-0.36	0.4070	0.09	0.5152	-0.45	0.3267	0.55	0.6564	0.27	0.0557	0.18
Cfap207	TTHERM_00529880	0.8223	-0.12	0.5520	0.06	0.0137	-3.31	0.0040	-0.90	0.5665	-0.32	0.9967	0.00	0.0157	-3.76	0.4082	-0.29	0.0177	-3.93	0.0024	-1.08
Cfap198A	TTHERM_01092470	0.8513	-0.09	0.0085	-0.55	0.4064	-0.34	0.0004	-0.98	0.0889	-2.42	0.0710	0.25	0.3138	-0.76	0.2017	-1.37	0.5137	0.84	0.0423	0.20
Cfap198B	TTHERM_00476710	0.8513	-0.09	0.0927	0.23	0.4064	-0.34	0.4849	0.05	0.0889	-2.42	0.0177	-0.45	0.1449	-1.85	0.2997	-1.01	0.0467	-1.51	0.1430	-0.14

Significant reduction or increase of the protein level are marked in red or blue, respectively; TGD – Tetrahymena Genome Database; LFQ – label-free quantification, TMT- tandem mass tag 10-plex isobaric mass tagging approaches.

Supplementary Table 8. Comparative analyses of the levels of potential RS-associated proteins in wild-type (WT) cells and RS mutants

Protein name	Acc number in TGD	RSP3A-KO/WT				RSP3B-KO/WT				RSP3C-KO/WT				CFAP206-KO/WT		CFAP61-KO/WT		CFAP91-KO/WT			
		LFQ		TMT		LFQ		TMT		LFQ		TMT		LFQ		LFQ		LFQ		TMT	
		q-value	diffe-rence	q-value	diffe-rence	q-value	diffe-rence	q-value	diffe-rence	q-value	diffe-rence	q-value	diffe-rence	q-value	diffe-rence	q-value	diffe-rence	q-value	diffe-rence	q-value	diffe-rence
TtTpr	TTHERM_00623040	0.4441	-0.77	0.6006	-0.05	0.1834	-0.66	0.9062	-0.01	0.0091	-3.42	0.0116	-0.56	0.0256	-2.01	0.7405	0.14	0.0683	-1.95	0.1189	-0.14
kelch-repeat	TTHERM_00760390	0.9112	0.06	0.8595	0.02	0.8452	-0.09	0.1661	0.11	0.9543	-0.04	0.2436	0.14	0.6226	-0.23	0.5444	0.23	0.0053	-3.87	0.0017	-0.52
Lrrc23A	TTHERM_000703669	0.2089	-1.02	0.0663	0.23	0.3478	-0.45	0.0339	0.21	0.2615	-1.73	0.0178	0.42	0.2243	-1.41	0.7830	-0.10	0.0035	-4.12	0.0014	-1.16
Lrrc23B	TTHERM_00105260	0.7001	-0.22	0.6874	0.04	0.3550	-0.40	0.1415	0.12	0.4826	-0.45	0.0131	0.46	0.1661	-0.60	0.0477	-0.94	0.0034	-6.02	0.0003	-1.12
Lrrc	TTHERM_01084360	0.5992	0.27	0.0605	0.26	0.1355	-0.95	0.0019	-0.40	0.4602	-0.45	0.0285	0.34	0.0682	-2.21	0.0770	-1.45	0.0085	-1.27	0.0132	-0.32
Lrrc	TTHERM_00046820	0.5539	-0.37	0.0696	0.25	0.2356	-0.59	0.0646	-0.16	0.4420	-0.64	0.3744	0.11	0.077	-1.64	**0.0328**	**-2.53**	**0.0072**	**-2.11**	**0**	**-0.73**
RIIa domain	TTHERM_00537370	0.7366	0.19	0.3208	-0.09	0.8479	0.12	0.1545	-0.10	0.8104	-0.16	0.1419	0.25	0.6208	0.55	0.0605	3.1	0.0838	1.24	0	-0.8
MRNN04	TTHERM_00324550	0.5	2.84	0.0065	0.55	0.5868	-0.27	0.0009	0.47	0.5294	0.44	0	0.71	0.3124	-0.47	0.1816	-0.61	0.003	-4.92	0	-0.65
Stpg1	TTHERM_00549640	0.8430	0.16	0.0267	-0.36	0.0337	-1.43	0.0170	-0.29	0.9056	0.08	0.0044	0.81	0.0185	-3.19	0.0546	-1.59	0.0147	-3.47	0.0354	-0.90
Stpg2	TTHERM_00420710	0.5100	0.64	0.1000	-0.20	0.1549	-0.77	0.0028	-0.38	0.3054	1.02	0.1326	-0.18	0.1105	-0.94	0.1492	-0.64	0.0089	-3.72	0.1069	-0.14

Significant reduction or increase of the protein level are marked in red or blue, respectively; TGD – Tetrahymena Genome Database; LFQ – label-free quantification, TMT- tandem mass tag 10-plex isobaric mass tagging approaches.

Supplementary Table 9. Comparative analyses of the levels of proteins with a putative enzymatic activities in RS mutants

**Enzyme name**	Acc numer in TGD	RSP3A-KO/WT				RSP3B-KO/WT				RSP3C-KO/WT				CFAP206-KO/WT		CFAP61-KO/WT		CFAP91-KO/WT			
		LFQ		TMT		LFQ		TMT		LFQ		TMT		LFQ		LFQ		LFQ		TMT	
		q-value	diffe-rence	q-value	diffe-rence	q-value	diffe-rence	q-value	diffe-rence	q-value	diffe-rence	q-value	diffe-rence	q-value	diffe-rence	q-value	diffe-rence	q-value	diffe-rence	q-value	diffe-rence
adenylate kinase 1, Ak1	TTHERM_00317200	0.2096	-1.03	0.2215	-0.12	**0.0186**	**-1.50**	**0.0010**	**-0.49**	0.2443	-0.93	0.4282	-0.09	0.3223	-0.43	0.4288	0.29	0.2574	-0.43	**0.0346**	**-0.22**
adenylate kinase 7A, Ak7A	TTHERM_00558060	0.9677	-0.02	0.0706	0.23	0.2455	-0.51	0.6449	0.03	0.9148	-0.06	0.0778	0.24	0.3148	-0.43	0.2183	-0.45	**0.0095**	**-3.57**	**0.0089**	**-0.37**
adenylate kinase 7B, Ak7B	TTHERM_000569069	0.6181	-0.27	0.0196	0.33	0.3412	-0.42	0.0116	0.28	0.5422	-0.36	0.0306	0.35	0.3047	-0.42	0.1970	-0.53	**0.0048**	**-6.74**	**0.0000**	**-0.99**
adenylate kinase 8A, Ak8A	TTHERM_00227800	0.5095	-0.41	0.4586	0.07	**0.0181**	**-1.80**	**0.0111**	**-0.28**	0.2103	-1.10	0.6136	-0.05	0.1451	-0.69	0.1838	0.54	0.6095	0.22	0.1733	-0.11
adenylate kinase 8B, Ak8B	TTHERM_00455540	**0.0003**	**-4.12**	**0.0065**	**-0.60**	0.1562	-0.73	0.0723	-0.16	0.8016	0.14	0.2832	0.12	0.5638	-0.25	0.5959	0.18	0.2885	0.41	**0.0134**	**0.31**
adenylate kinase 9, Ak9	TTHERM_00148750	0.4987	-0.40	0.0318	0.30	0.2744	-0.44	0.0031	0.35	0.4566	-0.48	0.0129	0.45	0.4154	-0.30	0.1851	-0.49	**0.0034**	**-5.47**	**0.0047**	**-0.76**
caseine kinase 1, Ck1,	TTHERM_00938880	0.3214	-1.20	0.0153	0.43	0.1869	-0.79	0.0009	0.48	0.3032	-0.90	0.0172	0.47	0.3752	-0.42	**0.0398**	**-1.77**	**0.0040**	**-3.45**	**0.0215**	**-0.32**
guanylate kinase, Gk1	TTHERM_00781030	0.5793	-0.30	0.8151	0.02	0.3717	-0.41	0.024	**0.23**	0.5442	-0.33	0.0658	0.24	0.4619	-0.33	0.4631	-0.31	**0.0047**	**-4.83**	**0**	**-1.36**
phosphodiesterase, Pde	TTHERM_00293350	0.4821	-0.49	0.0100	0.53	0.3325	-0.43	0.001	0.55	0.5061	-0.48	**0**	**0.75**	0.7072	-0.18	**0.0106**	**-4.61**	**0.005**	**-5.49**	**0**	**-0.65**
PKA catalytic subunit	TTHERM_00433420	0.7840	0.21	0.6428	-0.04	0.0831	-1.36	**0.0000**	**-0.98**	0.5403	-0.59	**0.0000**	**-0.61**	0.9273	-0.05	0.2436	0.49	0.0206	-1.50	**0.0037**	**-0.38**
PKA catalytic subunit	TTHERM_00658860	0.5455	-0.68	0.1637	-0.16	0.1845	-1.16	**0.0019**	**-0.63**	0.3813	-1.09	0.1141	-0.23	0.2830	0.55	0.3487	0.43	0.0075	-3.58	0.7250	0.03
PKA regulatory subunit	TTHERM_00623090	0.8422	0.18	0.3634	-0.09	**0.0103**	**-3.57**	**0.0035**	**-1.04**	0.8085	0.19	**0.0120**	**-0.47**	0.0945	-0.96	0.6476	-0.16	0.0073	-3.93	**0.0186**	**-0.27**

Significant reduction or increase of the protein level are marked in red or blue, respectively; TGD – Tetrahymena Genome Database; LFQ – label-free quantification, TMT- tandem mass tag 10-plex

Supplementary Table 10. Bases of the identification of RS subunits. *Tetrahymena* cross-linking data [15]. *Chlamydomonas* RS model (Rsp1-Rsp23) [16].

protein	TGD accession numbers	radial spoke	Mutant ciliomes
Rsp1	TTHERM_000196029	RS1<br>RS2	Comparative global ciliome analyses:<br>• The level of Rsp1 is diminished in *RSP3B* knockout<br>Co-IP and BioID data:<br>• Rsp1 is effectively biotinylated in cells expressing BirA*-tagged protein: Rsp3A, Rsp3B, and Rsp4A<br>Cross-link data:<br>• Rsp1 cross-links with Rsp4A and Rsp2
Rsp2	TTHERM_00394410	RS1<br>RS2	Comparative global ciliome analyses:<br>• The level of Rsp2 is diminished in *RSP3B* knockouts<br>• The level of Rsp2 is unchanged in CFAP61-KO and CFAP91-KO<br>Co-IP and BioID data:<br>• Rsp2 co-precipitates with Rsp3A, Rsp4A, and Rsp4B<br>• Rsp2 is identified among proteins biotinylated in cells expressing BirA*-tagged protein: Rsp3A, Rsp3B, Rsp4A, Rsp4C<br>• Rsp2 is poorly or not biotinylated in cells expressing RS3 protein, Cfap61-HA-BirA*<br>Cross-link data:<br>• Rsp2 cross-links with Rsp1, Rsp4A, and Rsp4C
Rsp3A	TTHERM_01044600	RS1	Cryo-ET analyses:<br>• a subpopulation of RS1 spokes is completely or partly missing in RSP3A-KO cilia<br>• there are no defects in RS2 and RS3 spokes in RSP3A-KO cilia<br>Cross-link data:<br>• Rsp3A cross-links with Rsp3B and Rsp14
Rsp3B	TTHERM_00566810	RS1<br>RS2	Cryo-ET analyses:<br>• a subpopulation of RS1 spokes is missing in RSP3B-KO cilia<br>• all RS2 spokes are eliminated in RSP3B-KO cilia<br>• there are no defects in RS3 spokes<br>Cross-link data:<br>• Rsp3B cross-links with Rsp3A, RSP9, Rsp22, and Cfap253
Rsp3C	TTHERM_00418270	RS2	Cryo-ET analyses:<br>• a subpopulation of RS2 spokes is completely or partly missing in RSP3C-KO cilia<br>• there are no defects in RS1 and RS3 spokes
Rsp4A	TTHERM_00427590	RS1<br>RS2	Comparative global ciliome analyses:<br>• the level of Rsp4A is reduced in cells with knocked out *RSP3B* gene<br>Co-IP and BioID data:<br>• Rsp4A co-precipitates with Rsp2, Rsp3 paralogs, Rsp4A, Rsp4C, Rsp9, and Rsp10<br>• Rsp4A is biotinylated in cells expressing BirA*-tagged Rsp3A, Rsp3B, Rsp4B, Rsp4C<br>• Rsp4A-HA-BirA* biotinylates Rsp2, Rsp3 paralogs, Rsp4 paralogs, Rsp7A, Rsp8, Rsp9, Rsp10, Rsp14, Rsp15<br>Cross-link data:<br>• Rsp4A cross-links with Rsp1, Rsp2, Rsp4B, and Rsp4C
Rsp4B	TTHERM_00502580	Rsp3C-containing RS2 subtype	Comparative global ciliome analyses:<br>• the level of Rsp4B is reduced in cells with knocked out *RSP3C* gene<br>Co-IP and BioID data:<br>• Rsp4B co-precipitates with Rsp2, Rsp3 paralogs, Rsp4A, Rsp4C, Rsp9, and Rsp10<br>• Rsp4B is biotinylated in Rsp4A-HA-BirA* cells<br>• Rsp4B-HA-BirA* biotinylates Rsp2, Rsp4A, Rsp9, Rsp12A
Rsp4C	TTHERM_00444180	RS1<br>RS2	Comparative global ciliome analyses:<br>• the level of Rsp4C is reduced in cells with knocked out *RSP3B* gene<br>Co-IP and BioID data:<br>• Rsp4C co-precipitates with Rsp3A and Rsp4A.<br>• Rsp4C is biotinylated in cells expressing BirA*-tagged Rsp3A, Rsp3B, and Rsp4A<br>• Rsp4C-HA-BirA* biotinylates Rsp2, Rsp4A, Rsp9, Rsp10, and Rsp14<br>Cross-link data:<br>• Rsp4C cross-links with Rsp2, Rsp4A, and Rsp16A
Rsp7A	TTHERM_00670610	RS1<br>RS2	Comparative global ciliome analyses:<br>• the level of Rsp7A is reduced in *RSP3B* and *CFAP91* knockouts<br>Co-IP and BioID data:<br>• Rsp7A co-precipitates with Rsp3A, Rsp4A, Rsp4B

			• Rsp7A is identified among proteins biotinylated in cells expressing BirA*-tagged protein: Rsp3A, Rsp3B, Rsp3C, Rsp4A, Rsp4C<br>• Rsp7A is not biotinylated in cells expressing RS3 protein, Cfap61-HA-BirA*<br>Cross-link data:<br>• Rsp7A cross-links with Rsp14 and Rsp22
Rsp7B	TTHERM_00492840	RS3	Comparative global ciliome analyses:<br>• the level of Rsp7B is reduced only in *CFAP91* knockouts<br>Co-IP and BioID data:<br>• Rsp7B is biotinylated in cells expressing Cfap61-HA-BirA*, Lrrc23A, Lrrc23B
Rsp8	TTHERM_00313520	RS2	Comparative global ciliome analyses:<br>• the level of Rsp8 is reduced in *RSP3B*, RSP3C, and *CFAP206* knockouts (mutants with RS2 defects)
Rsp9	TTHERM_00430020	RS1<br>RS2	Comparative global ciliome analyses:<br>• the level of Rsp9 is reduced in *RSP3B* knockout<br>Co-IP and BioID data:<br>• Rsp9 is effectively biotinylated in cells expressing BirA*-tagged protein: Rsp3 and Rsp4 paralogs<br>• Rsp9 is not or poorly biotinylated in cells expressing RS3 protein, Cfap61-HA-BirA*<br>• Rsp9 co-precipitates with Rsp3A, Rsp4A, and Rsp4B<br>Cross-link data:<br>• Rsp9 cross-links with Rsp3B
Rsp10	TTHERM_00378600	RS1<br>RS2	Comparative global ciliome analyses:<br>• the level of Rsp10 is reduced in *RSP3B* knockout<br>Co-IP and BioID data:<br>• Rsp10 is effectively biotinylated in cells expressing BirA*-tagged protein: Rsp3 and Rsp4 paralogs<br>• Rsp10 is not biotinylated in cells expressing RS3 protein, Cfap61-HA-BirA*<br>• Rsp10 co-precipitates with Rsp4A and Rsp4B
Rsp11	TTHERM_00540090	RS1<br>RS2	Comparative global ciliome analyses:<br>• the level of Rsp11 is reduced in all *RSP3* knockout mutants<br>• the level of Rsp11 is unaffected in CFAP91-KO mutant<br>Co-IP and BioID data:<br>• Rsp11 is biotinylated in cells expressing BirA*-tagged protein: Rsp3A, Rsp3B, Rsp4A,<br>• Rsp11 is not biotinylated in cells expressing RS3 protein, Cfap61-HA-BirA*
Rsp12A	TTHERM_01018330	RS2	Comparative global ciliome analyses:<br>• The level of Rsp12A is reduced in *RSP3B*, *RSP3C*, and *CFAP206* knockouts (mutants with RS2 defects)<br>Co-IP and BioID data:<br>• Rsp12A is biotinylated in cells expressing Rsp4B-HA-BirA*
Rsp12B	TTHERM_00466090	RS1<br>RS2 (?)	Comparative global ciliome analyses:<br>• The level of Rsp12B is reduced in *RSP3A* and *RSP3B* knockouts
Rsp14	TTHERM_000773538	RS1	Comparative global ciliome analyses:<br>• The level of Rsp14 is reduced in *RSP3A* and *RSP3B* knockouts<br>• The level of Rsp14 is not significantly altered in *RSP3C* and *CFAP206* mutants<br>Co-IP and BioID data:<br>• Rsp14 co-precipitates with Rsp3A,<br>Cross-link data:<br>• Rsp14 cross-links with Rsp3A, Rsp7A, and Rsp22
Rsp15	TTHERM_00695730		Comparative global ciliome analyses:<br>• The level of Rsp15 is reduced in *RSP3B*, RSP3C, and *CFAP206* knockouts (mutants with RS2 defects), and in CFAP91-KO mutant (likely due to RS2 base defect)<br>Co-IP and BioID data:<br>• Rsp15 is biotinylated in cells expressing Rsp3B, Rsp4A, or Cfap206 tagged with BirA*<br>• Rsp15 is not biotinylated in Rsp3A-HA-BirA* and Cfap61-HA-BirA* cells
Rsp16A	TTHERM_00471260	RS1<br>Rsp3B-containing RS2 subtype	Comparative global ciliome analyses:<br>• The level of Rsp16A is reduced in *RSP3A* and *RSP3B* knockouts<br>Co-IP and BioID data:<br>• Rsp16A is biotinylated in cells expressing BirA* tagged Rsp3A, Rsp3B, Rsp4A<br>Cross-link data:<br>• Rsp16A cross-links with Rsp4C
Rsp16B	TTHERM_00238810	Rsp3C-containing RS2 subtype	Comparative global ciliome analyses:<br>• The level of Rsp16B is reduced in *RSP3C*, *CFAP206* (mutants with RS2 defects), and *CFAP91* knockouts (also defects in RS2 base)<br>Co-IP and BioID data:<br>• Rsp16B is biotinylated in cells expressing Rsp4A-HA-BirA*

Rsp20/CaM1	TTHERM_00630500	RS2	Comparative global ciliome analyses:<br>• The level of Rsp20 is reduced in *RSP3B* and *RSP3C* knockouts<br>Co-IP and BioID data:<br>• Rsp20 is biotinylated in cells expressing BirA*-tagged Rsp3B and Rsp4A
Rsp22/LC8	TTHERM_000649439	RS1<br>RS2	Comparative global ciliome analyses:<br>• The level of Rsp22 is diminished in *RSP3B* and *RSP3C* knockouts<br>Co-IP and BioID data:<br>• Rsp22 co-precipitates with Rsp3A and Rsp4A<br>• Rsp22 is biotinylated in cells expressing BirA* tagged either Rsp3,<br>• Not biotinylated in cells expressing Cfap61-HA-BirA*<br>Cross-link data:<br>• Rsp22 cross-links with Rsp3B, Rsp7A, Rsp14, and Rsp15
Rsp23/Cfap67A	TTHERM_000372529	RS1<br>RS2	Co-IP and BioID data<br>• Rsp23 is biotinylated in cells expressing BirA* tagged either Rsp3B, Rsp4A<br>• Not biotinylated in cells expressing BirA*-tagged Cfap61 or Cfap91<br>• Co-precipitates with Rsp3A, Rsp4A, Rsp4B
Cfap61	TTHERM_00641200	RS3	Cryo-ET analyses:<br>– fragment of RS3 spoke is missing in CFAP61-KO cells [17]
Cfap251	TTHERM_01262850	RS3	Cryo-ET analyses:<br>• fragment of the an arch-like structure at the RS3 base is missing in CFAP251-KO cells [17]
Cfap91	TTHERM_00578560	RS3	TEM analyses:<br>of CFAP91-KO mutant showing a lack of some RS3 [18]
Cfap206	TTHERM_00820660	RS2	Cryo-ET analyses:<br>– either entire RS2 or is base and in some cases also RS3 base is missing in CFAP206-KO cells [19]
Cfap253	TTHERM_00316930	RS1	Co-IP and BioID data:<br>• Cfap253 co-precipitates with Rsp3A<br>• Cfap253 is biotinylated in cells expressing Rsp3A-HA-BirA* and Rsp3B-HA-BirA*<br>Cross-link data:<br>• Cfap253 cross-links with Rsp3B<br>• IQUB data [20]
Cfap207	TTHERM_00529880	RS2	Comparative global ciliome analyses:<br>• The level of Cfap207 is reduced in *RSP3B, CFAP206*, and *CFAP91* knockouts
Cfap198A	TTHERM_01092470	RS1, RS2	Comparative global ciliome analyses:<br>• Level diminished in *RSP3A* and *RSP3B* knockouts
Cfap198B	TTHERM_00476710	Rsp3C-containing RS2 subtype	Comparative global ciliome analyses:<br>• Level diminished in *RSP3C*
TtTpr	TTHERM_00623040	RS2	Comparative global ciliome analyses:<br>• The level of TtTpr is reduced in *RSP3C* and *CFAP206* knockouts<br>Co-IP and BioID data:<br>• TtTpr is biotinylated in cells expressing Cfap206-HA-BirA*<br>Cross-link data:<br>• TtTpr cross-links with Ak8A assigned to RS2
Kelch-motif protein	TTHERM_00760390	RS3	Comparative global ciliome analyses:<br>• The level of TtKelch is reduced in *CFAP91* knockout<br>Cross-link data:<br>• Kelch-motif proteins cross-links with Cfap91
Lrrc23A	TTHERM_000703669	RS3	Comparative global ciliome analyses:<br>• The level of Lrrc23A is reduced in *CFAP91* knockout<br>BioID data:<br>• Lrrc23A-HA-BirA* biotinylates primary RS3 proteins Cfap61 and Cfap251
Lrrc23B	TTHERM_00105260	RS3	Comparative global ciliome analyses:<br>• The level of Lrrc23B is reduced in *CFAP91* knockout<br>BioID data:<br>• Lrrc23B-HA-BirA* biotinylates primary RS3 proteins Cfap61 and Cfap251
Lrrc	TTHERM_01084360	RS3 vicinity?	Comparative global ciliome analyses:<br>• The level of Lrrc is reduced in *RSP3B* and *CFAP91* knockouts
Lrrc	TTHERM_00046820	RS3	Comparative global ciliome analyses:<br>• The level of Lrrc is reduced in *CFAP61* and *CFAP91* knockouts<br>Cross-link data:<br>• protein cross-links with Ak7A, Ak9, and DYH24
RIIa domain	TTHERM_00537370	RS3	Comparative global ciliome analyses:<br>• The level of RIIa domain is reduced in *CFAP91* knockout<br>Co-IP and BioID data:<br>• Protein is biotinylated in cells expressing BirA*-tagged, Lrrc23A, Lrrc23B
MRNN04	TTHERM_00324550	RS3	Comparative global ciliome analyses:<br>• The level of MRNN04 is reduced in *CFAP91* knockout<br>Co-IP and BioID data:

			• MRNN04 is biotinylated in cells expressing BirA*-tagged Cfap61, Cfap91, Lrrc23A, Lrrc23B
Stpg1	TTHERM_00549640	RS1<br>RS2	Comparative global ciliome analyses:<br>• The level of Stpg1 is reduced in *RSP3A*, *RSP3B*, *CFAP206*, and *CFAP91* knockouts
Stpg2	TTHERM_00420710	RS2	Comparative global ciliome analyses:<br>• The level of Stpg2 is reduced in *RSP3B* and *CFAP206* knockouts
adenylate kinase 1, Ak1	TTHERM_00317200	RS1<br>RS2	Comparative global ciliome analyses:<br>• The level of AK1 is reduced in *RSP3B* and *CFAP91* knockouts<br>Co-IP and BioID data:<br>• AK1 co-precipitates with Rsp3A, Rsp4A, Rsp4B, and Rsp4C<br>• AK1 is biotinylated in cells expressing BirA*-tagged Rsp3 and Rsp4 paralogs<br>Cross-link data:<br>• AK1 cross-links with Rsp4B, Rsp4C, and Rsp10
adenylate kinase 7A, Ak7A	TTHERM_00558060	RS3	Comparative global ciliome analyses:<br>• The level of AK7A is reduced in *CFAP91* knockout<br>Cross-link data:<br>• AK7A cross-links with AK9
adenylate kinase 7B, Ak7B	TTHERM_000569069	RS3	Comparative global ciliome analyses:<br>• The level of AK7B is reduced in *CFAP91* knockout<br>Cross-link data:<br>• AK7B cross-links with AK9
adenylate kinase 8A, Ak8A	TTHERM_00227800	R2	Comparative global ciliome analyses:<br>• The level of AK8A is reduced in *RSP3B* knockout<br>Cross-link data:<br>• AK8A cross-links with Rsp3B, Rsp16A, TtTpr
adenylate kinase 8B, Ak8B	TTHERM_00455540	RS1<br>RS2	Comparative global ciliome analyses:<br>• The level of AK8B is reduced in *RSP3A* knockout<br>Co-IP and BioID data:<br>• AK8B is biotinylated in cells expressing BirA*-tagged Rsp3A and Rsp3B<br>Cross-link data:<br>• AK8B cross-links with Rsp16A
adenylate kinase 9, Ak9	TTHERM_00148750	RS3	Comparative global ciliome analyses:<br>• The level of AK9 is reduced in *CFAP91* knockout<br>Cross-link data:<br>• AK9 cross-links with AK7A and AK7B
caseine kinase1, Ck1,	TTHERM_00938880	RS3	Comparative global ciliome analyses:<br>• The level of CK1 is reduced in *CFAP61* and *CFAP91* knockout
Guanylate kinase, Gk1	TTHERM_00781030	RS3	Comparative global ciliome analyses:<br>• The level of Gk1 is reduced in *CFAP91* knockout<br>Co-IP and BioID data:<br>• GK1 is biotinylated in cells expressing BirA*-tagged Cfap61 and Cfap91
Phosphodiesterase, Pde	TTHERM_00293350	RS3	Comparative global ciliome analyses:<br>• The level of Pde is reduced in *CFAP61* and *CFAP91* knockouts<br>Co-IP and BioID data:<br>• PdeB is biotinylated in cells expressing BirA*-tagged Lrrc23B
PKA catalytic subunit-1	TTHERM_00433420	RS2?	Comparative global ciliome analyses:<br>• The level of PKA catalytic sub-1 is reduced in *RSP3B*, *RSP3C*, and *CFAP91* knockout<br>Cross-link data:<br>• PKA catalytic sub-1 cross-links with PKA catalytic sub-1 and PKA regulatory sub
PKA catalytic subunit-2	TTHERM_00658860	RS2	Comparative global ciliome analyses:<br>• The level of PKA catalytic sub-2 is reduced in *RSP3B* knockout<br>Cross-link data:<br>• PKA catalytic sub-2 cross-links with PKA catalytic sub-2, PKA regulatory sub, and Rsp15
PKA regulatory protein	TTHERM_00623090	RS2	Comparative global ciliome analyses:<br>• The level of PKA regulatory sub is reduced in *RSP3B*, *RSP3C*, and *CFAP91* knockout<br>Cross-link data:<br>• PKA regulatory sub cross-links with PKA catalytic sub-1 and -2, and AK8B

Supplementary Table 11. Central apparatus components identified among proteins biotinylated in cilia of cells expressing BirA*-tagged Rsp.

TGD number	Protein name	CA projection	BirA*-tagged protein												
			Rsp3A	Rsp3A	Rsp3A	Rsp3B	Rsp3B	Rsp3B	Rsp3C	Rsp4A	Rsp4A	Rsp4C	Lrrc23A*	Lrrc23B*	Lrrc23B
TTHERM_00430030	Pf6	C1a						1/1			1/1		25/18	35/24	
TTHERM_00924250	Ccdc180/Cfap76	C1c/d											17/14	14/12	
TTHERM_00705200	Cfap46	C1d	25/24	18/9	21/11	1/1			4/4	29/28		55/39	59/38	140/60	17/11
TTHERM_00049190	Cfap54	C1d	4/4						4/2	7/7		29/22	139/81	216/107	19/12
TTHERM_00530270	Cfap74	C1d										1/1	18/14	23/15	
TTHERM_00189530	Cfap221	C1d							1/1			3/3	18/9	25/14	1/1
TTHERM_01142770	Spef2A C1b	C1b									2/2		15/13	17/15	1/1
TTHERM_00205170	Tt170 C1b	C1b	1/1		1/1								2/2	6/4	
TTHERM_00290850	Androglobin	C1b											48/29	28/20	1/1
TTHERM_00497260	Cfap70	C2a											10/8	15/10	
TTHERM_00551040	Hydin	C2b	3/3							1/1		9/9	200/112	268/122	5/5
TTHERM_000495990	Cfap47	C2b											263/107	91/56	

*TurboID

Supplementary Table 12. Comparative analyses of the levels of IDAs components in *RSP3* mutants

Protein name	Numer in TGD	RSP3A-KO/WT				RSP3B-KO/WT				RSP3C-KO/WT			
		LFQ		TMT		LFQ		TMT		LFQ		TMT	
		q-value	difference	q-value	difference	q-value	difference	q-value	difference	q-value	difference	q-value	difference
DYH6	TTHERM_00688470	0,427619	-0,63	0,97248	0,00	0,829776	-0,11	0,0333644	0,19	0,42251	-0,58	0,22074	0,14
DYH7	TTHERM_00912290	0,63228	-0,26	0,92333	0,01	0,889118	0,06	0,0436144	0,17	0,552849	-0,36	0,25214	0,14
DYH8	TTHERM_00531870	nd		0,35423	-0,09	nd		0,794792	0,02	nd		0,05891	0,27
DYH9	TTHERM_00947430	0,212183	-2,35	**0,01518**	**-0,47**	0,19376	-1,15	0,138485	-0,11	0,202845	-2,54	0,13222	-0,19
DYH10	TTHERM_00420340	nd		0,24242	-0,12	nd		**0,0211327**	**-0,26**	nd		0,78056	-0,03
DYH11	TTHERM_00252430	0,278289	-1,14	0,64207	0,04	0,079717	-1,13	0,695276	-0,02	0,207409	-1,33	0,61464	-0,05
DYH12	TTHERM_00919540	0,391149	-0,81	0,77556	-0,03	**0,016857**	**-3,02**	**0,0024172**	**-0,39**	0,216267	-1,14	0,89791	-0,01
DYH14	TTHERM_00492830	0,627178	-0,26	0,86334	-0,02	0,81443	0,11	0,0682636	0,15	0,604414	-0,28	0,36991	0,10
DYH15	TTHERM_00433800	0,509668	-0,39	0,53573	0,06	0,98351	-0,01	0,035494	0,19	0,58223	-0,33	0,13251	0,19
DYH16	TTHERM_00558640	0,500578	-0,41	0,49759	0,06	0,663928	-0,19	0,0255092	0,21	0,478641	-0,44	0,13177	0,20
DYH17	TTHERM_00850620	nd		0,16265	-0,15	nd		0,322829	-0,06	nd		0,56919	0,06
DYH18	TTHERM_00047540	nd		nd		nd		nd		nd		nd	
DYH19	TTHERM_01027670	0,59496	-0,32	0,13983	0,16	0,650933	-0,20	0,0160624	0,26	0,456762	-0,57	0,1503	0,17
DYH20	TTHERM_00821980	nd		0,27734	-0,11	nd		0,546651	0,04	nd		0,04042	0,29
DYH22	TTHERM_00565600	0,739417	-0,17	0,89428	0,01	0,80461	-0,12	0,0433816	0,18	0,728791	-0,18	0,63557	0,05
DYH23	TTHERM_00355100	nd		0,568953	-0,05	nd		0,716902	0,02	nd		0,36156	0,12
DYH24	TTHERM_00193520	0,454189	-0,60	0,04083	0,26	0,0565	-1,13	0,261682	0,07	0,306765	-0,88	0,23358	0,15
DYH25	TTHERM_00774820	0,497702	-0,46	0,29773	0,11	**2,36E-05**	**-4,25**	**0,004094**	**-0,77**	0,227139	-1,28	0,19224	-0,16
p28A	TTHERM_00841210	0,62273	0,34	0,13922	0,17	0,140637	-1,09	0,574056	0,04	0,529654	-0,45	0,28815	0,11
p28B	TTHERM_00319990	0,918872	0,05	0,71511	0,04	**0,186438**	**-0,70**	**0,0378964**	**-0,20**	0,546171	-0,42	0,43061	0,11
p28C	TTHERM_01129720	0,931915	0,05	0,8939	0,013	0,978215	-0,016	0,0700761	0,16	0,478333	-0,44	0,59091	0,07

Significant changes are marked in red; TGD – Tetrahymena Genome Database; LFQ – label-free quantification, TMT- tandem mass tag 10-plex isobaric mass tagging approaches; (nd, not detected) in analyzed ciliomes

Supplementary Table 13. List of primers used in this study. The nucleotide sequences recognized by the restriction endonucleases are in bold.

Primer name	Nucleotide sequence
	**Native locus expression** (-3HA and/or –HA-BirA*)
RSP3A-cod-MluI-F	AAAT**ACGCGT**AG GAGGAA CACAAT AGCAGT ATAA
RSP3A-cod-BamHI-R	AATT**GGATCC**CT CTGAAG GTAAAT TAAACC CAAAAT C
RSP3A-3UTR-PstI-F	TTAA**CTGCAG**CT TAAAGA ATAATG GATTTG TAGAGA TATTG
RSP3A-3UTR-XhoI-R	TTAA**CTCGAG**AT CATGTT TAAACT GATTTT AATAGT TATCAA A
RSP3B-cod-MluI-F	AAAT**ACGCGT**GT CTGAGG GCTCTA TTGATT TA
RSP3B-cod-BamHI-R	AATT**GGATCC**GT ATGATT TCATAT CTAAAA CAGGGA AA
RSP3B-3UTR-PstI-F	AAAT**CTGCAG**CA TATTTC TTTGCA AAATGT TTTATA AATTAG
RSP3B-3UTR-XhoI-R	AATT**CTCGAG**GT TGGATT TACAGC CAATCT AG
RSP3C-cod-MluI-F	AAAT**ACGCGT**CA ATAACC TCCAAG TGAAGA ATCA
RSP3C-cod-BamHI-R	AATT**GGATCC**AT CTTCTT CGGGTT CTTCCT CACCTT CTTCAC C
RSP3C-3UTR-PstI-F	AAAT**CTGCAG**CA TATTCT AATAAT CTTGAA TGCATG AATA
RSP3C-3UTR-XhoI-R	AATT**CTCGAG**AC TCTCTC CATCAC AAAAAT ATCTG
Bir-RSP4A-5UTR-SacII-F	AAAA**CCGCGG**GA TATCAA CAATTT TCTGAT ATTGAA TGTTGT AG
Bir-RSP4A-5UTR-PstI-R	AATT**CTGCAG**GT TCTCTT CTTTGA TTATTT ATTTTC ACTA
RSP4A-cod-MluI-F	AAAT**ACGCGT**CG TACTTT AATTGA CCCTGA TGAGA
RSP4A-cod-BamHI-R	AATT**GGATCC**AT TCTCTT CCTCTT CTTCTT ATTATT GCTATT GG
RSP4A-3UTR-PstI-F	AAAT**CTGCAG**CT ATCTAT CAATCG ATATATGTATGTATGGTT TC
RSP4A-3UTR-XhoI-R	AATT**CTCGAG**AA TATAAA AGTGCA AACGAA ATCATTAATTTTG
Bir-RSP4B-5UTR-SacII-F	TTAA**CCGCGG**GA TTTTTA AGATAA TGAAAC GTTATT TTATGC TC
Bir-RSP4B-5UTR-PstI-R	AATT**CTGCAG**TT AGAGAG TTGTTT CATTTC AATTAT TAATTC TA
RSP4B-cod-MluI-F	AAAT**ACGCGT**GT GAAAAT GACATA TAACTT GAAGAT GAAC
RSP4B-cod-BamHI-R	AATT**GGATCC**AT TTTCTT CTTCAT CTTCTT CCTTCT TTT
RSP4B-3UTR-PstI-F	AAAT**CTGCAG**TTTATTGTCAATAATTAACTATCAATATCAAAAAG
RSP4B-3UTR-XhoI-R	ATAT**CTCGAG**GA ATTATT GATGAA GAAGAG TATGAGATTATGC
Bir-RSP4C-5UTR-SacII-F	TTAA**CCGCGG**GA TATCTT ACACTT TAAATG GCTTGA AAATTA ATTG
Bir-RSP4C-5UTR-PstI-R	AATT**CTGCAG**GA TATAAA GAAGAA ACGTTA TAAATT TTGACT ATT
RSP4C-cod-MluI-F	ATTT**ACGCGT**GT ATTAAG CTTTGG ATGACC CTG
RSP4C-cod-BamHI-R	AATT**GGATCC**TTCTTATTCTTCTTAAGCATTCTCTT CATTTT CTTC
RSP4C-3UTR-PstI-F	AAAT**CTGCAG**GCTTTAAATACATATTAAATACATGTAATTATAAC
RSP4C-3UTR-XhoI-R	AATT**CTCGAG**AT ATCACT GTAAAC CTTGTA ATATTA CTAG
CFAP61-cod-MluI-F	AATT**ACGCGT**GA TGATAC CCAAAG TCGTGA TG
CFAP61-cod-BamHI-R	AATT**GGATCC**AT CTACAT TTACCT TCTTAG GAGGTA CATA
CFAP61-3UTR-PstI-F	AATT**CTGCAG**CA AACTAA ACATGT TACTCC TATTGT T
CFAP61-3UTR-XhoI-R	AATT**CTCGAG**CA TTCAAT ACTTAA CAGGAG AAATCT TC
CFAP91-cod-MluI-F	AATT**ACGCGT**GT CCTGCA ACTCCT ACTTG
CFAP91-cod-BamHI-R	AATT**GGATCC**AT TCTAAA CATTTG CGTGCT TATTTG
CFAP91-3UTR-PstI-F	AATT**CTGCAG**GA GGAGTA AGTAAC CAACAA ACC
CFAP91-3UTR-XhoI-R	AATT**CTCGAG**TC TCTAAT TCTAAA TTCCAG CTTTCA G
CFAP206-cod-MluI-F	AAAT**ACGCGT**GT TTATTG CTACCA GGTAAA CCT
CFAP206-cod-BamHI-R	AATT**GGATCC**AT TAGTGT CTTTGT CTCTTA ATCCAG T
CFAP206-3UTR-PstI-F	AAAT**CTGCAG**CCTTTCCAACTTTATATCAGATATAATTTATT ACC
CFAP206-3UTR-XhoI-R	AATT**CTCGAG**AAGCTTATTACAGTTTTAATAATTCCATCTTAGCC
LRRC23A-cod-MluI-F	AAAT**ACGCGT**TA TGAGTA AAAAAG AAAAGA AGCCAG
LRRC23A-cod-BamHI-R	AATT**GGATCC**AT TTTCTT CTTGCT ATTCTT ATTCTT GTTT
LRRC23A-3UTR-PstI-F	AAAT**CTGCAG**TA TTTACT GTAAAA TTTAGT CAGTAC ATCA
LRRC23A-3UTR-XhoI-R	AATT**CTCGAG**CT CGTTTT ACTTCT TGAATT TCTATA AATGCA T
LRRC23B-cod-MluI-F	AAAT**ACGCGT**TA TGTCAG AGTAGG AAATCG AAG

LRRC23B-cod-BamHI-R	AATT**GGATCC**TT CATCTT ACTGTT GCTAAT TTTAGG
LRRC23B-3UTR-PstI-F	AAAT**CTGCAG**AT ACATAA ATATGC AATGCA TTAAGT TATC
LRRC23B-3UTR-XhoI-R	AATT**CTCGAG**CT CTGGAT AAATTT ACTTCA ATTTGC TCAA
Gene knock-out	
RSP3A-KO-5-F-ApaI	AATT**GGGCCC**AA AGTATC AATGAA CACTTA ATTAGA ATAGC
RSP3A-KO-5-R-SmaI	AATT**CCCGGG**TG CTTATT CATATT CTTCAA TGAGCT C
RSP3A-KO-3-F-PstI	AATT**CTGCAG**AG CTCATT GTGAGT TTGCCG ATATTA CAG
RSP3A-KO-3-R-SacII	AATT**CCGCGG**CA ATATCT CTACAA ATCCAT TATTCT TTAAG
RSP3B-KO-5-F-ApaI	AATT**GGGCCC**CT AGTATT TAGTTA AACAAG GCATGC
RSP3B-KO-5-R-SmaI	AATT**CCCGGG**AC GAGGAT TAGGTC TGTCAA TAA
RSP3B-KO-3-F-PstI	AATT**CTGCAG**GC AGCAAG AATTCT AAGTAA AGCA
RSP3B-KO-3-R-SacII	AATT**CCGCGG**AG TTTTTG ATAAAG CTTCGT ATAATA TGG
RSP3C-KO-5-F-ApaI	AATT**GGGCCC**AC GCGTTA TGCAGT AAAGAA TTTTGTGTTTAG G
RSP3C-KO-5-R-SmaI	AATT**CCCGGG**AC TTATGT GTAAGC ATCAAT ACCAG
RSP3C-KO-3-F-PstI	AATT**CTGCAG**CA TATTAA CAGATT CCTTGA AGGAAC T
RSP3C-KO-3-R-SacII	AATT**CCGCGG**TT AGGAGG AATACC TTCAGT ACTG
Co-deletion primers	
RSP2-coDel-F	CAGTTC TCATCA AGTTGT AATGCT AAAATG CGGCCG CCTGCA GCTGTA TTTAAG GAGCC
RSP2-coDel-R	GGACTC TTTATT GTTATC ATCTTA TGACCG CGGCCG CCTGTT ATTCTG TTAACT AAGGCA TAAGG
Primers used to verify deletion of the fragment of RSP3 gene	
RSP3A-KO_check_R_2	TGTTTA AAACCC AAGGCA AGAAAT T
RSP3A-KO_check_R_1	TCCAGA TTTAAC TCAACA AACCTC CAA
RSP3A-KO_check_F_1	GGATGA AAATGA GATAGA GCCAGA T
RSP3B-KO_check_R_2	TGTTTC TTGATA TAAGTT TCTTGT GAGA
RSP3B-KO_check_R_1	TAAGAC AAATTC TCCAAC AAGACC A
RSP3B-KO_check_F_1	AATAAT TAACTG ACAAGC CACCTG A
RSP3C-KO_check_F_2	AAAAGA TCGTCT TCTTGA CCTCTA T
RSP3C-KO_check_R_1	CCTTGG AGAGCA TTATAC TTTGTG T
RSP3C-KO_check_F_1	GGCTCC TTATGA TATTAA GCCATC T
RSP2-coDel-spr-F	CATAGA AGCAAA GACTGA TTCCAT
RSP2-coDel-spr-R	GACTTC TTAGAG AATATT ATGCAA ATTCG

Supplementary Table 14. Source data - cells swimming rate

Supplementary Table 15. Source data – cilia beating frequency

Supplementary Table 16. Source data – co-IP data

Supplementary Table 17. Source data – BioID data

Supplementary Table 18. Source data – TMT data

Supplementary Table 19. Source data – LFQ data

Supplementary Table 20. Reagents used.

REAGENT or RESOURCE	SOURCE	IDENTIFIER
Antibodies		
Mouse monoclonal anti-HA	Biolegend	901503
Rabbit polyclonal anti-GFP	Abcam	Ab6556
Mouse monoclonal anti-α-tubulin, 12G10	DSHB	AB_1157911
Mouse monoclonal anti-acetylated (K40) α-tubulin, 6-11B1	Sigma-Aldrich	MABT868
Rabbit monoclonal anti-acetylated (K40) α-tubulin, D20G3	Cell Signaling Technology	35652

Rabbit polyclonal anti-polyglicylated tubulin	Duan and Gorovsky, 2002	doi: 10.1016/s0960-9822(02)00651-6.
Goat anti-mouse IgG-Alexa Fluor 488	Thermo Fisher Scientific	A-11029
Goat anti-mouse IgG-Alexa Fluor 555	Thermo Fisher Scientific	A-31570
Goat anti-rabbit IgG-Alexa Fluor 488	Thermo Fisher Scientific	A-11034
Goat anti-rabbit IgG-Alexa Fluor 555	Thermo Fisher Scientific	A-21429
Goat anti-mouse IgG-HRP	Jackson ImmunoResearch Laboratories	115-035-146
Goat anti-rabbit IgG-HRP	Sigma-Aldrich	401315, RRID: AB_2617117
Bacterial and virus strains		
E. coli XL-10 Gold	Agilent	200315
Chemicals, peptides, and recombinant proteins		
Pierce High Sensitivity Streptavidin-HRP	Thermo Fisher Scientific	21130
Antimycotic antibiotic	Sigma-Aldrich	A5955
Proteose peptone	Thermo Fisher Scientific	211684
Biotin	Sigma-Aldrich	B4501
Iodoacetamide	Sigma-Aldrich	I6125
Complete mini EDTA-free protease inhibitor cocktail	Roche/Sigma-Aldrich	5056489001
Paromomycin sulphate	Sigma-Aldrich	P9297
Puromycin dihydrohloride	BioShop	PUR333.500
Cycloheximide	Sigma-Aldrich	C7698
Cadmium chloride	Sigma-Aldrich	202908
Phusion™ Hot Start II DNA Polymerases	Thermo Fisher Scientific	F-549L
dNTP	EURx	E0502
Gold particles 0,5-0,8 nm	Thermo Fisher Scientific	044636.MD
Critical commercial assays		
Pierce BCA Protein Assay Kit	Thermo Fisher Scientific	23225
TMT10plex™ Isobaric Label Kit	Thermo Fisher Scientific	90406
Westar Supermova kit	Cyanagen	XLS075,0100
ReadyPrep 2D cleanup kit	Bio-Rad	1632130
Pierce peptide desalting spin columns	Thermo Fisher Scientific	89851
Pierce Quantitative Fluorescent Peptide Assay 96-Well Plates for Pierce™ Quantitative Fluorometric Peptide Assay	Thermo Fisher Scientific	88378
Experimental models: Organisms/strains		
Tetrahymena thermophila CU428.2 (wild-type strain)	Tetrahymena Stock Center	
Tetrahymena thermophila B2086.2 (wild-type strain)	Tetrahymena Stock Center	
Tetrahymena thermophila CU427.4 (wild-type strain)	Tetrahymena Stock Center	
Tetrahymena thermophila A*III (infertile strain)	Tetrahymena Stock Center	
Tetrahymena thermophila RSP3A-KO-neo4	This study	N/A
Tetrahymena thermophila RSP3B-KO-neo4	This study	N/A
Tetrahymena thermophila RSP3C-KO-neo4	This study	N/A
Tetrahymena thermophila RSP32coDel (paromomycin resistance)	This study	N/A
Tetrahymena thermophila CFAP61-KO-neo4	Urbanska et al., 2015	N/A
Tetrahymena thermophila CFAP206-KO-neo4	Vasudevan et al., 2015	N/A
Tetrahymena thermophila CFAP91-KO-neo4	Bicka et al., 2022	N/A
Tetrahymena thermophila CU428:Rsp3A-3HA-neo4	This study	N/A
Tetrahymena thermophila CU428:Rsp3B-3HA-neo4	This study	N/A
Tetrahymena thermophila CU428:Rsp3C-3HA-neo4	This study	N/A
Tetrahymena thermophila CU428:Rsp3B-3HA-neo4:Rsp3A-GFP-Pur	This study	N/A

Tetrahymena thermophila CU428:Rsp3B-3HA-neo4:Rsp3C-GFP-Pur	This study	N/A
Tetrahymena thermophila CU428:Rsp3A-BirA*-HA	This study	N/A
Tetrahymena thermophila CU428:Rsp3B-BirA*-HA	This study	N/A
Tetrahymena thermophila CU428:Rsp3C-BirA*-HA	This study	N/A
Tetrahymena thermophila CU428:Rsp4A-BirA*-HA	This study	N/A
Tetrahymena thermophila CU428:Rsp4B-BirA*-HA	This study	N/A
Tetrahymena thermophila CU428:Rsp4C-BirA*-HA	This study	N/A
Tetrahymena thermophila CU428:Cfap206-BirA*-HA	This study	N/A
Tetrahymena thermophila CU428:Cfap91-BirA*-HA	Bicka et al., 2022	N/A
Oligonucleotides		
All primers used in this study, including their sequences are listed in Table S2	Genomed	N/A
Recombinant DNA		
pNeo4	Mochizuki et al., 2008	doi: 10.1016/j.gene.2008.08.007
pPur	Iwamoto et al., 2014	doi: 10.1016/j.gene.2013.10.049
pCFAP44-TtBCCP-neo4	Fu et al., 2018	doi: 10.1091/mbc.E18-02-0142
pCFAP44-3HA-neo4	Urbanska et al., 2018	doi: 10.1007/s00018-018-2819-7
pCFAP44-HA-BirA*-neo4	Urbanska et al., 2018	doi: 10.1007/s00018-018-2819-7
pCFAP44-2V5-pPur	Urbanska et al., 2018	doi: 10.1007/s00018-018-2819-7
pRSP3A-KO-neo4	This study	N/A
pRSP3B-KO-neo4	This study	N/A
pRSP3C-KO-neo4	This study	N/A
pRSP3A-3HA-neo4	This study	N/A
pRSP3B-3HA-neo4	This study	N/A
pRSP3C-3HA-neo4	This study	N/A
pRSP3A-HA-BirA*-neo4	This study	N/A
pRSP3B-HA-BirA*-neo4	This study	N/A
pRSP3C-HA-BirA*-neo4	This study	N/A
pRSP4A-HA-BirA*-neo4	This study	N/A
pRSP4B-HA-BirA*-neo4	This study	N/A
pRSP4C-HA-BirA*-neo4	This study	N/A
pCFAP206-HA-BirA*-neo4	This study	N/A
pCFAP91-HA-BirA*-neo4	Bicka et al., 2022	doi: 10.3390/cells11244048
Software and algorithms		
Fiji ImageJ	https://fiji.sc/	N/A
Adobe Photoshop	https://www.adobe.com	N/A
ZEN2 blue edition	Carl Zeiss	N/A
Serial EM	Mastronarde 2005	N/A
Alignframes	Mastronade and Held, 2017	N/A
WARP	Tegunov and Cramer, 2019	N/A
IMOD	https://bio3d.colorado.edu/imod/	N/A
Axoneme align	Bui and Ishikawa, 2013	N/A
Relion4.0	https://relion.readthedocs.io/en/release-4.0	N/A
IsoNet	https://isonetcryoet.com/	N/A

UCSF ChimeraX	https://www.cgl.ucsf.edu/chimerax/	N/A
FragPipe v. 17.1	Nesvilab, University of Michigan, Ann Arbor, MI	N/A
MSFragger v. 3.4	Kong et al., 2017	N/A
Philosopher v. 4.2.1	da Veiga Leprevost et al., 2020	N/A
ProteoWizard's MSConvert v. 3.0.1908	Palo Alto, CA	N/A
Perseus v. 2.0.3	Max Planck Institute of Biochemistry, Martinsried, Germany	N/A

Supplementary Videos

Video 1. Cilia beating in WT *Tetrahymena* cell

Video 2. Cilia beating in RSP3A-KO *Tetrahymena* mutant

Video 3a. Cilia beating in RSP3B-KO *Tetrahymena* mutant

Video 3b. Cilia beating in RSP3B-KO *Tetrahymena* mutant

Video 4. Cilia beating in RSP3C-KO *Tetrahymena* mutant

References

1. Letunic I, Bork P (2018) 20 years of the SMART protein domain annotation resource. Nucleic Acids Res 46:D493–D496. https://doi.org/10.1093/NAR/GKX922

2. Letunic I, Khedkar S, Bork P (2021) SMART: recent updates, new developments and status in 2020. Nucleic Acids Res 49:D458–D460. https://doi.org/10.1093/NAR/GKAA937

3. Jeanson L, Copin B, Papon JF, et al (2015) RSPH3 Mutations Cause Primary Ciliary Dyskinesia with Central-Complex Defects and a Near Absence of Radial Spokes. Am J Hum Genet 97:153–162. https://doi.org/10.1016/J.AJHG.2015.05.004

4. Jeanmougin F, Thompson JD, Gouy M, et al (1998) Multiple sequence alignment with Clustal X. Trends Biochem Sci 23:403–405. https://doi.org/10.1016/S0968-0004(98)01285-7

5. Galtier N, Gouy M, Gautier C (1996) SEAVIEW and PHYLO_WIN: two graphic tools for sequence alignment and molecular phylogeny. Comput Appl Biosci 12:543–548. https://doi.org/10.1093/BIOINFORMATICS/12.6.543

6. Nicholas K.B, Nicholas H.B. (1997) GeneDoc: analysis and visualization of genetic variation. Embnew. News. 1997;4:14. EMBnet News

7. Diener DR, Ang LH, Rosenbaum JL (1993) Assembly of flagellar radial spoke proteins in Chlamydomonas: identification of the axoneme binding domain of radial spoke protein 3. J Cell Biol 123:183–190. https://doi.org/10.1083/JCB.123.1.183

8. Jivan A, Earnest S, Juang YC, Cobb MH (2009) Radial spoke protein 3 is a mammalian protein kinase A-anchoring protein that binds ERK1/2. J Biol Chem 284:29437–29445. https://doi.org/10.1074/JBC.M109.048181

9. Sivadas P, Dienes JM, Maurice MS, et al (2012) A flagellar A-kinase anchoring protein with two amphipathic helices forms a structural scaffold in the radial spoke complex. J Cell Biol 199:639–651. https://doi.org/10.1083/JCB.201111042

10. Gupta A, Diener DR, Sivadas P, et al (2012) The versatile molecular complex component LC8 promotes several distinct steps of flagellar assembly. J Cell Biol 198:115–126. https://doi.org/10.1083/JCB.201111041

11. Coyne RS, Thiagarajan M, Jones KM, et al (2008) Refined annotation and assembly of the Tetrahymena thermophila genome sequence through EST analysis, comparative genomic hybridization, and targeted gap closure. BMC Genomics 9:. https://doi.org/10.1186/1471-2164-9-562

12. Rhizobium GE (2025) UniProt: the Universal Protein Knowledgebase in 2025. Nucleic Acids Res 53:13–14. https://doi.org/10.1093/NAR/GKAE1010

13. Habicht J, Woehle C, Gould SB (2015) Tetrahymena Expresses More than a Hundred Proteins with Lipid-binding MORN Motifs that can Differ in their Subcellular Localisations. Journal of Eukaryotic Microbiology 62:694–700. https://doi.org/10.1111/JEU.12216

14. Yang P, Diener DR, Yang C, et al (2006) Radial spoke proteins of Chlamydomonas flagella. J Cell Sci 119:1165–1174. https://doi.org/10.1242/JCS.02811

15. McCafferty CL, Papoulas O, Lee C, et al (2024) An amino acid-resolution interactome for motile cilia identifies the structure and function of ciliopathy protein complexes. Dev Cell 60:965-978.e3. https://doi.org/10.1016/J.DEVCEL.2024.11.019

16. Gui M, Ma M, Sze-Tu E, et al (2020) Structures of radial spokes and associated complexes important for ciliary motility. Nature Structural & Molecular Biology 2020 28:1 28:29–37. https://doi.org/10.1038/s41594-020-00530-0

17. Urbanska P, Song K, Joachimiak E, et al (2015) The CSC proteins FAP61 and FAP251 build the basal substructures of radial spoke 3 in cilia. Mol Biol Cell 26:1463–1475. https://doi.org/10.1091/MBC.E14-11-1545/MC-E14-11-1545-S12.MPG

18. Bicka M, Joachimiak E, Urbanska P, et al (2022) Cfap91-Dependent Stability of the RS2 and RS3 Base Proteins and Adjacent Inner Dynein Arms in Tetrahymena Cilia. Cells 11:4048. https://doi.org/10.3390/CELLS11244048/S1

19. Vasudevan KK, Song K, Alford LM, et al (2015) FAP206 is a microtubule-docking adapter for ciliary radial spoke 2 and dynein c. Mol Biol Cell 26:696–710. https://doi.org/10.1091/MBC.E14-11-1506

20. Zhang X, Xiao Z, Zhang J, et al (2022) Differential requirements of IQUB for the assembly of radial spoke 1 and the motility of mouse cilia and flagella. Cell Rep 41:111683. https://doi.org/10.1016/J.CELREP.2022.111683
